# Uncovering the ceRNA Network Related to the Prognosis of Stomach Adenocarcinoma Among 898 Patient Samples

**DOI:** 10.1007/s10528-023-10656-7

**Published:** 2024-02-15

**Authors:** Zhe Liu, Fang Liu, Olutomilayo Olayemi Petinrin, Fuzhou Wang, Yu Zhang, Ka-Chun Wong

**Affiliations:** 1 Department of Computer Science, City University of Hong Kong, Hong Kong, China; 2 College of Chemistry and Chemical Engineering, Central South University, Changsha, China; 3 College of Life Sciences, Xinyang Normal University, Xinyang, China

**Keywords:** Stomach adenocarcinoma (STAD), Competing endogenous RNA (ceRNA), Prognosis, Long noncoding RNA (lncRNA), Risk model, Cox regression

## Abstract

Stomach adenocarcinoma (STAD) patients are often associated with significantly high mortality rates and poor prognoses worldwide. Among STAD patients, competing endogenous RNAs (ceRNAs) play key roles in regulating one another at the post-transcriptional stage by competing for shared miRNAs. In this study, we aimed to elucidate the roles of lncRNAs in the ceRNA network of STAD, uncovering the molecular biomarkers for target therapy and prognosis. Specifically, a multitude of differentially expressed lncRNAs, miRNAs, and mRNAs (i.e., 898 samples in total) was collected and processed from TCGA. Cytoplasmic lncRNAs were kept for evaluating overall survival (OS) time and constructing the ceRNA network. Differentially expressed mRNAs in the ceRNA network were also investigated for functional and pathological insights. Interestingly, we identified one ceRNA network including 13 lncRNAs, 25 miRNAs, and 9 mRNAs. Among them, 13 RNAs were found related to the patient survival time; their individual risk score can be adopted for prognosis inference. Finally, we constructed a comprehensive ceRNA regulatory network for STAD and developed our own risk-scoring system that can predict the OS time of STAD patients by taking into account the above.

**Supplementary Information:**

The online version contains supplementary material available at 10.1007/s10528-023-10656-7.

## Introduction

Stomach adenocarcinoma (STAD) is a devastating digestive tract disease that is prevalent across the world. As the fourth most frequent cause of cancer-related deaths worldwide and the fifth most common malignancy, gastric cancer (GC) continues to be a challenge for global health (Ilic and Ilic 2022). In 2020, there were more than 1 million new cases and an estimated 769,000 deaths, with China accounting for nearly half of both (Sung et al. 2021). STAD makes up more than 95% of instances of GC (Piazuelo and Correa 2013). Age, high salt intake, alcohol, and active cigarette use are all considered critical risk factors for this condition (Yusefi et al. 2018; Poorolajal et al. 2020). The competing endogenous RNA (ceRNA) hypothesis, an as-yet-unknown theory of how gene expression is regulated, was initially put forth by Salmena in 2011 (Salmena et al. 2011). The development of STAD is strongly connected with the ceRNA regulatory network. In STAD, a prognosis-related ceRNA network has yet to be discovered systematically and comprehensively (Nie et al. 2020).

Noncoding RNAs having more than 200 nucleotides in length are referred to as long noncoding RNAs (lncRNAs), which lack the ability to code for proteins but have the power to control gene expression (Ghafouri-Fard and Taheri 2020). Short single-stranded RNAs called microRNAs (miRNAs), also known as noncoding RNAs, typically include 18–23 nucleotides. MiRNAs can limit the spread of cancer by acting as oncogenes or suppressors (Shin and Chu 2014). The decreased gene expression by specifically binding to the 3′ UTR of downstream target mRNAs. In order to restore the activity of downstream mRNAs, lncRNAs can function as ceRNAs that bind to miRNAs (Zheng et al. 2020b). Cell proliferation, migration, and invasion are some biological processes regulated by the regulatory network connecting lncRNAs, miRNAs, and mRNAs. Cell growth and development are impacted by ceRNA network disruption, which frequently results in numerous illnesses, particularly cancer (Su et al. 2021). In STAD, lncRNAs, miRNAs, and mRNAs work together rather than just interacting directly. For example, according to Huang et al. research, the *IGF2-AS-miR-503-SHOX2* ceRNA network was responsible for *IGF2-AS*’s role in promoting tumor growth and invasion (Huang et al. 2020b). Zong et al. point out that the RhoA signaling pathway allowed the *CTC-497E21.4-miR-22-3p-NET1* ceRNA network to play beneficial functions in the evolution of GC (Zong et al. 2020).

Researchers are increasingly adopting statistical algorithms to investigate novel diagnoses and therapy targets due to precision medicine’s quick progress. Exploring the relationship between genomic features and clinical characteristics was made possible because of the TCGA database source. The TCGA team has so far used integrated multi-dimensional analytics and wide-scale genome sequencing to examine large cohorts of more than 30 human tumors. Research on specific cancer types as well as thorough assessments of all cancers, has added to our understanding of carcinogenesis. For example, Li et al. constructed a ceRNA network using 15 differentially expressed mRNAs, one differentially expressed miRNA, and two differentially expressed lncRNAs using integrated analysis based on TCGA (Li et al. 2019b). Liu et al. explored the interactions between differentially expressed lncRNA, miRNA, and mRNA in TCGA and established a lncRNA-miRNA-mRNA network in clear cell renal cell carcinoma (Liu et al. 2020). Gao et al. identified early diagnostic and prognostic biomarkers for liver cancer based on studies on TCGA RNA-seq and clinical information data (Gao et al. 2021).

**Fig. 1:**
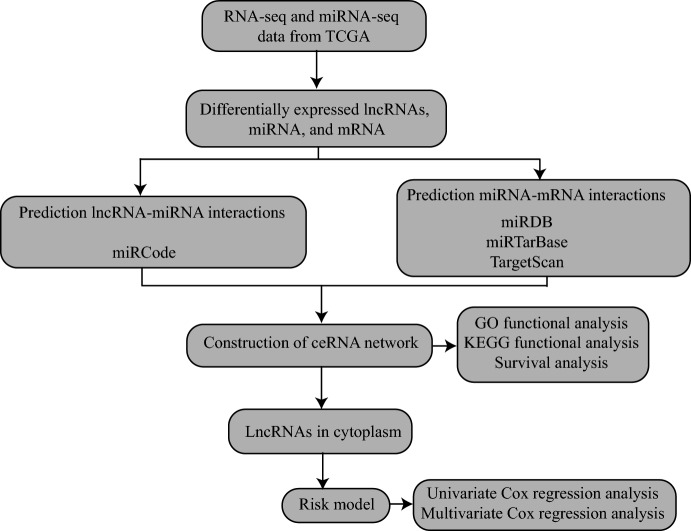
The schematic workflow of ceRNA regulatory network construction in STAD

In this study, we systematically and comprehensively identified survival-related ceRNA networks in STAD. The study of cancer from a molecular perspective has recently been a research hotspot due to advancements in diagnostic technologies and a deeper understanding of cancer gene maps. This has also led to improvements in the study of the molecular basis of treatment for patients with STAD. A flow diagram for the Construction of the ceRNA network is shown in Fig. 1.

## Materials and Methods

### Data Collection and Pre-processing

We downloaded lncRNA, miRNA, mRNA expression profile, and their clinical information data from TCGA (https://www.cancer.gov/tcga) database using TCGAbiolinks (version: 2.25.0) R package (Colaprico et al. 2016). Data pre-processing mainly completes inter-sample correlation testing (inter-group differences), count library standardization, and gene filtering. TCGAanalyze_Preprocessing() function was utilized to check and remove outliers from data using Spearman correlation coefficients. The cutoff of correlation was set to 0.6, which means we will filter these samples if their Spearman correlation values in samples are less than 0.6. Next, we only kept the tumor samples if their tumor purity values were greater than 60%. TCGAanalyze_Normalization() function was used to perform normalization, including withinLaneNormalization, betweenLaneNormalization, and quantileNormalization. TCGAanalyze_Filtering() function was utilized to remove low-expressed genes. The method of filtering was set to quantile. The parameter qnt.cut, which is a threshold selected as the mean for filtering, was set to 0.25.

For RNA-seq data (lncRNA and mRNA), we obtained 407 STAD samples (375 tumor and adjacent 32 normal samples). For miRNA-seq (miRNA), we obtained 491 STAD samples (446 tumor and 45 adjacent normal samples). The gene type information came from GENCODE (https://www.gencodegenes.org/, version: GRCh38/hg38). We defined the following six gene types as lncRNAs: sense_overlapping, lincRNA, 3prime_overlapping_ncrna, processed_transcript, antisense, and sense_intronic.

### Identification of DEGs

Differentially expressed genes (DEGs) were identified with edgeR (version: 3.38.1) R package (Robinson et al. 2010). The criteria for selecting differentially expressed lncRNAs, miRNAs, and mRNAs is |log_2_(fold change)|> 1 and FDR < 0.05. Three volcano plots were utilized to visualize DEGs (lncRNAs, miRNA, and mRNA). We treated the genes as up-regulated genes if they were significantly up-regulated in STAD tumor samples compared to adjacent normal samples, while we treated the genes as down-regulated genes if they were significantly down-regulated in STAD tumor samples compared to adjacent normal samples.

### Interaction Annotations

#### Interactions Between lncRNAs and miRNAs

The miRCode (http://www.mircode.org/) database (Jeggari et al. 2012) was used for inferring putative miRNA target sites in the lncRNA. We exported the interacted miRNAs from miRCode (version: 11, June 2012) for differentially expressed lncRNAs.

#### Interactions Between miRNAs and mRNAs

We adopted a strategy based on the vote for improving the reliability of miRNA and mRNA interactions following published methods (Bai et al. 2019; Chen et al. 2021). If an interaction occurred in twice of the following three databases, miRDB (http://www.mirdb.org/, version: 6.0, June 2019) database (Chen and Wang 2020), miRTarBase database (https://mirtarbase.cuhk.edu.cn/~miRTarBase/miRTarBase_2022/php/index.php, version: 9.0, September 2021) (Huang et al. 2020a), and TargetScan (https://www.targetscan.org/vert_80/, version: 8.0, 23 May 2022) database (McGeary et al. 2019), we picked up this interaction.

### Subcellular Localization of lncRNAs

We used the lncLocator (http://www.csbio.sjtu.edu.cn/bioinf/lncLocator/) database (Cao et al. 2018) to look into the lncRNAs’ intracellular location because they can only serve as nodes of the ceRNA network in the cytoplasm. The locLocator is an ensemble classifier-based predictor exploiting lncRNA sequence information. So, we exported the sequences from the GENCODE database’s lncRNA transcript sequence information (version: GRCh38/hg38). Then, lncRNA sequences were uploaded into the lncLocator database. The subcellular locations consist of cytoplasm, nucleus, cytosol, ribosome, and exosome in the locLocator.

### Construction of ceRNA Network

Cytoscape (http://www.cytoscape.org/, version 3.9.0) (Shannon et al. 2003) software was utilized for visualizing the locations of lncRNAs and ceRNA networks.

### Functional Enrichment Annotation of DEGs

We used DAVID (The Database for Annotation, Visualization, and Integrated Discovery, https://david.ncifcrf.gov/) to carry out GO enrichment analysis in order to investigate the role of differentially expressed mRNAs in the ceRNA network (Huang et al. 2009; Sherman et al. 2022). The chord diagrams were created using the GOplot R package (version 1.0.2). In addition, the KEGG (Kyoto Encyclopedia of Genes and Genomes, https://www.genome.jp/kegg/) signaling pathway enrichment analysis of mRNAs in the ceRNA network was carried out using the clusterProfile (version 4.4.4) package in R (Ogata et al. 1999; Kanehisa and Goto 2000; Kanehisa 2019; Kanehisa et al. 2019, 2021).

### Optimal Cutoff Selection and Kaplan–Meier Survival Analysis

There is no difference in survival analysis in many cases when using specific locations, such as median, mean, quartile, etc., as the cutoff point. At this time, it is often necessary to find an optimal cutoff point to make a difference in survival analysis. The surv_cutpoint() function in survminer (version: 0.4.9) package R was utilized to display the distribution of gene expression levels and determine the optimal cutoff for survival analysis (Li et al. 2020; Sun et al. 2021). R package survival (version: 3.3.1) was used for visualizing the difference between low- and high-expression groups classified by the obtained optimal cutoff. P-values below 0.05 were regarded as significant.

### Creation of Risk-Scoring Model

The upstream region of the ceRNA network is dominated by lncRNAs, which serve as the main miRNA and mRNA effectors (Zhang et al. 2019b; Li et al. 2019a). Additionally, lncRNAs are very particular in their expression and distribution, making them the best possible biomarkers for diagnosing and evaluating the prognosis of STAD (Chan and Tay 2018; Han et al. 2021). The survival (version: 3.3.1) R package was used to create a risk-scoring model for lncRNAs in the ceRNA network. The multivariate Cox regression model included *LINC00486* and *LSAMP-AS1* to make the independent prognostic signature for STAD (*P*-value ≤ 0.05). The following formula was used to calculate the risk score for each patient:

$$\mathrm{Risk score}= {\sum }_{i}^{n}{Exp}_{i}\times i\beta$$

here, ${Exp}_{i}$ represents the expression levels of lncRNA. $\upbeta$ represents the regression coefficient of multivariate Cox regression for lncRNA.

### Univariate and Multivariate Cox Regression

Mining the independent factors is essential for prognostic analysis for cancer research (Liu et al. 2022). Univariate Cox regression analysis was conducted to determine whether the clinical characteristics, such as age, gender, stage, tissue of origin, primary diagnosis, AJCC T, AJCC M, AJCC N, race, and risk score, were significantly associated with overall survival (OS) in STAD patients (*P*-value ≤ 0.05). Then, all clinical factors were compared at 1 time to identify independent prognostic factors (*P*-value ≤ 0.05). The coxph() function in survival (version: 3.3.1) R package was applied for univariate and multivariate Cox regression analysis. And forestplot (version: 2.0.1) R package was applied for visualizing the results.

#### Linear Regression Analysis of lncRNAs and mRNAs

The ceRNA mechanism theory states that lncRNAs interact directly with miRNAs to influence mRNA expression favorably (Zhang et al. 2021). Using R software and ggpubr (version: 0.4.0) package, linear regression analysis of the log_2_ transformed normalized the ceRNA network’s lncRNA, and mRNA expression levels were performed. The results were displayed via ggscatter() function. The cor.test() was used to evaluate the correlation. We regarded there is a statistically positive significant correlation between lncRNA and mRNA if their *P*-value ≤ 0.05 and cor > 0.3, there is a statistically negative significant correlation between lncRNA and miRNA if their *P*-value ≤ 0.05 and cor < − 0.3, and there is a statistically negative significant correlation between mRNA and miRNA if their *P*-value ≤ 0.05 and cor < − 0.3.

## Results

### Differentially Expression of lncRNA, miRNA, and mRNA

One can study the differences between tumor and adjacent normal samples to determine the genetic origin and biological pathways, therefore, identifying potential targets for treating cancer (Narrandes and Xu 2018). Identification of DEGs is vital for cancer research (Zhang et al. 2019a). From the TCGA database, we obtained the expression profiles of 2,366 lncRNAs, 1,881 miRNAs, and 19,431 mRNAs. Then, we got 1,112 lncRNAs, 587 miRNAs, and 12,855 mRNAs after the pre-processing, including inter-sample correlation testing (inter-group differences), count library standardization, and gene filtering. With the criteria of |log_2_(fold change)|> 1 and FDR < 0.05, a total of 380 differentially expressed lncRNAs, 143 differentially expressed miRNAs, and 4,344 differentially expressed mRNAs were selected.

**Fig. 2:**
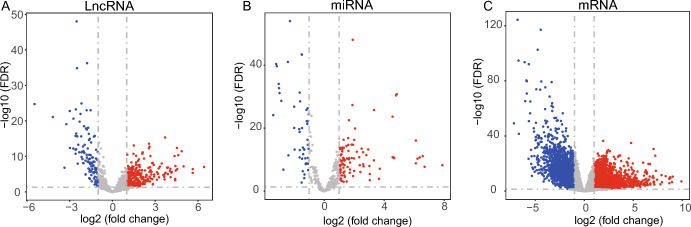
Volcano plots of differentially expressed lncRNAs (**A**), miRNAs (**B**), and mRNAs (**C**). The red dots indicate up-regulated genes, while the blue dots indicate down-regulated genes in STAD. The x-axis represents the log_2_ (fold change), while the y-axis represents the − log_10_ (FDR)

Two hundred and seventy differentially expressed lncRNAs, 95 differentially expressed miRNAs, and 2,220 differentially expressed mRNAs were among those that were up-regulated in STAD tumor samples compared to adjacent normal samples, and 110 differentially expressed lncRNAs, 48 differentially expressed miRNAs, and 2,124 differentially expressed mRNAs were among those that were down-regulated (Fig. 2A–C). They were regarded as essential genes involved in the early incidence of STAD.

### Interactions Between DE lncRNA and DE miRNA

Using the miRCode database, we first identified possible miRNAs interacting with 380 DE lncRNAs. The interacting genes between the 143 DE miRNAs and the predicted miRNAs were then identified. Finally, we discovered 26 lncRNAs and 28 miRNAs with the potential for mutual interaction (Table S1).

### Interactions Between DE mRNA and DE miRNA

By using mRNAs targeted by miRNAs shared by two out of three databases (miRDB, miRTarBase, and TargetScan), we identified target genes for the 26 miRNAs mentioned above, which increased the accuracy of the bioinformatics prediction. Then, we contrasted potential target mRNAs with 1,535 DE mRNAs that had differential expressions. Finally, the ceRNA network was established through miRNA-mRNA interaction pairs, including 16 mRNAs and three miRNAs (Table S2).

### Subcellular Localization of lncRNA and Construction of ceRNA Network

**Fig. 3:**
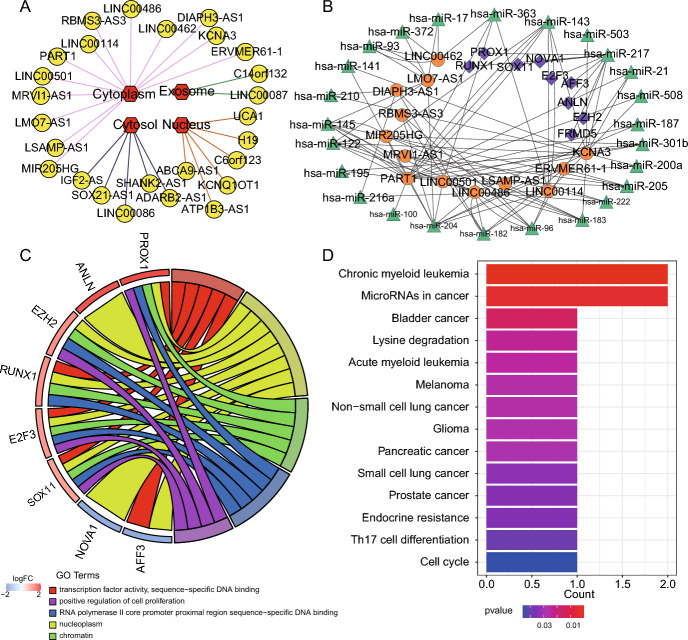
The Identification and Functional Annotation of ceRNA Network. **A** The predicted subcellular localization of lncRNAs. The red hexagon indicates the predicted location of lncRNA, and the yellow circle indicates lncRNA. **B** ceRNA network. The orange circle indicates lncRNA, the purple diamond indicates mRNA, and the green triangle indicates miRNA. **C** The chord diagram of mRNAs with GO term. The left circle indicates mRNA. For genes, the red color represents up-regulated, while the blue represents down-regulated genes in STAD. The right circle indicates GO term. **D** The barplot visualization of mRNA functional enrichment results

The endogenous competition role of lncRNAs is mainly manifested in the cytoplasm, making it imperative to examine the cytoplasmic-nuclear localization of these lncRNAs in order to understand their complex but precise regulatory processes better (Fan et al. 2019). We counted and drew the distribution of 26 lncRNAs using Cytoscape. In this study, we identified 13 lncRNAs located in the cytoplasm of the cell using lncLocator software (Fig. 3A and Table S3).

After taking into account the interactions between the remaining DEGs, a final STAD ceRNA regulatory network with 47 nodes and 98 edges was created by combining 13 lncRNAs, 25 miRNAs, and nine mRNAs. The ceRNA network was visualized by Cytoscape software (Fig. 3B).

### GO Term and KEGG Pathway

The nine mRNAs in the ceRNA regulatory network were investigated concerning their putative biological functions and pathways. We conducted GO functional enrichment analysis on the DAVID database and reported significant enriched GO terms. There are 18 significant GO terms enriched by mRNAs in the ceRNA network (*P*-value ≤ 0.05, Table S4). We used a chord figure to visualize the enrichment results (Fig. 3C). The top five GO terms among these were “transcription factor activity, sequence-specific binding,” “nucleoplasm,” “chromatin,” “RNA polymerase II core promoter proximal region sequence-specific DNA binding,” and “positive regulation of cell proliferation.”

KEGG was also selected for annotation of the function of mRNAs in the ceRNA network. The R package clusterProfiler was utilized to perform gene enrichment analysis and visualize the results (Fig. 3D). mRNAs in the ceRNA network enriched in 14 significant KEGG signaling pathways (*P*-value ≤ 0.05). The top five KEGG pathways among these were “Chronic myeloid leukemia,” “MicroRNAs in cancer,” “Bladder cancer,” “Lysine degradation,” and “Acute myeloid leukemia.”

### Survival Curves of ceRNA Network-Related Genes

**Fig. 4:**
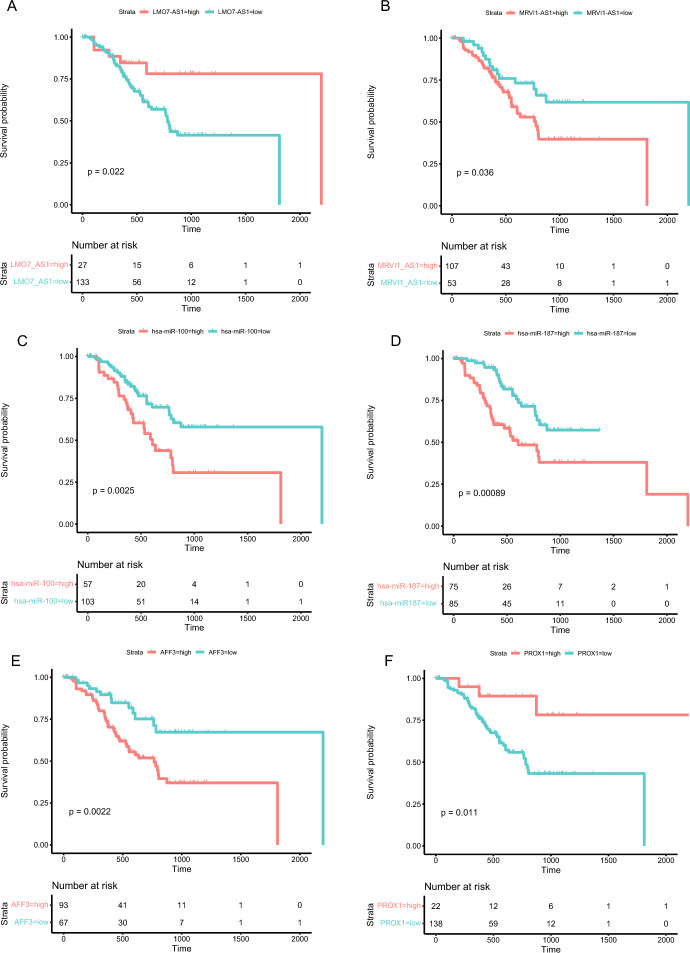
Kaplan–Meier survival analysis of selected lncRNA (A: *LMO7-AS1*; B: *MRVI1-AS1*), miRNA (C: *has-miR-100*; D: *has-miR-187*), and mRNA (E: *AFF3*; F: *PROX1*) in STAD. The x-axis represents the survival time, while the y-axis represents survival probability. The table counts the number of patients for each group

Kaplan–Meier survival analyses and log-rank tests for each gene were carried out to assess the contributions of gene expression levels to global OS time (Liu et al. 2022). Thirteen lncRNAs, 25 miRNAs, and nine mRNAs were put into this survival analysis model in order to identify the probable genes with substantial associations with the prognostic characteristics of patients with STAD. The cutoff value was set based on surv_cutpoint() function from survminer R package. There are 18 genes, including two lncRNAs, 12 miRNAs, and four mRNAs, which significantly differ between low- and high-expression levels groups (P value ≤ 0.05, Table S5). We selected two genes for each gene type (lncRNA, Fig. 4A, B: *LMO7-AS1* and *MRVI1-AS1*; miRNA, Fig. 4C, D: *hsa-miR-100* and *hsa-miR-187*; and mRNA, Fig. 4:E, F: *AFF3* and *PROX1*) to illustrate the difference in survival probability grouped by gene expression levels.

### Construction of Risk Score Model

LncRNAs, which act as the primary miRNA and mRNA effectors, predominate in the upstream area of the ceRNA network (Zhang et al. 2019b; Li et al. 2019a). Also, lncRNA has highly specific expression distribution patterns, making them the ideal biomarkers for identifying STAD and guiding its prognosis (Chan and Tay 2018; Han et al. 2021). Therefore, multivariate Cox regression analysis was used to determine possible prognostic-related lncRNAs based on the 13 lncRNAs in the ceRNA regulatory network, and their contributions were weighed by their relative coefficients. The risk score model was then updated to include *LINC00486* [*P*-value = 0.031 and *HR* = 1.002 (1.000–1.003)] and *LSAMP-AS1* [*P*-value = 0.047 and *HR* = 1.008 (1.000–1.015)] and the final risk score formula was as follows:

$$Risk score=\left(1.61\times {10}^{-3}\times the expression level of LINC00486\right)+\left(7.67\times {10}^{-3}\times the expression level of LSAMP-AS1\right).$$

**Fig. 5:**
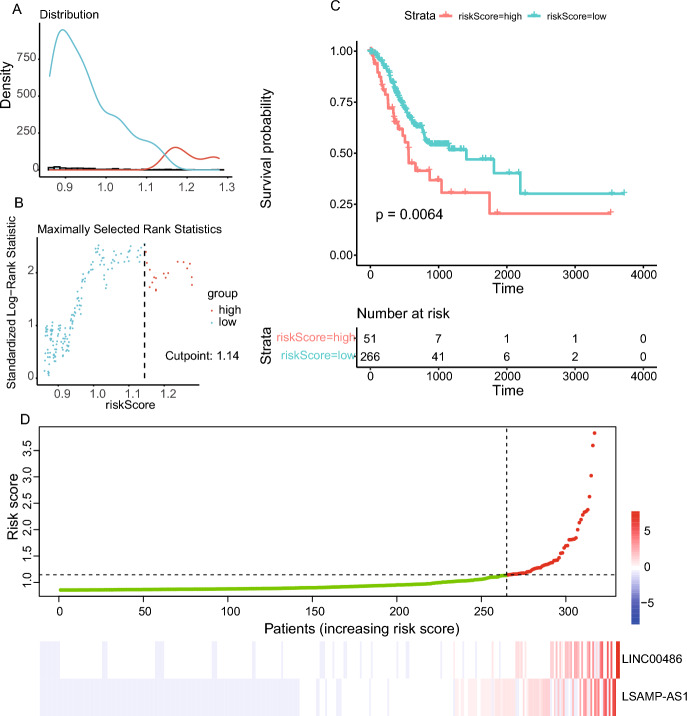
Risk score model. **A** The distribution of risk score values. **B** Maximally selected rank statistics for risk score. **C** Kaplan–Meier survival curve between low- and high-risk score groups. **D** The Construction of prognostic models of risk score. The x-axis is the patient sorted by risk. The y-axis is the risk score. The red dot indicates the risk score for high-risk patients, while the green shows the risk score for low-risk patients. LncRNAs’ expression levels in STAD patients

Positive coefficients were found for *LINC00486* and *LSAMP-AS1* in both the univariate and multivariate Cox regression analyses. This phenomenon suggested that they are cancer risk factors for the survival time in STAD. As shown in Fig. 5A, B, the distribution of all risk scores and maximally selected rank statistics were analyzed by surv_cutpoint() function in the survival R package. Thus, we set 1.14 as the optimal cutoff. Patients with risk scores less than or equal to the cutoff were assigned to the low-risk group (315 patients), and those with risk scores greater than the cutoff were categorized into the high-risk group (60 patients), respectively. The risk score signature’s Kaplan–Meier survival analysis revealed a significant difference in survival times between the low- and high-risk score groups (*P*-value ≤ 0.05, Fig. 5C). Scatter plot and heatmap showed the lncRNAs’ expression profiles and risk scores of 315 patients with survival time (Fig. 5D). The results revealed that patients’ risk scores increased as the expression levels of lncRNAs.

### Identification of Independent Prognostic Factors

**Fig. 6:**
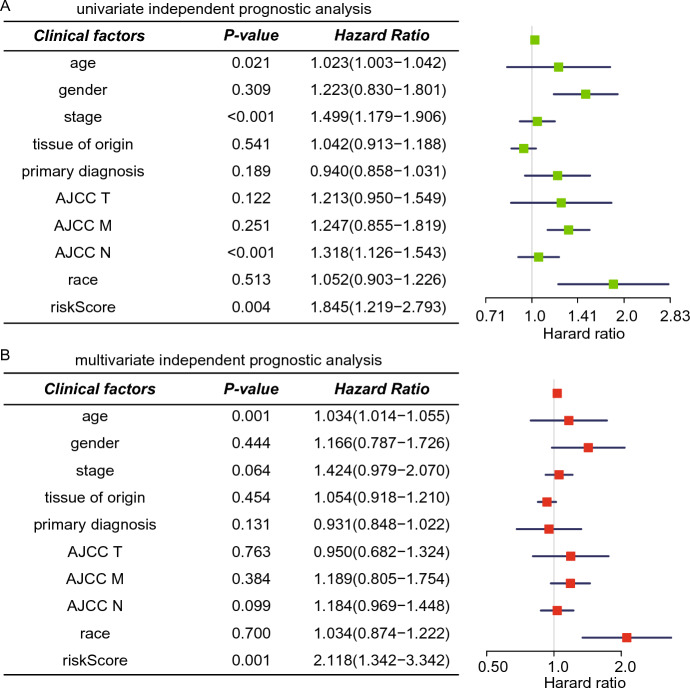
**A** Forest plot of independent univariate analysis in STAD. The green forest plot suggested that age, stage, AJCC N, and risk score are related to survival time and status. One of them can serve as an independent clinical characteristic (*P*-value ≤ 0.05). **B** Forest plot of independent multivariable prognostic analysis in STAD. The red forest plot suggested that age and risk score are related to survival time and status. The risk score can be an independent characteristic (*P*-value ≤ 0.05)

Following this, a univariate Cox regression analysis was carried out to examine the 317 patients with complete clinical data for any markers that might be associated with OS. Based on the following clinical traits, we separated the patients into different groups: age, gender, stage, tissue of origin, primary diagnosis, AJCC T, AJCC M, AJCC N, race, and risk score. The finding demonstrated that, like risk score, the age, stage, and AJCC N had statistically significant prognostic values in the green forest plot (Fig. 6A).

The red forest plot demonstrated that stage and AJCC N clinical factors were not linked to the prognosis of STAD patients in the multivariate Cox regression analysis. Thus, an independent predictive indicator of survival time for STAD patients was the risk score system created from the expression levels of lncRNAs in the ceRNA regulatory network (Fig. 6B).

### Relationships Between lncRNA, mRNA, and miRNA

**Fig. 7:**
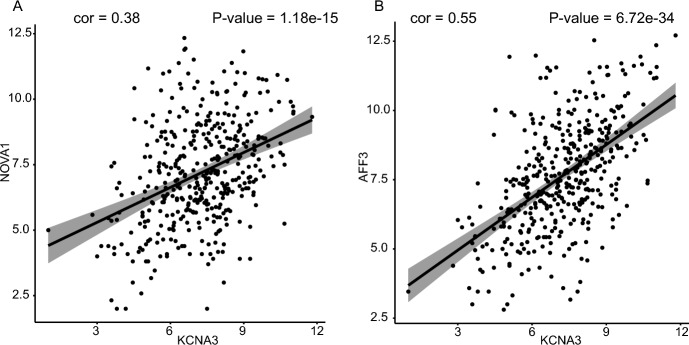
The correlation between lncRNA and mRNA. **A** lncRNA *KCNA3* and mRNA *NOVA1*. **B** lncRNA *KCNA3* and mRNA *AFF3*. The x-axis indicates the relative expression levels of lncRNA *KCNA3*, while the y-axis indicates the relative expression levels of mRNA *NOVA1* and *AFF3*. The gray area represents a 95% confidence interval

Under the ceRNA mechanism concept, lncRNAs interact directly with miRNAs to positively affect mRNA expression (Zhang et al. 2021). Regression analysis of 13 lncRNAs and nine mRNAs was performed to confirm this positive relationship phenomenon in STAD. The correlation values and *P*-values were calculated and counted. There are 21 positive correlation pairs, including eight lncRNAs and nine mRNAs (*P*-value ≤ 0.05 and cor > 0.3, Table S6). Furthermore, we explored to see if the lncRNAs and mRNAs shared any miRNAs. The findings demonstrated the importance of hsa-miR-217 as a critical miRNA in several ceRNA pathways, including *KCNA3-hsa-miR-217-AFF3* and *KCNA3-hsa-miR-217-NOVA1*. We plotted the linear regression curves between *KCNA3* and mRNAs in Fig. 7A, B.

Based on the theory basis of Barbagallo et al.’s research, there is a negative expression correlation between lncRNA and miRNA, there is a positive expression correlation between lncRNA and mRNA, and there is a negative expression correlation between miRNA and mRNA (Barbagallo et al. 2021). Therefore, we performed regression analysis for 13 lncRNAs and 25 miRNAs. The results showed that there are 34 positive correlation lncRNA-mRNA pairs, including 8 lncRNAs and 9 mRNAs (Table S6). We performed regression analysis for 13 lncRNAs and 25 miRNAs. The results showed that there are 34 negative correlation lncRNA-miRNA pairs, including 6 lncRNAs and 14 miRNAs (Table S7). We performed regression analysis for 9 mRNAs and 25 miRNAs. There are 36 negative correlation mRNA-miRNA pairs, including 7 mRNAs and 17 miRNAs (Table S8).

## Discussion

As the fourth most frequent cause of cancer-related deaths worldwide and the fifth most common malignancy, STAD continues to be a challenge for global health (Ilic and Ilic 2022). Patients with STAD continue to have a poor prognosis and an inadequate survival rate despite large improvements in diagnosis, prevention, and therapy (Ye et al. 2021). The ceRNA regulatory network is crucial to cancer development (Su et al. 2021). ceRNA network disruption affects cell growth and development, leading to various diseases, including cancer (Su et al. 2021). Unfortunately, very little research has been performed on the ceRNA regulatory network in STAD prognosis.

Interestingly, recent research has provided fresh insight into the role of lncRNAs in the development of STAD (Luo et al. 2021). In the tissues of STAD, a sizable number of miRNAs exhibit variable expression (Wu et al. 2010). Protein-coding proto-oncogenes and tumor-suppressor genes are altered genetically and epigenetically during the multi-step process of STAD (Huo et al. 2021). In particular, cytoplasmic lncRNAs are essential for a variety of molecular processes in both animal and human cells. They have the ability to influence mRNA stability, control mRNA translation, act as ceRNAs, act as miRNA precursors, and mediate protein changes (Rashid et al. 2016). Certain cytoplasm lncRNAs can control how cytoplasmic proteins are transported into the nucleus to activate transcription (Willingham et al. 2005). Thus, we focus on the lncRNA-miRNA-mRNA regulatory ceRNA network in this research. Also, we find an optimal cutoff point to make a difference in survival analysis. In which we only selected lncRNA, which is located in the cytoplasm. Based on survival analysis, *LMO7-AS1* and *MRVI1-AS1* were recognized as possible prognostic biomarkers and therapeutic targets in STAD. High expression of *LMO7-AS1* was linked to a poor prognosis in patients with childhood kidney cancer, according to research by Zheng et al. (Zheng et al. 2020a). *LMO7-AS1* was up-regulated in colorectal cancer (CRC) tumors compared to non-tumor tissues (Yang and Kang 2020). Qin et al. reported that *LMO7-AS1* was positively correlated with poor prognosis of CRC by screening 240 differentially expressed immune-associated lncRNAs between CRC tissues and normal tissues using the bioinformatics method (Qin et al. 2021). The survival analysis identified *has-miR-100* and *has-miR-187* as potential prognostic biomarkers in STAD. *miR-100* is the most important miRNA related to the progression of GC (Ueda et al. 2010). MD et al. suggested that by performing cluster analysis, *miR-100* is up-regulated in diffuse-type GC (Ueda et al. 2010). *miRNA-100* was also up-regulated in pancreatic adenocarcinoma (Bloomston et al. 2007). By blocking *FOXA2*, *miR-187* encourages the growth and spread of GC (Li et al. 2017). In GC, *miR-187* may serve as a biomarker and therapeutic target (Chen et al. 2018). *AFF3* and *PROX1* have been deemed to be candidate prognostic biomarkers in STAD on the basis of survival analysis. In a recent study, AF4/FMR2 family member 3 (*AFF3*) was proposed by Zeng et al. as a novel prognosis-related biomarker that can be used for immunotherapy by an integrated multi-omic framework (Zeng et al. 2022). *AFF3* has recently been discovered to play a significant role in the initiation and growth of several malignancies, including glioma, breast cancer, and adrenocortical carcinoma (Zeng et al. 2022).

Additionally, the Cox regression analysis got the lncRNAs *LINC00486* and *LSAMP-AS1* from the study of 13 lncRNAs. Ma et al. concluded a novel lncRNA *LSAMP-AS1* is involved in the prostate cancer process via targeting *miR-183-5p/DCN* axis, and they reported that *LSAMP-AS1* binds to *microRNA-183-5p* to suppress the progression of prostate cancer by up-regulating the tumor-suppressor DCN (Ma et al. 2020). The ceRNA regulatory network’s dysregulated mRNA function is mainly located in the following GO terms: transcription factor activity, nucleoplasm, chromatin, RNA polymerase II core promoter proximal region sequence-specific DNA binding, and positive regulation of cell proliferation. *PROX1*, *AFF3*, *SOX11*, *E2F3*, and *RUNX1* were involved in the transcription factor activity GO term. One of the most important controls over mammalian expression is runt-related transcription factor 1 (*RUNX1*) (Tuo et al. 2022). In cohorts of glioma, pancreatic cancer, colorectal cancer, cervical cancer, renal cancer, lung cancer, ovarian cancer, and gastric cancer, *RUNX1* is a worse predictive indicator (Lin 2022). On the other hand, better clinical outcomes are associated with patients with higher levels of *RUNX1* expression in both breast and prostate cancer patients (Lin 2022). By targeting *RUNX1* and activating the Hippo signaling pathway, *miR-28b-3p* prevents invasion, migration, and epithelial-mesenchymal transition in GC (Bao and Guo 2022). *SOX11*, *E2F3*, *EXH2*, and *PROX1* were involved in the positive regulation of cell proliferation GO term. Due to its methyltransferase activity, the Polycomb group protein enhancer of zeste homolog 2 (*EZH2*), a member of the PRCs family, can play functional roles in the cell. By stimulating H3K27me3, *EZH2* influences gene expression (Mirzaei et al. 2022). *EZH2* inhibitors have reportedly reduced metastasis and neovascularization in human malignancies. *EZH2* inhibitors have been used to treat colon cancer because they cause tumor cells to respond to them more frequently (stage II and III) (Mirzaei et al. 2022).

In particular, we have identified two promising ceRNA networks: *KCNA3-has-miR-217-NOVA1* and *KCNA3-has-miR-217-AFF3*, involved in STAD progression and prognosis. Also known to be dysregulated in PCa are the following K+ channels, which have been suggested as biomarkers. In high-grade malignancies, Potassium Voltage-Gated Channel Subfamily A Member 3 (*KCNA3*) is down-regulated and mostly expressed in the early stages of cancer growth (Abdul and Hoosein 2006). LncRNA *KCNA3* serves as a potential prognostic biomarker and therapeutic target for CRC since it inhibits the growth of tumors by downregulating the expression of *YAP1* (Zhong et al. 2018). When compared to the normal stomach gland tissues, *KCNA3* has been found and be significantly down-regulated in stage-I STADs, which suggests it is very useful as an early diagnostic indicator (Tan et al. 2021). In cancer, *miR-217* is typically dysregulated. Chen et al. demonstrated that tumor tissue has lower levels of *miR-217* than the nearby normal tissue (Dente-Cassidy 1989). In patients with GC, a low expression level of *miR-217* was linked to aggressive tumor characteristics and poor OS (Dente-Cassidy 1989). The expression of neuro-oncological ventral antigen 1 (NOVA1) was suppressed in GC, and ectopic *NOVA1* expression in tumor cells contributes to tumor growth and a poor prognosis (Kim et al. 2017). Reduced *NOVA1*, which was suppressed by *miR-146b-5p*, is a possible biomarker for predicting poor prognosis in individuals with GC (Yoon et al. 2016). It is also a biomarker of concealed residual disease in leftover tissues following GC resection (Yoon et al. 2016). Furthermore, Shen et al. showed that downregulating *NOVA1* by *miR-339* overexpression in GC cells inhibits malignant characteristics like proliferation, invasion, migration, and oncogenicity while restoring *NOVA1* in *miR-339*-overexpressing GC cells partially reverses the inhibitory effects of *miR-339* (Shen et al. 2015).

However, our study has some limitations. External validation was not done because there are not any other STAD-associated lncRNA, miRNA, and mRNA databases that are comparable. Furthermore, some exploratory experiments are still required to assess the roles of unreported regulatory elements.

## Conclusions

To summarize, we described a method for building a lncRNA-mRNA-ceRNA regulatory network based on the connections between genes and lncRNA located in the cytoplasm. This strategy offers a comprehensive analysis network in addition to limiting the study area and improving prediction accuracy for the target lncRNAs that have a great deal of potential to be used as candidate biomarkers for the diagnosis, prognosis, and treatment targets of STAD patients.

## Supplementary Information

Below is the link to the electronic supplementary material.

Supplementary file1 (DOCX 287 KB)

## Data Availability

The public availability dataset of this research is from the accessibility database. The lncRNA, mRNA, miRNA expression profiles, and clinical data were downloaded from the TCGA Research Network: https://www.cancer.gov/tcga.

## Abbreviations

TCGA: The cancer genome atlas
STAD: Stomach adenocarcinoma
GC: Gastric cancer
LncRNA: Long NonCoding RNA
miRNA: MicroRNA
ceRNA: Competing endogenous RNA
GO: Gene ontology
FDR: False discovery rate
KEGG: Kyoto encyclopedia of genes and genomes
DEG: Differentially expressed gene
DAVID: The database for annotation, visualization and integrated discovery
AJCC: The American joint committee on cancer
CRC: Colorectal cancer
OS: Overall survival

## Code Availability

All code for data cleaning and analysis associated with the current submission is available at https://github.com/liuzhe93/ceRNA-Projects.
