# Supplementary material for: Uncovering the ceRNA Network Related to the Prognosis of Stomach Adenocarcinoma Among 898 Patient Samples
Source: Biochem Genet. 2024 Feb 15;62(6):4770–90. doi: 10.1007/s10528-023-10656-7 (PMC11604743; doi:10.1007/s10528-023-10656-7)
Supplement: Supplementary file 1 — Supplementary file1 (DOCX 287 KB) [file 10528_2023_10656_MOESM1_ESM.docx]

**Supplementary Table 1. The interactions between lncRNAs and miRNAs.**

| LncRNA_GeneSymbol | LncRNA_GeneID | LncRNA_Log2FC | LncRNA_FDR | miRNA |
| --- | --- | --- | --- | --- |
| ABCA9-AS1 | ENSG00000231749 | 1.55483145 | 0.000631926 | hsa-miR-195 |
| ADARB2-AS1 | ENSG00000205696 | 1.800239814 | 0.032457996 | hsa-miR-503, hsa-miR-195, hsa-miR-205 |
| ATP1B3-AS1 | ENSG00000244124 | -1.49976218 | 1.20E-14 | hsa-miR-93, hsa-miR-96, hsa-miR-122, hsa-miR-182, hsa-miR-204, hsa-miR-372 |
| C14orf132 | ENSG00000227051 | -1.73307131 | 5.91E-13 | hsa-miR-122, hsa-miR-141, hsa-miR-17, hsa-miR-182, hsa-miR-195, hsa-miR-200a, hsa-miR-204, hsa-miR-216a, hsa-miR-222, hsa-miR-372, hsa-miR-503, hsa-miR-508, hsa-miR-93, hsa-miR-96 |
| C6orf123 | ENSG00000146521 | 1.621820267 | 9.36E-06 | hsa-miR-204, hsa-miR-184, hsa-miR-222, hsa-miR-217, hsa-miR-143 |
| DIAPH3-AS1 | ENSG00000227528 | 1.158102506 | 0.006578142 | hsa-miR-216a, hsa-miR-182, hsa-miR-96, hsa-miR-210 |
| ERVMER61-1 | ENSG00000230426 | 2.741382492 | 2.24E-06 | hsa-miR-182, hsa-miR-205, hsa-miR-96, hsa-miR-204, hsa-miR-21 |
| H19 | ENSG00000130600 | 2.34820373 | 5.71E-05 | hsa-miR-301b, hsa-miR-141, hsa-miR-200a, hsa-miR-17, hsa-miR-93 |
| IGF2-AS | ENSG00000099869 | 2.457677063 | 0.000360948 | hsa-miR-122, hsa-miR-17, hsa-miR-93, hsa-miR-503 |
| KCNA3 | ENSG00000177272 | -1.423146373 | 1.14E-08 | hsa-miR-122, hsa-miR-141, hsa-miR-143, hsa-miR-183, hsa-miR-195, hsa-miR-200a, hsa-miR-204, hsa-miR-21, hsa-miR-210, hsa-miR-217, hsa-miR-50 |
| KCNQ1OT1 | ENSG00000258492 | 1.212816358 | 2.21E-06 | hsa-miR-122, hsa-miR-141, hsa-miR-143, hsa-miR-145, hsa-miR-17, hsa-miR-182, hsa-miR-183, hsa-miR-187, hsa-miR-195, hsa-miR-200a, hsa-miR-204, hsa-miR-205, hsa-miR-216a, hsa-miR-217, hsa-miR-222, hsa-miR-223, hsa-miR-301b, hsa-miR-363, hsa-miR-372, hsa-miR-503, hsa-miR-508, hsa-miR-551a, hsa-miR-93, hsa-miR-96 |
| LINC00086 | ENSG00000178947 | -1.628713073 | 3.54E-11 | hsa-miR-195, hsa-miR-301b, hsa-miR-182, hsa-miR-205, hsa-miR-187, hsa-miR-503 |
| LINC00087 | ENSG00000196972 | -1.645416341 | 2.44E-15 | hsa-miR-93, hsa-miR-205, hsa-miR-17, hsa-miR-503, hsa-miR-182 |
| LINC00114 | ENSG00000223806 | 2.26822831 | 0.000469964 | hsa-miR-143, hsa-miR-182, hsa-miR-204, hsa-miR-216a, hsa-miR-363, hsa-miR-508, hsa-miR-96 |
| LINC00462 | ENSG00000233610 | 1.189885453 | 0.03436265 | hsa-miR-363, hsa-miR-17, hsa-miR-372, hsa-miR-93 |
| LINC00486 | ENSG00000230876 | 1.858050872 | 0.007428603 | hsa-miR-122, hsa-miR-145, hsa-miR-182, hsa-miR-187, hsa-miR-204, hsa-miR-222, hsa-miR-96 |
| LINC00501 | ENSG00000203645 | 1.914717814 | 6.14E-06 | hsa-miR-183, hsa-miR-204, hsa-miR-301b, hsa-miR-363 |
| LMO7-AS1 | ENSG00000228444 | 1.132539273 | 9.30E-08 | hsa-miR-122, hsa-miR-145, hsa-miR-17, hsa-miR-204, hsa-miR-372, hsa-miR-93 |
| LSAMP-AS1 | ENSG00000240922 | 1.774708689 | 0.000177377 | hsa-miR-183 |
| MIR205HG | ENSG00000230937 | -3.363174644 | 1.61E-07 | hsa-miR-122, hsa-miR-143, hsa-miR-145, hsa-miR-183, hsa-miR-204, hsa-miR-205, hsa-miR-222, hsa-miR-301b |
| MRVI1-AS1 | ENSG00000177112 | -1.223696352 | 1.66E-07 | hsa-miR-183, hsa-miR-205, hsa-miR-222 |
| PART1 | ENSG00000152931 | -2.541641297 | 3.68E-17 | hsa-miR-100, hsa-miR-122, hsa-miR-141, hsa-miR-143, hsa-miR-145, hsa-miR-187, hsa-miR-195, hsa-miR-200a, hsa-miR-204, hsa-miR-205, hsa-miR-21, hsa-miR-301b, hsa-miR-363, hsa-miR-508 |
| RBMS3-AS3 | ENSG00000235904 | -2.238783704 | 1.08E-22 | hsa-miR-204, hsa-miR-96, hsa-miR-182 |
| SHANK2-AS1 | ENSG00000226627 | 1.328287525 | 3.01E-05 | hsa-miR-141, hsa-miR-143, hsa-miR-145, hsa-miR-17, hsa-miR-200a, hsa-miR-372, hsa-miR-93 |
| SOX21-AS1 | ENSG00000227640 | -2.5863628 | 1.81E-11 | hsa-miR-301b, hsa-miR-503 |
| UCA1 | ENSG00000214049 | 2.730931687 | 1.27E-05 | hsa-miR-122, hsa-miR-143, hsa-miR-182, hsa-miR-184, hsa-miR-96 |
| LncRNA_GeneSymbol | LncRNA_GeneID | LncRNA_Log2FC | LncRNA_FDR | miRNA |
| ABCA9-AS1 | ENSG00000231749 | 1.55483145 | 0.000631926 | hsa-miR-195 |

**Supplementary Table 2. The interactions between miRNA and mRNAs.**

| miRNA_GeneSymbol | miRNA_GeneID | miRNA_Log2FC | miRNA_FDR | mRNA |
| --- | --- | --- | --- | --- |
| hsa-miR-217 | ENSG00000207548 | 1.72E+00 | 1.32E-07 | AFF3, ANLN, E2F3, EZH2, FRMD5, NOVA1, PROX1, RUNX1, SOX11 |
| hsa-miR-184 | ENSG00000207695 | 3.71E+00 | 7.57E-08 | CRISPLD2, FSCN1, HAND2, SIDT2, USH1G, ZFPM2 |
| hsa-miR-551a | ENSG00000207776 | 2.00E+00 | 3.89E-07 | GALNTL6 |

**Supplementary Table 3. Intracellular localization of lncRNAs.**

| LncRNA_GeneSymbol | Location | Sequence |
| --- | --- | --- |
| ABCA9-AS1 | Nucleus | GCTGCCGCCTTGCAGTTTGATCTCAGACTGCTGTGCTAGCAATCAGCGAGACTCCGTGGGCGTAGGACCCTCTGAGCCAGTCACCAATGCTGCTCATTGCAATGTATGGAGTGAAAGAGGCTGCCATCGGTATCCAGAAGAAGGTGTTACTTCGGTACCCATACTCATAATCCATATGCTCCTGAAATCATGTGGAACATGCAACTAGGACCTTCTGAACACGTAGCCTGATTTACTTATGAAGAAGCAAATTCAACCAAGTGATACGAATTGTCTAAGGTCATACAGTTAATAACTTGTAAGAGCCCAGCACAGCACAGATTTACAAAATGATACACAAAGACCTCAGAGGTGAGTCAATGATTTACCTCCCTACCTCCTCTTTAGTTGCTTTGTGCAACAAACATTAAGGTTAAGTAAACTTGCAGAATCTATTTCTAAAACAAACTCTCCCATGTCGTATTTTTCATTTTTTAATTTATTTTCTGTTTGGCCTCATATATGCTTTCTAAAACATACAGAG |
| ADARB2-AS1 | Cytosol | GCCGGATGACATGAAGCAGCTGTTCCCGCCTCCTCCTGGCACCTCCCTGACTCACGCACTTGGTGCGTGGAGGGGTCGTGAGCGGGCACAGGCAGCCACTTCGCTGCTTGCCTCATCAGCCTCACAGTTCCCCACAGATGATCCCTGCATTAGTTGGCGGAGCTGATGGTGTTCCCTGTTTTACCCTGTGGTCTCATTTCCGGATGCTGCTACCAATGCCCGTCTCTATGGCTCCCTGCCCACGTGTGCAGCCCCGTCACTGAGAGGGAAGTGGCTGGAGAGGTGTCCAGATCATTGTGAATGAAGTCATCTGTGAAACCATGGGAGAGAGATTTGATATCTGCTTCCCTTTCAAATGAAAGGAAAAGAGTGGGGTGAGAGAGGGCGGCACTCCCCAGACAGGCCCTGACCACACCCTGGCCCCACGCCTCAGCAGAACAACTCTGAACCTCTATGGACTCACAGGGGCGAGGTCGGGGCAAGGAGACTCAGGGCCTGCGCTGCTCACCGGGTGAGAAGTCACAGCTGGTTAGGAGGCCACAGACGGCAATGTCGCTGATGGCGAGATTCCGGGCCATCCATTTATCCTGTGTGGAGGCTGACAGGGACGCATGCCTCATCTGCT |
| ATP1B3-AS1 | Nucleus | TGACTAGCCTAGGGAATAAATGCCAAATTTTACAAACATTTCTTCCACTTTTGTGCTTTTCTAAAAACAAACAAAAAATCCACAACTAAATAAACAAAATTAAAACTCCACCTGCTTTGATTATTTTATGTACTTCACACACCTCTCTCCAAAGCAAAAGGGAAAGCTAACTGAAATCCGAATCATTAAATCTGGGTTTTTCATTAACATTTCCCCCAACTATTCTAGAAGCAAAGGATACGTAATAGATAAACAAGAAGAAAAATGCATTTAAAACCTTAAGGGGACCCAGCACTCCATCCAAGCTTATCTATGCAGCCTGCTCACAGGCTCCTGTATACAGGCGCACTGTCATGAAATTAGCACTTCTACCCAG |
| C14orf132 | Exosome | GCGCCGTGCGGTCTCCGGACGCTCGCTGCTCAGCCCGATCCCCGCCAACTGTGCAGGCGGCTGACCCGCAGCGGCAGCGGCAGCAGCGAGGACTCGAGCGCTGGCTGCAGCGACACCATGGATCTCTCCTTTATGGCCGCGCAGAACAAAGACAAATCCCGGATTTCTCCATCAGTCTGTGACCCTAGAGAAGACCCAGAGCTGGCTCCAGGGAAGGGCTGCGTTTGGCCTGGGAGACTGCCCATGATGGGGGGAGCTTTCATGGACTCGCCCAACGAGGACTTCAGCACCGAGTACTCCCTGTTTAACTCCTCTGCCAATGTCCACGCGGCTGCCAATGGCCAGGGCCAGCCGGAAGATCCTCCTCGGTCCTCCAACGACGCCGTCTTGCTATGGATTGCCATCATAGCTACGCTGGGGAACATCGTGGTGGTGGGCGTGGTGTATGCCTTCACCTTCTGAGGACGGCACACCCTGCACCACCATGGGGTGAGGCTTGGCACGTAGCTCTGACTTGCTGTCGGCCTTTGGCTTCT |
| C6orf123 | Nucleus | AGGACACGTGGAAACGCAACCGCCAGGAGGAAAGGCGGGACACAGCGGGAAGGGAGGTTTGCCTCCCCTCTCTTCCTGTGGTGACCCCCACCTCACTTACAAAAATCACCACCGACGTTTGGGAACCGTGCACGCCGACTGAAAGCAAAGCTCAGCTGACCTGCGAGGAGTCCCCGGGGAGCTGCCGCGGTGGTCGCCGTTGAGTCCGTCCGTGCCGCTGGGGCCGAGGCAGCTGGAGATGTGGTCAACGGGCTGATGGGCACGGCTGTGGGACCCCATCATTCCCCTGCCCCGCATGACTCTGCTCTCCCAGCTCGACTGCTCACTTCAGACTTCCCTTATGGAAGGAGTTGCCAGATAGAGCAAGTAAAATACAGTGTGCCAGACACAGGTTTATTTCAACACTGGGAAGGCTCCATTCCTACTTAAACCCAGCAGGGAACAGGGAGCGGTGTGCAGAGGAGGTGGCCTGCCCGGGAAGCCGGCCTTGGTGCTGGGACTGCAGGGAGAGGGCTGTGAGGAAGAGCACAGGCTTCATGCACATTGCACAGAGGAGGAGATTCATGGGGACAGCAGGCACTGGTTAGGCACAGCGGGAAGGTTATCGAAGACAAAACACATTTCTACCCAAGTTTAAATGACCTGCTATGCCCACAGAGCTTGATCCAGAAGTGCACAGGTAAGTATTTAGCAGGCTGGCCGATGTTGATTTGAACAATCTCAAGAATGTGCTCTTTAGTGCATTAAACATATGTGACGAGAGCTGAGCACGTTTAAAATCTCAGTAACTTTGGGGTTTGTAAGTGTTGTAGCTCATAGTGTCGGTTAAGGTTAGACATCTCAGTCACATGCTGTCTTGGCACCAGCTGTGGACTGTGCTGTGAAAGAGATCTGTGACCTTGCACCCTGAGATCTCAGGCTCAATTAAGAGACAAAACAGGCAATGCAGTATCAGTGCAGATGGGAAAGAGAAAGCAGAAAGTATGAAGAGGGAAAGGAGGAGCACAAAGGAGTTAGCATTAGTCTCTACTGAGCAATTCCTATTCCAACCACGTCCTAAATGATGAGTTGGATCCTAGCAGAAAGGGAACACATGTTATTTTTAACTGATTTTAACTTGATTTATTGTAACCTATCAGGAGCTCTTAATAAATATGGCTCTTTATGTTGTTA |
| DIAPH3-AS1 | Cytoplasm | CTCGTGTAGGACGCCCAACATCTGGACGCACACAGCATGACTACTAGTGCTGAGCAGCCACCCACAATGAATTACCTCACAGGATTGCTGGTCAAAGACACTCGGAGAGACTCCAGGCATGTGACAAGTCTCTCATCTGCAGACCCCATTTTCAGCTCATGAATGAATTCCTGAGATTCACCATCCAACGGCAGGAAATGAATTGTGTAAGTTCAAAAGAAGATCAGGTGAAGTGGTACGAAATTCGGTGTTTTTCATGTTTTTTGTTTTTATTAATTTACTATAATGTTATTCAAAACAACATTCCCAATAAAGCAAAACAAATTAGCACTGTAAA |
| ERVMER61-1 | Cytoplasm | AGGACGGATCAGCAGCTCCCTGCCACTGTCCCCTCCCTGCCAGGGCTGGGACACATGGCTCAAGGCGCTGCAGAAGGCTGGCTGGCGTTTTCTACCATGTGCCCATGGAATCTCCTCCCACAGTCAGGGAGCCCAGCTCCAACCCACAGCAATTACACTTCTCCCCCTGGTGAAGGAAACACTTGCATAAGAATAAGAGGTTCTTCCACAGGCATTTTAAAACCTTTTTTCTTTCCTCTTCTCCACAATATCAGCATTTAAGTTTAAGCAAGTTTTTTTATTTCTAGAAGACATTTTACTAGGCAAGGAATGATAAGAATCCCTGTGTATATTCTCTATTAAGCTTTAATTGTGAAAAAGGATTTGTAGGGCTAGTCTTGGGCTGTGGCCAATCTGGTATGCTTCCTGTGTCTGTATGGTTTGTGCTGTAAGCCTCCATCTTGTTTTACACATCCTGGGGACATGGCCCACAACTGCTTGACAGGACTTTGTTTAGCAGTCCTGCCTTAGGGGATCAGCCCTCTCTGGCCAATATCTGCATGTTTTCCTACTCCTGTCTCTTAAAGGGCCCCACCCAGCGACTGGATTTTCTTCTGCCTCTCTGTGTGGGTACTGTGTGTGATATCTGTAAAAAGTGCGCTAATTAATTTGGCCTAAAGAAAGACAAGAACTTGGATCAAATTTTTTTTAAGGGAAGTTAAAAGCTGTGGTACCTTTCAGTTCACATGACTTTAATCTCTGAGAAATAAAAACAGCCCTAAAGACTATTGGTAAAATGCAGGTGAGATGCAAGGTTTTCTAAGTGTTTTGAGGTTAAAAACTGCTTTTTGGGTTTTGAGAACTATTTGACTTGAAGGCTTCACAATTGGTAAGGCCTGGGGACATATGGAAATAACCACGCTCTTAATTATGCTGGGAGTCAAACCTTGGCTGCACCTAGCACACAATTAAACAACTTACCAAGTTTTTACCTTAAAAGTTAAAAATTGCTAGGAGTTACTATTCCGAGATGTAATTGAGACTACAGGAAATAGATTTATATGCAAGATGTGTAAGAACAGTAAAATGTGGTGTTTTTTTGTAAAATATTATAAGAAGGCATGGAAATGTATACTTTTGCTTAGGGTTAAAGGATTGTTTAAATTAGGAAAAAGCTGAAGGTTCAAACAAGTGGTGGAGAATTGTGGAAATTAATCTTGCAGAAGAGGTTCAACATATTAACTAAATTCAAAAGGGTTATAAGGTTATAAAAGGTTTTTGCTTCTTTGAAATTTCTGAGTCATCCTTTTGGCAAAATAAATAACTTAATGGCAATTTGGAACTCTGTTTCATAATATCAAGTGTTTTAAACCTCAAACATGTTTGACAGCCTTCCCAAAATCAAACTTTGGTTTCAAAACTGTCTTCCCTGGCACTTGGCTTTTGGAATACTTCAGAGGGCCCCTGAAGTGTCCAGAAAAGAGAGGTAAACAGGGTTATTTGACATGTTTAGGTACATGGATTGCCAAAATGATGTTCAATCTTCTTTAGGTTATATCTTGGTAAATAATGCTAATATATGTTCCAAAATTGTATGGGACTTCTAAAATTCTAATGTCTAAGTATTTGCTACCAATCGTAATTAAGGTTGTTATGTTAAGTTATTGTAAACCACGGAGACAACCAAACTTCTTTTTCCATCGTGTTTCTAACTGTAAGTACCTTGGACATTTTTCTATTCACAGAAAATTGTCTTGTTTTAATCCTTTTCAAAAGATGGTTTATAATAAGCTATATGACTTTAACAGGCACTCTCAAATATAGGCTTCTGGTAACTTTGGAGATGGTAACATTGGAATAGAAGAAAATGTACAGGACACATAAAGAGCTGAAATGCTCAAGAATATCAAGCAAAACAAGAGTTAACTAAATGGACTGAACTCAGAAAGCTGAAGCAAACTTTTTGACTTTTGCTTGGAATATTGCTGATACTTGTTTTGTTTTTCAGAGTCAAGGAAATTATTTTGAACTGTTTACAGGAACGATAAACCAAAAGTGTTTTATACATTTTTGCTCTTTGAATTACTTCATTATTAACTTATCTGCAAAATTCCCTGAGAATATTTTAACATTATAGAGAAGCACTGTAAAACCTTGATAAATTACCCTTAAATATTTACCTAAATTAGATAGGAGCAGATAAGAAATATCTCTACCATCAACTTTCAGATATAACCAAATAAATGAACATATTC |
| H19 | Nucleus | ACAACCAACACGTTCTCCCCACACGACTCTCTCGTTCTCCCCACAGCCAGGTCTCCAGCTGGGGTGGACGTGCCCACCAGCTGCCGAAGGCCAAGACGCCAGGTCCGGTGGACGTGACAAGCAGGACATGACATGGTCCGGTGTGACGGCGAGGACAGAGGAGGCGCGTCCGGCCTTCCTGAACACCTTAGGCTGGTGGGGCTGCGGCAAGAAGCGGGTCTGTTTCTTTACTTCCTCCACGGAGTCGGCACACTATGGCTGCCCTCTGGGCTCCCAGAACCCACAACATGAAAGAAATGGTGCTACCCAGCTCAAGCCTGGGCCTTTGAATCCGGACACAAAACCCTCTAGCTTGGAAATGAATATGCTGCACTTTACAACCACTGCACTACCTGACTCAGGAATCGGCTCTGGAAGGTGAAGCTAGAGGAACCAGACCTCATCAGCCCAACATCAAAGACACCATCGGAACAGCAGCGCCCGCAGCACCCACCCCGCACCGGCGACTCCATCTTCATGGCCAC |
| IGF2-AS | Cytosol | ATGAGCAAACGAAAGTGGCGCGGATTTCGGGGCGCCCAGCAGGAGCGAGCGCAGCCGCCAGCCGCAAGTCCGCAGCCGTGTCCCGCGCCCCACGCCGGCCTCCCCGGCGGCAGCCGGAGACGAGCGCCAGCACCGGCGGGACAGCAGCAGATGCGGGCCGAGAGCCGGTCAGGGGCGCAGAGGCGGAGGGGATCGGCGCGGCGGGGAGCTCACCGCGAAGCTGGAGGCTGCGTCCGCGGGCGCACCAGGAGCTCAGGCAGCGAGCGATCGAACGCTCTGTGGCAGGCGGTGGACGCTGCTGAAGCTCTGGCTCTGAGCTCACCGCTCCGCCGTCCGTGGGACCAAGCCCAGCATTTTACAAACCCAGCTCCTTTCTCCAAGGGTCCTCAGAGCGCCCCTCCGTCACCCCCTGCAGGGCGCCGCAGACGAGGCGCTGACCTTGCGCTCACTCCCCTCGCTGGGGAAGGACACACTCGCTGGCGTCAGCCCGGCCGGCCTGGGAAGTAGGACTAAGGACCCGAACTGCAACCCTCCACACCAGACAGCACAGACCACCCCAAGGCTGCTCAATCTGCCCAAAGCCAAAAGTACGTGGGGGATATGGGTCCAGGTTGGGGTGCTTGGGAGAGGGCCCTGGGAGAGGGGTTCAGACGGTGCACACTCCAAAGGACTGAGGCGGCGCCACTGTGTTACCATTTTATTGAGTGTCTAAGGTAAAGACACCTTTGAAAGAACACTCCCGGCTTCTATCTGGGATGGGCAAATCAGCCTGAAGAGTCACCTGCTGGATCCCTCCCTGCAGGCCCTGTCGTGGGGCAGGTACAGGGACTCCAATCCATGTGCCTCCAGCTCCAACGCTCCCCAAGTCCTGGAGCCTCCCCTGTCACCCCAGTGCCTGACCAGTGAATGGGTGCTGCCCCCACTACAGAGCTCCCTCTGGCTGAGGCAGAGGCCACTGGCTGGGCCTTTCTAGCCACCTTTCACCCCTCTTGCACACGGGAAGCCCCGCAGGCTCCTGACCACAATGGGATCCTGACGCCGGAGCCCCTTAGGCCCTTTGCTCAGAAGGCCTCTGGTTATTACCTCAATTCTGAACAGCCAGGAGGCCTGAGACTGTCACCCACTTTGATAAATACAGACCGACAGAAAGGTCAGGCCATTGGTGCAAGACCCCATGGCCAGCAGCCTGGGGCGGGGAGAACCCGGGGGTCTCACAGCCCCCAGCAGAGCCCAGCTTTCCTTGGGTTTTTAGGTTCTTCCCCAATGATATTTTCCCTTTTTCCCTCTCTACCCACTTTCTGCCCCCCATCTTCTCCCCTTTGGCTGCCCGCTCTCTCCCTGCCCCTCTCCCCTTCTTTGCCCTCTTTCGTCTCCCAGGAGCTCCCCTGCCAGGACCTCGGTGCCGTGGAGACTGCGGGGAACCTGCTCTGCCCACACCTGCTCTGGCCCCTAGACGGAAGCACCTGGAGCCAACCACGGAACTCCAGATCCCAGCTGTGTGACTGAATCCACGCCAGCCTCTCTGAGCCAAAGCTGCTTGCAGAAGGGGGAGATCCCAGTTCGAAGACTCCCGCGCAGAGCCTGTGGCCCTCTCTGCCAGGCCTCAGGGTGCCTGAGACACTCACCTCTCTGCCTCGCAGTTGGGGCTGAGGCTGGGGCTGGCTGCCAGCCTCAGTTCTGGGAGCGCTGGGGTCGCCTGGGCCACAGGCCACAGCAGCTCACCTCAGGACTGGGCTCTCTGGCCTGCTGGGGCTCAGGCTGTGGGGCAGGCTGGGCAGGGGGCTGAGCTGGCAGCGATTCAGAGCCCTGGGGCTGGGGGCTGGAATCCACCTCCTCCCACACAAGCTCGGTGGTGACTCTTCGGCCC |
| KCNA3 | Cytoplasm | CTTAAGTACCCAGATTTTCTTATGCAGGAAAGAAACATGAAGTAAGATTTAAGCCTTGAAAGTATCATAAATATATCATTCTTCAAATTGATTGTGATGAAAGGAAATAGGATTCTTTCTCCTTAGAATTTTCACTGATCTATAATGGGATCAAAAAAGTAGAAAAATCAGTCTGCAGAATGAAAACAGATTAATGAAATAGCTAGAAAGGAGGGGAGAGCATTTGTTTAGTTAAATGTTAAGGTAACTTGAAAATTGTCTATGAAATGCTAAAACTGTACCAGAGCCTTATTGCACACTCAACATTTTAAGCCTGGGAGCAGGGGAAGGTGGGAAAAGGTTGGAGTGATAGAAGTGTTTGCCTTATGATCTCTTGACTCCTTTTACACAAGAAGCAGATTGGTAGAGCTTAAGCAGGTGTCTGTGCTGTGGTGCTGGCTCTGCCAAAAACTCTCATATGCCCTAGGAAAGTTCCTTTAACTTCTCTGAGCCCGAGTTTATCTGTAAAGTGAAGACTTTTTAAATTTAGTAATCTGTATGTCTCATCTCTGAGAATTCTATAATGTTTTCACATTGTTATATGTGTATATGTACATGTTGCTCCCCTCTAGTCACTAAGATGCCACATATTTCTACTTGAGTGAACAGATTGCAAGTCCAAATGTTCTTGTTTCTAATACTAATAATTGATCTCAGCAAGACATAAAAAAGTAAAAAAAAAAGTAATCTTACCTTTTATTTTGACCTTGGCTTTAAATTATTATGATTTATCATTTCATCTTTGAATGCTGAGTGAATTACCTAAACAGAATGTCTGTTCCAGTTAATCTTCTCTTTAAATTTTTGTAGTTTAATAAACATGTAACAAATCAGAAGCAAGTATATCATGCTTATCGATGAACTGTAGCAGATTGTAATGGTGCATTTTTGTCACATTAAAAAGTTAATGTCCTTTTGGTCCCAGAAGCAATTACTATAATTTAAAATAATATTCACAAAATTTTGTGAATGTTTTTGAATACAAAGGAGGCCTAATACAGCCATATTGTAGTTCATTTTGCTCAGGAGTGAGAAACAAAATTCAGGGATAACCCAAACATGTCTGTTTGGCAATAGTGGATAGTTAGGTAAAGAAGGGTTATAGCTTTTGCACATCACAGATAAGAGGCGACTATACCTATTGAAAAACAGCAGTATATTCTGGTTCTTGGTTTTATGATGGAAAGATATTAGAATGATACTAAAATTTGGATGCTTGGGAAATTGAATAATACAGAAATATTTTGTCATACTGAGATTATTTGTCCAGAAGTTTAGTCTTATATCCCTTCCATTCAGAAAAGGGGGCAGTTTTTAAAGGCCTCTGTCATAAAAGGAAATGTATATTTTGACTAGTGGAGCAATTAGTTAAAACATTTAATCTGATCATTTTGGTTTTATGAATCTGAATTTTCACTTTAGGTGTAATTGAGTCGCTTATGTATTACTGTGATCTTTGTGAGAAGTGATTTCGTCCATTTTTTTAAATCTGATGAAACTTGATAAATGGTGATTCTAGCATATTGTGTTAATACTGTATCTTACAACAATTTGTGAACTGACCATCATAAACGAATCATAGTAGTATCTCTTCTGGTAAGTTGTTTCTATATGTGATCTCTTAATGCTGGGTTTGGGGGTGGGGATGGGGTTAAGTATTTAGTTTACTTTAAACTGTCAAAGTGTTTTTCTTAAATGCCATAAAATTGTATGACTATAAGCCAAATAAGAATATTCTCTTTTTTCACTTTTAAGGGCACTGTATAGACCATTTTTGAAACTATCCCACAGAATGGTATAATTTCAGTTTAAAAGCTCTTACACACATACACACACACAAATATAAATATATATATATACACATTCAAGGGAAAGCAAACAGTGTATGTAGTATTTCTAAGTGACATGCTACACGTTATAAGAATCTATTTTTGAAATCTTATACAGATTATGCAGAAGTCAGAATAACAAATACAACACCTCAATTTTTTTATAGAGTCTAGCGTTAGTTTACTATAAGATTTGCTTACATCATACTTGCAAAGATCATTCACATAATTTATTTAGCATTGAAAATCTTTATATGAAGTACTAATGTCATCTGAAGTTTAGAGATTAAACAACCAAGGTTTAGAAAGATCAAATGACTAGTCCAAGATAATATGGCCAATAAATGGCTCTAAACCTCCTATGTTCTTTATACTATTCACTTTTTGTCATGTTATTTTTATCCTGTCACTGCCATTATCTTTAAAACTTTGACAAAAATTTAATTTTAAAAGTTACATTGGGAATTTTCAGATTTTAGTGCAAAGACATTGTTGCTGTTCTTGTCAGACCTTTTCAGATACCTCCAGACTATAGCCCTTCCACAGGGTGATATTCAAACATTTTCCACCTAAACATATAAGTTTGCACACCCTGACCACAGACACTGAATTCACTTGATGTCAGAATGCTAGACTAAAAGTTGATCTTCTTGACTGACATTTCTAATCTCCCTTTTTAAAAATCCATCATAAGGACTGACTTCTGAACAGTTGAATAAAGCAGTAATCAATCCAAGAAACTGCATCCATTCTGATTCAGAGGAAAAAAAAATCCAACCTAGTGACCATTTTTCAGCATACTGTATTTCATCACATTTATTCTGTGATTAGTGGAGGACAGAGAAGAGGGACTGGGGAGAAAAACTGCCTTTATTTTAAGAAAGCTCATTTTCCTGTAACTATTCAAAAAGAATTGATTATCAAGTGTTAGATAACAATATTACTAACATTTATCATCATTCAAATATTTAGTAAAATTTCTCCATATATTTAAAATTTTACTTAAGAATTTTCCCAGTTAAAATATGACTGCTTAGTTTGAATAAGGAAGACATTCCTTTCAATTCATGACACAATAGTACATTTTTATTCTTTTCTCAGACAGCAGAGAGACACTGTTACTGCTTTCCCTTGTAAGTCAACAGTCTGTGATACATGCTGTACTTTTACTGATTAGCAATGCAATGTTACTGTGAATATTTAACTAGAACTTATTATATTGGAATTTAAAATTTTCTAACATTGAATTTAGTTCTCTTCATCAAATAAGCAAGCATTATTTTTATGAAATGAAATAATCATTCTTAAGCAGTGATGATAATGGAATTCTGAATACGATGTAAAATCAAACATTCTATTTTTTCTGTTTATCCTTTGACATTGAGTTGTTTTAATTTATTACATATTATTAAGTTGAAATAAGCTTTATACCATGTTCTCTGATGTTCTTGTCATAATAATTTTGGGTCTTTTGTTAAGTAAATATCAGAAAATATCTGTGAGTTTTGATTATGTGATATTTTCATCTGATTGATTTTTTAAATTTCTCATTGGATTTAAAGTGTTTTCTTTGCTAATGTTATATACAGAATTCAATAAAAGACCAAAAAAAA |
| KCNQ1OT1 | Nucleus | AGAACGGTCGCCGCGTCGCCTCAGCACGGACCTCCAGGGAGCTCCTCAGCAAGATCCTGCCAGGGCGCCCCTCAGCGCGATTCTGCCGGGGTGCCTCTCAGCGTGGTCCTCCCCGGGGCTCCTCAGCACGATTCTCCCGGTGCGCCCCTCAGCGCGGTCCTCCTCGGTGCGTCAGTCATCGTGGTTCTCCCCGGCGCGCCCCTCGGCGCGGTTCTCCTCGGGGCTCCTCAGCGCGGCGCTCTTCTGGGGGCTCCTCGGCGCAGTTCTCCCCGGGGACTCCTCGGCGCCGTTCTCCTCGGGGCACCCGGGGCTTTTCGGCGCGGTTCTCCCCGGGGGTTCTTCGGCGCGGTTGTCCCCGGGGGTTCTTCGGCGCGGTTCTCTCCGGGGGCCTCTCGCCGCGGTTCTGTTCTCCCCGGGGGCTCCTCAGCATGGTTCTCCTCCGCGCGGTCCTCCCTGGGCCTCCTCAGCGCGGCACTCTCCTGGGGGCTCCTCAGCGCGGCACTCTCCCCGGGGGCTCCTCAGCGCGGCACTCTCCCGGCGGCTCCTCAGTGCGGTTCTCCCAGACTCTCCTCAGCGCGGCCCTCCCCATCTCTCTGGGAGGGTTTGAACACGGTCAGCACGGACCTGGGCGGACGGCGCGGGACGGGTGATCACTGGCGTTGCTGAGGTGAGCTGTGTGCCCCGCGGCCGTCCCAGATCACAGGCGTCAGCAGTGCAGCCTGGCCTGGGCAGTGCGCTCCCATCTGCACCTTATGGACAGCGTGGCCAGGGTCGAGGTCCGAGTTCCTGGCCGCGTCCCAAGGATCGGATTCCGGGTCTATAGTTCTCATGGTGGTTCAGAGTGGGCTGAATTGGGATTGGAGTCTGGAATCCGCATCGTGGTTCTGAGTCCGCGCTATTGGGATGGAAGTTGGGAATCCATGTTGTGGTTTTGAGTCAAAGCCCGAATTGGGACCGGAGTCGGAAATCCACTTAGTGGTTCTGAGTCAGGGCCCGAATTAGGATCGGAGGTGGGAATCCCCGTTGTGGTTCTGAGTTAGGTATCCTAGTTGGGATCAGAATTCGGGTCTAGGGTCCACATCCTGGCTGGGAGTTTGCCTGAGTTGGGATCTGAGTAGTTGGGGTCCCAGTCAGTCCGGGTCTGGAGTCCGCATGGTGGTTTTAGGTAGGGCCTCATTAGGGACAGAGGATCGGGAGTCTGAGTTGGGGACCTGAATCCCGGTCTGGAGTCTGCATCATGGCTCTGAGTTGAGATCCAAGGGAGGTCCGAGTTGGCGACTGGAGTCAGGGTCTGGAGTCTGTCTTTGGGTAGAGGTTCTGAATGGGGGGCTCTGAGTTAAGAGATTGGGATTGGGGTTAGAGGTCTCAGTGGGGTATGGGAGTCAGGAATTGAAGTCGAAGTTCTGCCTCTAGGTGGGGATTCTGAGTCAGGAATTAGGGGGCGAAGTAAAGGTTGCAGTCTGAGTTGGGGGTGGTGATCACCTGCCAGGTGAGAGGTAGTGGTAGAAGTCTCTTGGTCATATTGGAGATGAGAGTTGATCAGATGCAGGTCTCAGTCTGGGTAGTAGGCTTGAAGGGCCACCACTATGTTCAGATCCCAGTACCCCACTGTGGTCCCTGAGCAGGTCAAATAGCCTTTTATATAAAATTGAGAGTAAGAGTGCCTTCCCACAGCACTGTTCTGGTTATGAAAGAGAATAATTCATCCAGGTAGAATAGTTCTGTCTTAGATTATCACAAAGTATAAAAACTTTCCTGATAATATCCACATTTGATGTGCATTTACCAGTTCTTTCTTTACTGTGGTGCCCCTTTTGCTAGACCAAAAGCTCCCAACGAGGAACTCTGTTTTGTTTTCTGCTGCCTGCTCTGTACCCAGCCAAGAGCCTGATACACAGCCTTACTCAGTCAGTACTTGAAGGAAAGCAGGCAGGCAGGATTAACCAGATTTCTATGGTGTCTCAAATGATCCATTTCTGCACCTGTTTTCCAGATACTCAGTAGGAGAAAAATTTAAGGATTGAGTTGCTTGATTATGGGTGTATTTTAATGTGTTTCCTAACATGCTGAGGAGAAAAGTGGGTCTGTTTCAGGATTTATGCTCCAGCTAGAGGGATTTTTTAAAATTGCGAATCAGAGCATGTCCAAATTCAGATAGAAAGCACAAGAAAACTATAAAGAATTTCCCTTATGAATGTAGATGCAGAAGTTCCTTGTACAATGCTAGCTAACTGAATCCAATAATATGTATTAAACAACAATTCTTAAACAAGTGGGATTGCTCTTAGGAATGCAAAGATGATCCAATATCAGAAACTTTATCCATGTAATTCACATTAAGAGGCTACAGGAGAAAAACCATATGGTTCCCTCAATAGAGGCAGAAAAAAATGATGTGATAAAGTTTAACGCCTATGTGTGATTTTGGGGTAGTGGAGTACACCACTTAGCAAGCCAGGACTAGAGAGGAACTTCTTCAACTTGATACAAGTTATAAACCAAAACTTAACTTTTCAGTAAACTTGATAGAGAAATTAAAAGTGTATCATTCTCTTTAAGATAAGGAGTAGGCCAAGGATGTCATCCTGGCCATACCCTAATACAAGAAAAAGAAATGGGGATGTGAGGATCAGGTAGGAAGTGATAAAACTTAGTGTAGCTCATAGGGTTGATGGTCTCCATAAAGATTCTCAAACATTTTGGTGTTACGACTTGTTGTATTCTTAAAAGTTATTGAAACCCCCAAAGGGCTTTTCTTGATAGAGATTCTATCTGTTGATAGTTACCATCTTAGAAGTTAAAACTAAGAAATGTTTAAAGCAGAACTATACAAGCACATATTCCACTGGGGTCATGGTGTGCTGCCTCTGGAAAACTCTTTATCTTTGTGAGAATGAAAGAAAAGGCAAATAATGTCGAGTATTATTATGAAAATGTTTTTGACCTTTCCTGAAAGGGTCTGGGGGGCTCTCTGGAATCCCCAGACTACACTTTGAAAATTACTGGGCTACATGGAAAAAAAAAATTAAGGAACTTAACAACTAAAGAGAGATTTCAGATTTCAGAGAGGTTTTGGATATAAGATCAGTGTACTCAAAACAAAAAACAAAAACAAACCACATTTCTTTATGCCAATAGCAACTGACTAAAAAAAAGCATAATAGAGATTAGGACAGCAATCAGAATAGCAGCAAAATTTATACCAGTCTAGGAATTAACAAAGACGACGACTTTTATGGAAAAAATTTTAAAACTCTATTGGAGGATATAGAGGGAGATCTTTCCATATGCAGAGACATTTTATTTTCTTGAACAGGGCAACTTGGCATTAGAATGACGTCAGGATTTTCCAAATCTGTTAAGTTCAACAGGACTCCACTCAAACTTCCAGTTGGAGGGCTTGAGTAATTTAACAAACATATTCTAAAATGTATGTGGAAGAAAAATATTTGTAAATGCCAACTGAACTTTTGAAAAGGAGAGCAGAGGACTTCTGCTGGCAGATACCAGGGCACACGCCAACTTCAGCAATCAGAGCGGTGTGGTGACAGAGGCCCAGAAGAGAGCTCAGAGGTAACTCCAAGTCTAGAGGAAGGGGACATGTGATAAAGGTGGCACCACATATCAGTGGGGACTGTTCAGAAGATGATGTTGGAAAGCCAGCCCTAAAATGGAGGAATAGGAATATGGATTCCTAACTGAGCCCCTAGGAAAAGTAGACTCCAAATGGACTAAATATTTGAAAGCAAAAGGTAGTTGCCATTTGTTTAATAGGAGAAAAAATGAATAATCTCCAGGGCCAGGGTGGCCGTGGGGTTCTTAAACAGGATCCAGAAAGCACAAACCTCAAGACTAAAATGTATGAACTTGAATGCATTAATGATTATGGATTAAGGATTTCTGTTAAATAAATCCATCATGGCAAGGGTCAAGACTGTTTTCATAATAATACTAAGGAAACAATTTGCGTTTTTCACTTTTACACTCATGAATGTAACTTTGCCATGACTCATTACAAGATATTTGAAACAATTAAAGCTGAGGAGGGAACAACAGCTAAAATATACCAGAAAGCACCACAAATCATTAAGAAAAAGGTAGGAAGATTGCCAGTTTGCAGGAGGGTTAACAAACAGATGAAAATTTGCCCAAGCTAATCAGTTATCAGAGAAATGTAAATGAAAGCAACAGCTATTCTTCATAGGAGAAGCATTAGAAAGTAGGATAAGGCCAAGTGTTGGCAGAAGGCAAGGAACAAGCCTCCCTGTGTGCTGCAGGTGGGGACGGAGACAGGCACAGACGTTCTGAAGAGCTGGCCAACACTTCTTCCTCAAATAAAATATTTTATACCTGTGGCCCAGCAGTTTCCCTCCTAAGTAAACATGCTGGAGCAGTTCTCACCCAGAACAACGAGAGGCTGTGTGTGTGCATGCTCAGGGCAGCATGGCCCATAGGGGTGGGCAGTTGGAGCTGGTCACTGTGGTTAGGAGCAGGGGACTAGATGTACACTTCACAAGATAGAGACGCGTGGAAACACAGTGCTGAGTGACACAAATGCCAAACAGAAAGAGGGCCTCAGTACATGACTGTGTATTTAAATTAAAATTACACACCACCCAGACACGCAGTACTAGGGAGTGCTCGTGAGTGCACACACACACACACAATGATATAAATAAATTACCCTGGAGAAGCTGTTTGTGTAGGGAGGAGAGCAAGGATAGAGGCTAGAGATGAAGTGGAAAAGCATGGAGAGGAAGAGAGCGGTGACTTTATGCAGACCCTTACAGGGTGACAGTGTTTCATAGGCTGAGGGCTGTGATCAACTCTGTGCAGAGTCCCAAAAGCCAAGAACCAACGAATGAATGTGCCTCCATGACTCTCCTGCCAGATACCCTCTAGTGGCTTCTATTTCTAGGAAATAAAATTCAAATTCCATCAGGTTTTTACAAGATCTGCCCTGCTTACCTCTCTGACCACTGTCCTCCCTCACTGAGCTTTGGCTCTGCCAGCCTTCTTCTTCTTTGTTAAATCTGCCAAGTATCCCTCCTGCCCCAAGGCCTTCACCACCTCTGCCTGGCTTGTCTTTTCTGGTGATCTTCCCTTGACAATTCCTTCTTCTTATTTAAATCTCACAGGAAATTTTGCTCTGACCTGCCATATCTGTCATCCCCTTCAGTCACTCACTAGCTGACGACTACCTTTCGAGTCTCTGCAAAGCACAGGCTACGCTTTTCTAGTTCCTTAGACATCTACTTATTTGTTGGCTGCCTATCTCTTCCACCGGAAGAGAGGCCCCAAGAGAGCATATCTGTCTTCCGTATAGGGTCCGCACATGGTGGGTGTGTCACAGATTTCTGTTGAATGGTTTCATTCATTTATTGAACGAAGGAAGAGGCCAGGCCAGGAAAGTGTGCCTCCATTTTCACAACTCAGTGAGAAAGCAAATAGCATGTCAAAATCTGACCCCTTGCAACCAGGGTTTCTTAGCCAGCCACACTGGTAATGCTTGGGACAGGATGTATCTTTGTTGTGAGGGGCTGTCATTTATATTGTAAGATGTTTAGCACCCTGCCTGGTCTCTACCTAGTTGATATTAGTAATTCTCCCATAGTCATGATAGCCAAAAATATCTCCAGACATTGCCATATGTCCCCTGGGGGGCACCGCAGATGACAACCACTTCCTTAGAACAGCTCTTGGGACTGGGATGGTGTCTCTGCTGTGGCTCTTTAAAGGCAGCAGGCCCTGGCTTGCATCCTGCCACATCTAACACCTATAGCCTCACCACCTCGGTGAGAAAGACCCTGGCCCAGGCCTGGCCAATCAGAGTACCCTCTTCCCTGGTCAGCAGTATAATTTGTTTGGCAAGCACTTCCAGTCCCACAACCTATGGGCTGCCACCCAGAGGTTTAGTTGGAACTACGTGTCAGCTGCCGTCCCTCAGCCATCTATGGCATGGGCACCTGTGGCTTGACCAAGTTGGAGGTGGCCTAGAGAGGCCGGGCTGGGCCTGCTCACCTTCTCCAGTTAGTGCAGACTGATGAATGAGTGGTTCTCAGCGGATACCGGATCCAGGTTTGCAGTACACTCCAGGAGGAGGACGAGTAGGGTTTCCCTCAGAGCAGGCCTGGATGGGAGGGCAAGGGGTGGCCTCTAATACTGCCTTTCCTTCCGGTGCCTTTATCAGCCTGTGCCATGCTCCTCTGAACTGGCCGGCCCCAGCATGTCCAGGTTACTGGCAGGAGCCTGTATAACCCAGGGTTCCAGTCACACCAGCCAGGAAGGCCCATGAGGCTGGAGCGCACCCAGTCTCTGGCTGCTCTGTCTGCGGAACTAAAAGCCTTTCTCCCAGAGCCTTTCCTACGGCTGGGCGAAGCTCTTGTTTGCAATTTAAATGTGGCCTCATTTATGGAAGAATCCCTGCTCCTCCGAAAAGCCTTGGGCTCAACCTCCCTAGCAGCCCGGGCCTCAGCCAGGAGAAGAAATGGATCTAAAAGGCCTTTCTCCTGCTAATTGTAGCAATTAGCATTAGAGAATGGGGACTCGATGGGGGGCCAGCGCCTGGCAAAACAAGCTTATTCCCAGCTTAGGCCCAGGCCCAGCTCCACACACTGCTGCTTGGCCAGACTCGGCCCCCACCCAGGGTCTGGTTGGCTTCAGGGCAGGGAGAGAGGGCCCAGGCCTGTGGGTGCTAAGAGCAGTTGTGGAGGGTGTGCATCTGAGTTGTTAAGCCTGGTAGCTGTACAGGTAGCCCTGGGCTATGACTTGTGGTCCTCTCAGAAAAGGGTCTGACTGGAACGCAGAGCCAGACCAGATATTCATGAGTGGATCACAGAGTGTAGACTGAGGGCTATCTGGTGATTGACAGAAAGTAGTCTTGGGGCAAATGACTCTTAAAGAGTAAGGAGCCATTCCCCTCTATGGCTAACTGGATGCCTTTCTCTCCCCCTGCTCCAGAAAGGACTTCCTGTTCCTTCACTGAGCGTGCTTGCTTATGAGGCTTCTGGAAACCAGGAGAGTTCAGTGGGCCTCATTATTGAGCGTATGGGCAGGACAACCTGCTTGGGACATAGGCCATGCTACAGTTGATTTTAGCCAGCTTGCTGCATCTACACAGCACAGTGCCTTCCCAGGCAGTGAATTATGTCTAACAGGCAGGGGAATGATCATTGCCTCATGTTAGGGATGCAGTCATTCAAGAAATATTTGTCAAACACTCAGCAGGTACTAGACCCTGGCCCAGAGGGAGGGATTTGATAGGAGCAGGGACCTGCCTACCCTCCCAGGGTTAGTGGACAAAGGGGAGAAAGACTAACTAATGTTGAAGGACCGTTTGCACCCTGAGGGGAGTGCTGAGGCCTGGGAGCACAGCAGGTGTATAGCAGGAACCCACTGGAACTAGCCTGGGGTCTGAAAAACTCCCTTGGGGAAAGTGGCAGGGGCAGGCCAAGGGCCCTCTGGTGGTAGGGAGCCTGTGAGCACCTGGGATGGAAAGAAGCCAAGCATCTGAACTACCAGAAAGGCAGCTGCAGGATTAAGCAAGACCCACAGGTCTCTAAACTTTGAAAGGATGTCTGTCCACTGAGTAGATAATTGATTGGAATGGGGCAGGGGATGTGAGAATGCCAGTTAGGAGATGCCTGCAGTACAGAGAAATGGTGGCCTATCAGGACAGTGGCAATAGGGATGGAGGGAAGCAGGGAACCTCTAGAACTATGGATGGGTGAAGTAAGCCTGGCTTGGTGATGGGCTTGAGGTGATGGGCAGGTGGTGAGGAAGCGGGTGTATCGGAAATGACCCCAGAACTTCTGACTTGTGCCACTGAAAGGATGGGTGACACTGAAAGGACAGGGGCCTTCAGTAGAGTGGGCTATAAAATGATGGAGGCTGTGGAGAGGAGGTGGGGGCAGGTGACTGGAATGTGGGTCTGGCTGGGTTGGAGAGACATTTGGGGGGTCAGGGCCTCACAGGTGGTCTGGAAGCCCTTGGTGCTGAGGGAACAGCTTAGGGAAATGGTTTGCATAGAGAAAGGCACTGGCATTTAGTGGCCATGCCAGAAGGGAAAACCTGCCAAGGAGACTGAGGATAGAGCCAGAAAGATGGCAAGAAAGCCAGGAGAACTGGAGGGATGGAAAAGAGGACCTAGGGGTCATGGTCCTGGCAGAGGTCACATTGGATCCAGTGCTTGTGATTCTCTGATTGCTGGCAGTGGATGGCCACTGGCCTGCAAATGGCCCTAGTGGAATGTCCCCTGCCTGTGTGGCTCCCTGTGGGCTGCCTCTCCCCTGGACAGATGGGGAGAGACCCCTTCCCCACCTGGTTCTCTGTTTCACCCTTGGAACTGTGGCGGTAAAGAGGACCTCCCCACAGTACGTGCAGGGAACAAGCTAGGTTGACTCCCTCAGGGAGGCCTCGTCCTTGCTGAGGTATTTCATAACCTCAGGATAGAGAAATCCCCCCAACTTTAATGCAAAGCCAAGCAAGCTGAAAAATGGCCTTGAATAGGCTGAGATGAAAAAACAAAGAGGCTGTCAACATCAGAGACTCAGTGGGGGCAGGGAGCTTCAGTGGACTCCCCACAGACAGCAGTTCCTGGGCTTCTATGCCCAGACCCAGAGGCCTGAGCCCAAGATGTCAGGATGGGTGGTCACTGTAAGTGCAGTGGATTGTAGCTCAGGGGTGAGACATCGCCTTTTTCTTGTCAGCTCTCCTTCCTGATTAGTGGCTCAAACAGCCTTCCGAGACTCAAGGGGTTAACTGCAGATCCTCTAACTCCTCCACTTGCTAATTTTTAAACACAGCTGAGCTCTGAGCACCCCTCCCAAGGCTGTGAGAGGCCAGAGGCCAGCACAGACTCTGCACCAGGTCTGCACAGCCTCCACCCCTACTGTTGTCAGGAATCTGCAGGCATCTAGAACAAAGGTGCCTCCATTATTTCCCTCACCCAGCATATCCTGATACCTGAGCCAAGAGTTCACCCAGAGCTTTTGCCCACTTCATTGATGGTCTGGTGGGCTTTTGTTGACACATACCCGTGACTCCCAAATGAACCTTTCAGGTTGGCTTAAAGCCGCTGGGACCTACACATGACAAAACCCAGACGGAAACAGTGGCTTACACTCCTGTGTCCCTAAAGGTACCTGTTCCTAACAGTGGGAGTGCCAGAGCCTGGAAGCTGGAAGCCAAGGAGACAGGGACCTCCCTCCTGCTGACCCCCTGCCAGGCCACATCCACCTAAGGGATTTGGGATCATACTCCATCTTCAACCACTGGGATCAAATGGAAAGCCCTCAGGAATTCTGAGAGACAGCCCCTATACCTATGTGCACTGACACACATACACTGACAAATACGCATTCTGACACATATATGAACATGCACATATGTGTGCCCCACTCCCTGCCAGGAGGTGGGTTCTGTCCCATCTTTGACATGTTACTTCTTGTCCTTCCCTAGGCCCAGTGGCACTTTCTTTGTGATAGGTGCTGAGACCCCCCCGACTGGGCCCAGTGGCTGTTCCCAGGACCCAGTCTCTGCTCTTATCTTAACCCAGCTCTAGCTCCCAGGACCCCAGCCTTTCGCAGGGAGAGAGATAGCTCTTTTGGTCTGGCACATGTTCCATGCCAAGTAGTGGAAACAGCAGCTGCAGGGCAGCCTCAGACTTGACATCATTTAATCCTTGTGACAATCCTACAGGGAGGAAGACACTACACATATGTGCAGATGCAGCAAGGAGCAGCTCAGCCACATGGAATGACTTGCTTGAGGTCACCAGGCACTTGACACAGCAGAGTTTAGCCCAGTCTCTGGAGAGACCAGCCTTCCACATCCTGTCATGCTGGGGAGGGCTGCCCGCTTTTTGTCCCTGCTCCCCCAGTTCTAACCCCGGAAGAGCGATTGTCCCTGCTGTGCCTTCAGCCCAGACAGAAGCAGGCTAGGAGAGGGGAGGAGAGGCAGGGCTGGCCCTCGGAAGGCTCACCTTCCTCAACAGTTCTCTGCTTGGGCAAAGGTCTTCCAAGCAGCCAGGCTCTGAGGCTAGTCACTGTCATCGTTGGGTGAGGCAGCCTGGGAGTACCAGAAGTTGGAGCAGGTGGGAACCTTCCCCAGGGATGGGGCTGGATAGGCAGGGGTGGGTGCTAAGTGGCAGGCCACAACCAGGCACCTGGGAGGGCCAAGCACCACACTGAGAACGCAGTGCTTCCCCTCCCCTCCTAGGCTGCTCAGGAACCCATCAGTCTCCTGTAAACAAATGAGACTAAGTCTCTGGCACTCTAGGCCCAGGTGCAAAGGACAGCTGCCCCTGCAGCTACTCACTCTGCCCTGGCCCTCTCAGGAGGCTTGTGACCTCTGCACCTGCTGGCTTGTTTTGTTCCTAAAATACCCGGATCCTTGAGAATCTAGAAGACACCACTGCTAACAGATTTCCACATCATTCATTCTGTTCTTCCTGTGCAAGCTTCATGCTGGGACAGGGACCATAAAACAAGGTGACCCAAAACCTTCAGCAGAATGCAGGTAGGCAGAGCAGAGGTCTCTGGGAGTGTCATTCCAAGGCAGTGTCTTCCTCCTCCCCTATGCTCCCCATTTCCCAAGAGTGGGCTTCTTCCACCCCACTCAAGCACCTCTTCCCAAGGCTGACCTAAAGGCCTCAAGCTCTGCGGATAGGGGCAGTCCTGAGGTGGCATTGAGTCTTAGGGTTGGAGTGGGAGATATCTGTGCAAAGGCTGATAGGAACGTCCTCCAAGGGCTGACTTCAAAGCTTGGCCTAAACCACTGCATCAAAGTCAGCAAGCTGAGCTGTTTGCACAGAAAAGGAGGAGGAGCAGGCCCTTGAGCCACCTGTAGCTCTCCTTAGGGTCTCAGGCTTTCACACCAGGGCAGGCCTTGGGCCAGACTACCCAACTTCCAGGCCGGATGTGCCACCGGGGAGGAGGAGGTAACTGCCCCTCAAAACCCTGCAGCCCAGGGCTCCAGGATTCCAACCCAGGGACCTGCAGGCTCCCCTGCTCTCCAGATCAGACCCCTACTCTCAGAGCCCTAGGCCCAGTGGGTGACAGCCTGTGAGACAGGCCTGTGTGGGGTGGCCCTAGCCCCACCTCAGCTTCCAACCTGACCTTGCTGCATGACCTCTGGCCCAGCCCTCTCCACCTGGAGTGCCATCTCTCTTTGTGGGATGGGCCAGGTGGGCTGTGTGTGGGGGAGGGCAAGTATATAGGTGTGGTATAGGTGGAAACTGCCACAGTAACCAGCAGCAGCTCAGAGCCGGGCCACCCAATTTGTATGCCAGCCCCAGCTGGGCCTAGGCACGTGGTGTGTGGAAATGAACGAAGCAGGCAGCCCTATGTTTAGGAGGCAGGTCCAGAGCTCACCCAGGCACAGCACAAGATGAGTGCCTGGCCTCAGTGAGCGAGCACTGGCTGCTATGAGGCTGTGGTGTGGAACCTTGACACTAGGGCCACTGGCCTGGCTGTGTGACCTCTACCTCATCACCCAGAGCAGGGCAACCAGAGGCAGAGCCTCAGTGTGGTGTTCTCCTGTGAAATAGTGATGGCACAGGCAGGGGCTGGGGCCCAGTGCCTACACAGAGGATCAGCAAAGGATGGCAGTTATTGAAACCTCTACGGGGAAGTTCCCCAGACACGAGAGGAAGACCCCCATGGAAGCTGTGTGTGTGACGTGCTTAGATTTTCCAGAGTCTTCATTTTCCTTGTCTAAACAGAGGTCATGGCGCCTCCCTCTCAGATCAAACAGCCCTGTATGAGTGGCTACAACCTTTGGTGGGCTCAGTGCAGCCCCTCCTGCCCCCTTGCTTGGGAGCTGTCTGCAGAGGCCTAGGGAGCTGCGTGGGGTCTCTCCACCTCAGCCTGTATCCCCACCCCCACCAGGTCCCCTGCCTGACTCCAGCATACCCAGGCCCTAGAAGCACCAACTCCATGGCTGCAGCTGAGTGGAGTGTGTGCCTGCCCAAGCCTAGAGGGGTGCTGACCCCTCCCTCGCACGAGGAGACATCAAAGGGAAGCAGGAAGCAGCGGCCTGGCTGACCCACAGCGGGTGGGAGAAGTGCAGCCTCACAACTGCTGGGGATTTTCCATCCCACCAGGATTTCCGCGGGACACCCCAAGTCCAGCCTTTGAGGCTCCTCAGGAACCAGAGTTTGGCCTGGAGCCAGTTGGGGCAGAGGAGGGGGGCCAGGCTGGGTGGCTTAGGGGTGTGGTTAGGCCTGCTAAGGGGCTCCTCTTCTCAGTCTCCCCATGATTTGCTGGTTACTGGCTTGAAATCTGGGCCCAGACCCATTCCAACACTGAACCCTGAGCTCCCTGAGACCTGGCTGGCAAGGACAGGTCCCAATTTTTACAGGGGCCTGAGAGGAATGGAGAAGGGATCTCTGCCCCTTCCTTTCTCAGTCACACCTCCTTGACAGGGGGTCATCAGAGTGGGCTGTGGTAGGTGCACCAGGGAAGGGACCATACAGAGGAGGCTGGTCTCAAGTCCTAGGGTGCCTGGGATGGCCCTATTGAAATGCTGCAGCAGCTCTTGTGCAGGCAGGGATCTGTATGGACAGCTGGGTGGACACAGGTGCCTACTGACCTCCCAGATCCTTCAGCAGAGCTCAGCCCTGAGTGACAGCCCAAGGCAGACCTGGCTACCTCAGCCTCTGGCTCACAGCCCTCAGCCAGAGCCCTGAGAGGCCTGGGATCTCAAAGCTGCAAGGTGGGAGGCCAGAGGAGAGAAGCCCGGAGCTGGTAGTGATGCTGGCTGAGTGAAAATTGCACATTTCCCAACAGTGTGATGCTCCCTATCCAGCCTGGGCTTTTGGCAGACTTCTGTGCTTTGTGGAGGAAGCTGGCTCAGATTCCTCCCCTCCCTGTGCAGTTTGTGTGTCAAGGTGTACTTGTCACACCCAACTCAGGATATCTTGCATCACTTTGTGGGTCCTTGTCCCCCAGAGCTGGGACTGCTGAGTTTTAGCCCTAAGCTCTTGCTCCCATCTTAGCAAGTCAGGGCCCAGTGTTGTCCTTGCCTCTGCATCAGGATGGTAAGCTGGGCATGCTCCGCATCACGGAGACCTGCATGGACCTGGATTGGGTTGCCTCTATCTTCAGGCTGGTCACTGGGCAGACACAAACCAGGGCCTCTGGCCAGAGGGGACAGTGGCTCTGAGTTACTTGTGCTGCTCTTTAGGGGTGAGCAGGGGCTGCTTGTGCATCATAGTGTATCACAGGGGATGCACATTTACAATTGAAAGCTACTGGTCCTGGGTAGAAGTGGGGCCGAGTAGGACTTCCCAGCCTTGAACATGGGTTTGTCATCCAACTGGGACCCACACTTGATCCAGCGGCCATGCTCTGTGCAGCCTGGGCCTGAGGGCAGCCACGCTCATGTGAAAAGTGCTAGTAGCCAAGCTGCCATTCTAGTCACCACAGCTGTCCTTGCAGGAGCACATTGCCCAGGGCTGGGAGCTGTCATTTCCGTGACGGGAGTGAGACTCAACACTGGGTGAGGGTGGGGGTATCTGTTGGGAATTATTTCAAAACAATTGTGGAAACTTGGCAGTGGAGGGGCTCTGGGCATAGGTATCCACAATATCCTAGTCAGGGGTTGGGGTACCCTGGCTCTGCTGATAAGCCCAAGTGAGCATACTCCTCCCCCAGCTTGGAACAGGTGGGACGGGCCAGGTGACACTAGTGTGGTCTGTGGTCTGGCCTCCCAAGCCAGTGGCAGTACTGGGGTGGCAGACATTTGCTGTGTGCTATCCCAAGGCCTAAAGGTCACACAGCTAGGTTGGAGCAGGGTTTTGGGCAGGATGAGTAGGCATTGCTGTGCTCTCAGAAAGGCGAAGCAAACCCCAGAAGCAAGGGGGCGAGACAGGGCAAAGGCCTACACTTTGCCATCAACTCAGGCCTACTCAATGCTGGCCCTGTCCTTCCCCTGCCCCTGCCCCATCAGATGGTGCGAGCTGGCGGGGCTTAGAGTGAGCCCCCCTGACTCTTCTTGGTGTGGAGTCTGAGGCCCAGAGTGTACAGGCCTCATCCGTGTGACCCAGCAGGAGGATGGGAGAGTGGGCCCAGAACTTGGTCAGGCTCATCCGTGGAGAACAAGTTCTTCCCTAGTCTAGTTGTGGTCTGAGTCCTGGGTGAAGCAGGAGGCAGCCTGGGAAACAGCCTCGTTCTAGTAGGAATCGCATTCTCAGGAACGCTGACTGTGCAGGGCCCTTTCTCACTGCCACCTGGGAGGATGCCTGCCTGCTGGCCATTTCGGCGGACTGTCAGAGCACCCTCCCTGGGGTCCCAGACCGAAGGCCTGAAGTCCACATGGGACCCCTGAACTTCTGGTACCTGTGTGGGCTTGTCCCCTCAACCCTGAGTGGAATGGCAACAGCCAGCTGCAACCCTCCTAGCTTCCTGTCATGTGCTGTATGGAAGGGCCAAGAGCTGTAGTCATCTGCAGATCCTCCCACTGCACCTGTAGGACTAGCGTCTCCTGTCTGGATGTGAGCAACTTGCTGGTGGCCTGTGAGATGGGAGTTGCACTGGGCTTCTGGATAAGGGCTTCTCGTTCAGGTGACAATTCTATGTGAAGGAGACCCATGGAAACACATCTATCCCCTGCCCATGTACCAGGCAGAGATGGTACCTGCCCATGTACCCTCCATGACATGGCTGGGTCCCTGAAACATCAGACAGATCGATACTCTGTGTGCATGTTTGTGGGCAAGACACAGTGGCACGTGTGCCCTGGAGTTTGGTAATGGGAGGACTGAAGTAGTGCCTCCGTGTGTGAGCTGTGCCAGCCATCCCCACGAAGGGGGCATTCCATGCAGATGGGGATCCTCTCCAGGCAGCTTCTTCCACACCTGATTAGCTCTGACCATCAGACCCCCCGTGCCAAAAGGGCCCCTTCCCTGGAATCTGTAATTCTCACAGGCTGTCCCATACATTTTAAATCCAGGAAATGTGCTCACCCATGAGACAGGGATGGCAAGAAGATATGAATGAAAAGCAACCAGTCCGTAAAATTGGAAGCCATTTCACTCCTTACCAGGTACAAAGATAACATGCTGCATGGATCAGGGACACAAACTTACCTACCTTATGATGTAGTGAAAGAATCTAAAACTTTTGTCTCAGCAGACTCCCTGGAGGAAGCCAATAACAGGAGGCCTTGACTAGGAGACCTCAAGCCTTGGAATGTGCCCAGACCAGTGGGGAGGGAAGGCTGGGAGGCAGTCCTCCCTTACCCCTTTTCTCCCAGGCAGACCTACCCTGGGTTCTCTCCAGGCCCCTCCGCCTGAGCAAGTGCTCAGTTGGCCACTAGCTCCCTGCACATCCAACAAGCCTTTCTTTCCTGTTCCTTCTTCAAGGAAGGCTTAGCCTCAAATCCAGGGCCACAGACAAAGGACAGTCACTCTCCTCTGCCAGAAAGTCATTTATGGGACATGGAGAAAGCATCAAATATATTGCTTTTCCAACACCTAACAGTGGAATAGGAGGAATAGAAGGAGAACTCAGCCAGAAAGTTCTGGAGCATAGTCTTTTTGCCAAAAGAACACGTTTTCAAATGAAAGAGGTGGTGCTGGGGCACTGAGGAATCAGGGGGATGGACCCCACTGGAGCTTGTCTCATGCTGGCAGCAAGTCCCAGGCCTCAAACACATACCTGGGCCACCTCAGGAGAGAGACTGCAGGGAAGAAATGAGTTGCAATGCTCCAGCCTAACCAACTCCCCTTTTGTTAATCCAATCCCAGAAGGTACTGGGTAGGGTCAGTTAACAGACAGCTGCCTGTCTCCTGGCAGAGTCTGACCAAGCCCCAGTGGGCGCTTCTCTTCCTAAGTCCTCTGGTGTACCCCATTCTTCAAGGGAAACGGTCCCCTTAAGAATCTATGTGAAAGGGTTCTGAAATTGCTGTACCAGTTAATGAATTATTTACTCTAAACAATGTCTTGATAAAGGGGGAGGATGAAGTTAGCTGATGTTGACTAAGGAGAGACGGAGTTGCCAAGGAAATTCTTCTAAATGAAAAAAAAAAAAAAGTAAAAGCAGTCAGCTGGTTTTGTCTGACTATGAAACGTGTCACAGAGCATGCACCTTTGGCCATCTTTTGCATTTATGATTTGGAAGAGAGATGTAGCAGTGAGTGTGGGGCCAGGGAGGGGAATCCAACAAAGCCATTCTGCAAGCTAGTGGCTGGGATTTTGGGGTATAGAGTAAGAGCAGCCAGCCTTAGAGGCCCATGTCATCTGGGCTGCCTCAGAGCTAAAGCTAGGTAACCAGAGGGAGTAAGGAAAGGCCTGAGTCCCAGGCTAGGCTTTTCACAGTTGTATTGAGATGGGAAAGTAAGGGATGTTCCTTGCAGCAGATGCAGGGCCCAGTTGTCTAGAGCAGTATTTGAAATGAGTTGGCATTTCAGGGCAGTGTTTGTGATGCCAGTCAGTAGGTAGGGGCTTCTACCTCTTTTTTAGAAATGTTTACTCTGAACTAAGCTCTATGCTTTAACAAGTTATGTTTTAATAGGAAAACTGTCATTGATGGAATCATATGTAATTCCTACATTTTTCCAGTTACCCATTCCTGGGGAAAGCTTAGCCATTGGACAAACCACCCAGAGCCCCAGGAACCTGCATTTGCAGACAGCAGTGGCAGGCACAGTGGCCTAGTGTTGAGCGGGGGAAACCTGGCCTCTATGTGTCTGGTTTTGCAAACTAAAACGCCATTGCCCATACTCAGGCTGACAGGTTCTGGAGGTGCCATGGACTCCTGCTCTGTATCTCACCTCTAGTTCCATCACAATGCATGAGCTGTCAGTGGGAGCCTTTTAATGCAGATGCTAATACCCATTCCTAATGCTAGCCCATCCCCACACCCTCCTGAGTTGGTGCGATATATCCTGGGAACAAGAAAATCTGAAGGCTCCTGGTCCTCAGGCTAGCAAGAGCCTTGCCTGTAAGAAGTGGGAGAAGGTCACCTCTCTCAGGAGGCAGAACTAAGGCTGCACAGCACAAGGGAGATGTCTGAATGCACAGTCTGGGTGGCAGAAGGACTCCTGTCCTTTCTTCCACACAGACCTGAGCACCTCTCCAGGTGCCACCATTTTCTGTCTGGGAACATCTGCTATCCCCAAGCCTCAGTTTCCTGGTCTACAAAACTGAGATGGTCTCTTCTATACAAGGCCCCAGGAGGATCACTGCAAGTCACTGCATTTCATGGTGCCCAGATTTCCCTGCATTTCTAGAATGTCAACAGAGATGAGTGGGCTCAAGGGGAAGAGATTCTTTATGACTAGAGATATTCTGGTCAACTTTGGACCCCAGCTTCTATGGACAATAGCATGTGGACATGAGCCCTGGGGAAGCAGGGATGACTGAGCCTGAAGAAGAGCTCTCAAGCCTTACACTCTCCAGACCAGCCTTGGGTTGCCTGAACCCCAGCATAGCCTTATGGAACCCCTGCCAGGGTCCTAGCACCTGGTTTGACCTGGGCACAAGTACTCACTCACTCACTCACTCACTCACTCACTCGTCAAACATTCACTGAGGGTCCGTGACAGGCTCCATATGGATTAAGCACTGAAGATAAAACACTGAATAGAAACAGCCAGCCTATACCCTCATGCTCCTTGAATGTGATAGAAGGAGCCAGCCAGTAAACCCTTTACCACAAATAAACACACAGTTACACATTAAGAATGGGAGGGGAGGTATGTGATTTTTTACAGCCCAGTCTTAAGCTTTAGAAATTGGCTCCTCTGAGGAAGGGACATTTCAGCTAAGAACCACAGGATAAGACAGTCCTGGCCTGGGGAAGGGGAGAGAAGGTCAGGAGAGAGCTTGATATGTTTAATACTGAAAGAATATTTGATGAGCTGGGACATCTCCTGCAGATCCACGGCTCCCTAGCTTTGTGACCCTGGGCCCTAGACTCAATCCCTCAACCCTTAGGAGATTATCTATAAAACAGGAGTGATAATATTCTTGTGTTAAAAGCTGCTGTGAGGATTAGTGAAATGATGTCTATATTTGCCTGGCAAACAGGAAGTGTAAGTAGTAGTATCATTTGAAGATTATTCTTCAGCCTAAACGTGTGTTCATTATGGCCGGTGTGTTTTCTCCCCACCTAGATGGTAGTGCTGGGCCTCCTTTGGAGTACCTTGATGTTTGGGGAGCATGGATGATATGACCATAACCTGAGAAGGCAGGGACTGTACCCATTCTGGTCCTCCCAGGTCCCAGGGCCCCCACAGTGCCCTGGTGTGTGATCAGTGCCCACAATACTGAATAGAGGAACAGACACATGGGAAGCAACGGCAGCCTGGTCTTTAAGGGTGAGGGAGCACCTTCTCCTGAGAGAGGGAGAGATAGGTTGGGGGAGGTTGGGTGGGGGTGGGGAGAGCGAGTGAGCAAGCAAGCTAGAAACTTCCTGAAAAGGGAAGTTACTCTAGAAAAGGGTAAGTTCCTTCCAGGCATCATGGGCTGGCTTAACCTGGACCAGTTTGACTTTACTAAGTCCCATTTCTCATCTAGGGAGCCTGAGGCCATTAAGGTTATCTGGTCCCAAAATCCCCTAGAGCTGTCACAGGGATCCCCATTCTTTGCCTTTAGTCACCCCAGCCCCAAGAGCCATCCTCCACATGGAGTGAGCAGGGAGCTACAAGGAAAAGACGGTCACAGTTGGAGAGAGACAGAGAACCAGGTGGGCTGGTAGGACAGGAAGGTATAGGGAGAAAGTGAAGCTCTCTCCTGGGGAGGCAGGAATACTCTCCTGCTTCCTTCTACCCACTCTTCATAGAACACAAACACTGCAAAAATGACTTTTTCCAAACCCTCTTAGCATTCAGAAGTAGAATTGCTATGTAAGAATTTTTTCATAGGTGCTTTTTTGGGGGGCATCTATTTAAAAAATAACCACATCATTAGTGAAATCTTCCAAAGTCTCTTTTTGGAGCCCACATTTCACTAATGTCCACATCTGAGGAGGCTTTCATAATAAAAGAACACATACCTATTTCACCTATGATGGTCAGTGCAATCTTGAAAACACTAGTAAATAGTGTCCATCAGCTTTAAAAATACAAACCCAAATTGGGTCATACCAAAATGCAAGGATACACACAATAACCTGGGTGAATCTTCAGGGAATTGTGCTGAGTGAAAAGAGCCGATCCCAAAAAGGTGATTTGCTATGTGGTTCCATTTATATAATTTTCTTGGAATGATAAAGAAATGAAGAATAGGTTAGTAGTTGTCAGGGGCTAAGGACAGGGTAGGGTGGGAGGGAATTGGGGGTAGCTATAAAAGGGGGACATGAGGGATTCTTGAAGAGTTAGAAATGTCCTGTAGTGAGACTATCAATGCAATATCCTGGTCAGAATATTGTACTACAGTTTTGCAAGATGTTACTATCGGGGGAGACTAGGTAAAGGAGTATGTAAGATTTCTCTGTGTTGTTTCTTAGAACTGCATGAGAAATGTACAATTATCTCAAAATAAAAATTTATTTTAAAAAATGCCCAAATTAAAAAAAAATCAGTAGTCCTCCTAAAAATACCCAGAAATGAATTTTAGAATGTCCAAAATCCATACAAAGATCTAAAAAAATATGGAAGGATGTTGCAATGGATTTGAACATGTGGGTAAACTGCTTCTATTTGTGGCTGGAAAGACTTTTTTTTTGAGGTGGGGAAGGCAATTCTTCATCAGTTAATCTAAATTGAATGAAGTTTCAATAACAAATGCCAATGGGATATGAATATACACACAGATAGACAACTTTTTTTTTTTTTTTTTTTTTAATTAGGCAAGCTGAGGCCAGGTGCATTGGCTCACGCCCGTAATCCCAGCACTTTAGGAGGCTGAGGCGGGCAGATCACTTGAGGTCAGGAGTTTGAGACCAGCCTGGCCAACATGGTGAAACCCTGTCTCTAATAAAAAAAAAAATTAGCCAGTCATGGTGGCAGATGTCTGTAGTCCCAGCTACTAGGGAGGCTAAGGCAGGAGAATTGCTTGAACCCAGGAGGTAGAGGTTGCAGTAAGCCGAGACTGTGCCACTGCATTCCCGCCTAGGTGACAAAGTGAGACTCTGCCTCAAAAAAAATAAAAATAAAAAAAAAATTAGGCGTGCTAGTTCTAAGTTTATATGAAAAAAATGCATGTCCTATAAAATTCAGAAGTAGCAGCAGTCTGGCCCCTTGTGCTCCTCTAGCCCTCAACTGGCCTAACAGGCCCTAGTGTGCAGCAGGGACTATTTTCCCATCATCACTGTCCACAGCATCTGGACAAGAATACAGAGAACGGCTGATTTAAAAAAAATCTAATCTGGGATCCTATGCATCCTAAATAATCTTATGAGGATGCACTTGTATTTTTTCTGATGCTGATACTATTCATTCCAACTCGCCTCCCCATCAGCTTTGTCAGGCACTGTGAATTGCATGGTACACATGATTACAATGGATGGAATGGCCATCATGAGGTATAGCAATACATTTAAGTAACACTGTGTCACTACTATAGGACAGGCACTTTTCTGCATACTTTATATTATTAATTTGTGTAATCCTCCTAACAACCCTCTGAGGAAGGTACTCTTATGATTCCCATTTTACAAATAAAGAAACTGAGACTAAGTGACTCACTCAAGGTGTAGCAGGTAGAAAGTAGTGGAATTAGGATATGAGTTCAGACTCCAGACCCTGTTATTAGCCCCTGTCTTTGGCTATGGATTAGTAAAGGAGAGCACAGCCAAGGGTGGGACAGGCAGCTGTAGTCCTCTGGACCAGCTTCACATGGATGGGGCTGCATTGTTACAGGTGGCCTAACTCAGACATGGAAACTGAGTCGCTGCTGCCCAACTCATGCCATGAAACCATTTTGGCCTGCACAGCTTATAACAGCACTCTAGCTCTAGCTATGACACTTTATGGTGTTAGTGGGTTTTTTTGGTGGTTGGTATGGAGGAGGGGCTGGGCTACAAGTTTTAGATTATGTTTCTTATTGCGGGTTTTTAAAAATTCTTGAATTAGGCCTATTTGCTCTTTTGTTCTCTATTGAGTTAGCAAATTCAGTTTTTCCAAGATGCCCTGAAAGTATGATCATACATGTATACGAAATGGTCCTTGGGTCCAGCTGGCAACTCCTGTGCACCCCCGACCTGGATTCTGCCTTCCTTGAAGTGAGCCTTGGAAGAAACAAGTGCTCATGCTCCCTGACATTATTAGCTGCCACGATGAAGCCTCAGTCCCAAACACCCCAGCTTCCTGTCTAACTCAAGAGCTAATCCTGTGTTTAAATCTTAAGAGTGTTTAGATCTTAGGCCATGCCATGAATTCCCCTGGTTGACTGCATTTGTGAAAATGAGCTTCCCATTATTGGCTCTTAAGTAATGGAGATGAGGGGGAGTCCTGCTACACAGATAAGTGCATCATAAAGTTACATAATTAAAATAACTGAGCTCTGGCCTATCAACAGGCCCATCAGCAGTCCAGAATAGAGTCTAGAAATGGACACAAATTAAATGAGAGTTTGAAATATGATAAAGGTAGCAGTTGAAATTAGTAGAGAGATGATGATGTACTCAAATAAATAGTGTTGGACAACTGGACAAATATATGGGAAACCAAATTGGATTTAATTACTCTGTCAAAATCCCGATAAAGCAAGGATTTAAATGTAAAAAATGAAATAAAAAATGATAGAAGAAAACATGGAAGAATTTATTAAAACAATTTCAGGATGAGAGGTTATTTCAGAATTTAAGATAAGGACCTCAAACCATAAAAGACTGACACAGCTGACTATGTGAAATTAAAAAAATTCTGCCACCAGAAAAATATTATAGAAAAAGCCAAAACACCTAAGGAAAATGAGGATAAAATATTTTCAATGAATATGACAAAGCAAGTTTCTACAAATCAGTAAGACAAGGTATTTTACAAAGTCCAAAGGGTATGAAGAAATAGTTCACAAGAAAGAAAAACAAATGATTTTAAGCATTTGGAAAGATAAAAACCTCATTTCCAACAAAAGAAATGTAAATTAAAGCTCCAGTGTACCATTTCTCATCTATGAAATGAATGAAGATAAAAACCTGAGTATACACTTGGTAAGGGTGGGGGAAGAAAGTGCATTTGTGCAGTCAAAGTGATGTAAATGGTACAGCCCTTATTTGGCAGTATCTATCACAACATAAATGAACATTTTAATCCTGCTGGAAATGGGGGGAAAGGGAGGATCTCTTAAAGTAAATCCACTGAAAACGGAGACCAAAATTTTACAATAGAGATATTTGATTGGGAAGTTAATTTGAAAAATAACAGTAGTTAAAATATTATTTTATCTCATATGCAATAACAGTTGTTAGAGTTACAATTATGTACACGTTTTAGAAAAAAACAAATCCAGAGGGGATTTTGGTTTGTCTTTTGTAAAGCATTATATGGTAACATCTGAGGGCATCTTTTATACTTGGCAGAGGATGTTGGCAGCACTTCCACAGAGAGGATCATTTTTTGTAGAAGTATGGCATGGTTTCACTGCTTGACCAAGAGGCCACCTGCATCAATTATCATCTCCCCTGCTGTCCTCTGAAATGCTTATCTAGGCATCGATCTTGGTTTCCTCTTCAACTGATTAACTAACTGGTCTACTCTGCCCAGTCTCTATTTGAGAAATCATTTGACATCATTTTCTTTAACTTGATGAAATCTCCTATTTTGACAAGATTTACAGTTCCTCTTTATAAGTTCCTCTTTACTTTGTGTGTGCTTTATATTGTCTTTGGTTGACAAGAGACAGCCAAAGAGGTCAGTCAGCAGAGATACAAGTAGGGATAAGATGGATAGGGAGAAACAACAGTGTTCAATAGGGAGGGATGTTTCAGGGAAACTAATTTCATAAGTGGTCATTTCAGTAGTGAATATATTTGAATGGTTAATGGCTGCTGTTTTTTAGCTGCCCTACATATCCATCAACAGAGGATCTGTTCCATAAATCATGGCACAATGAAACTCTGCAGCTGTTAATGAAAAGGAGATTTGCTAGATGTCTTAATACAGATCCATCTCCAGAACATACCAGTGTCTTCCTTTTAAGTGGTGCACACTGGGGCTGAACACTTACTAAGAAATGAAAGGGCTCCCCAGTCAAAGCTGACAGGTGTCATTACAACATGACCCCTGTGGTAGATCCCAGCACAATACACTGGCCATCACTCCATTCTCTTCAAATGTCCTAGGGTTGCTTCATACAGCAGACCATCGTACCCAAAGCCACTATGCATATGACTTGGTTATCTCCTGCCAGGCTGTAGTCCAGTTAGTGCTCCCTTGGAAATACTCAAGACCTTAATGAACTATCTATCTGTGAATCTGTGAAGCTACCCATTTCTTAGAGGCCTCTTTGGAGAGCTGGCTGTTGAATACCCAGTATGATGGTCCTTAGCCTTCTTAGACCTTTCCTGTACCACTACCTCTATGCCTTTTGCCAAGTCCAAGTGTTCACCAGAAATGGAACTGATGCCAGAAAGACTACAGCTAACAATCTGAGCAGAACAGTGAGCATGTCCTATCTGGGAAAAGCCCAGCCCATTGGGCTAAATCTCTAGCTGAACACAGAGACTATGCTCCTTCCAATTATACTGGAAGCCCAACCATGAGAAAACAGCACTTCAACTACACTGTTCAGAGCCATCACCTGTTCACACGTGTGTCTGTATACATGTAAACACACATTTTGCAATAAAAATGCTTCTCAGATGTACTGCACCATAATAAATCAGCATTGTATGTACAGCTCAAAAAGAAGTATGGCATACCTATGGAAGAAGTCAAAGGCAAAGTGTAGAAAAATACATACAGTATGCTACCATTTGTATAAAAAGGAGCCGTATCTACTAAGTACATGCACACACAGACACACGTATATTTGTTTATATAAAATACGTCTGGAAAGATGCAATAGGAAAGTGTTGAGTGGTAACCTTTAGGGAAGGAAACTTGGGAATTAGGGTTGGGTGGGGAGAGTGTTATTTTTCACCCCTTTTGTACTCTGAATTGGTTTTACCATGTGTGTAAAGACTGTTGTATGATTTTTGTAGCACAACCCTTGAATACATAAAGCACGATTGGCTCCCCAGAAGGCAGGTGTTGGCCAGGCAGTGGATGCACGGTAGGGCAGTGTGCAGCCCTGCATGCACGTGGCTGGTATGCCCTGCGGGCAGCAGCTCCCCGCACACAGTGCTGTGAGTCTCTGGTTCATGTCACTCTGTGGAGCAGGGGCTGCTCCAGCACAAAGAGGTTTTTGACAGGGAAAGGCAGAGCCAGAGTCAGGGCAGGCCCTGGGCGGGCTCCTAGAGAGCCCAACCACCCTCTAATAAGGGGTGCTCTCCACCCTATCTGAGGAGCAATTGGGATCTCCTAGGAAGTGAAACTGTTACGTATGTCTTATTACAGAATTTCCAGTGGAGTGATTCTGGAAGAAATGTGTATGGCATGTTGTGACTGCTATGTCTCATGAAATATTTACGTTTTTATGTAACTTTTTTCCATTTTCAGATCATCTTTTATTTTAACGCAAAATGACAATATATTTTTCAATTAATTTGCCCAATATCTGTGTTTAAATGTTTTCTAATGCACACACGTGTTTTAAACTCATTAAAAAAATATGACTTAGATCCCATCCCACTTTCATGTTTTATATTGCCAAAACTCTGCTTTTGGGAGAGGTGAGTTATCTTCACTTCTGAAACAGTACTGCTTGGGAGCTGGTTTGGCCCTGGCTATTCTGGGGAAATGACACAATATTGACCTAGAGGGGCCCTAATCTTTCCCCGCTAGAGCACTACCTCTAATAGGGTGAAGGATGGGAGAAAGAGGCTAGTGAACCCACCTTCAGGTCTGTCCACTCAGGACATGCCCACACTAGCCTCTCCAGGGGCTCTGCCCTTGGCATCCCCGTCCCCCACCTGGGCTCTGGCAGAGAAGTCAGGCGGGAAGAAGCCGGCTGAGTGCCCGCCACTGTAAGAGGAAACGAAGAGAAACAGCTCCAGTCAGCCCCAGAAGCGAGGCCTGGAAAAACTCTGCAGCTGGAGCTAACACAAAGGACTTTTCAGACCCTGCAGCCAAGGTGCCCAGTGAGCTTTTTTTTTTTTTTTTTTTTTTTTTTTTAAAAAACAGCTATTAGGGTGACAAGCCTTTCCAAAGGCCAGCAGTTGGCGAGAGTTTCCAGGAAGCTGGTCTGGCACTGGGCTGGGATTGGAGCAACTTTAACGAGTTAAGTTCAGAAGGAAGGAGTCATCAGGAAAGGTTTAAGGTTCAACAAACTTTTCAAATTATTTTGCAAAAAGTTCTGGCTTTCCAATTCAAAGGCATGTTTCGCTGTGACTGTTTTAAGCCAGAGCCTATTTAAAAGGCAACACAACTAGCATGCTTCAGATTCACTCACTCGAGGTATTTGTCTAAATCCTCCGAATGGGCTTTGCAAGTGCCATCTAGTGCCTAGTAACACTGGGGCCATCTTACACATCTCGACAGGCAGCCCTCCTCCCCACGTGCCTCCCCCCAGCCTCTGTGGTCGTGTGCGCGCGGGCGCGCACACACACACGCACGCATGCACCTTCCCTAAGCAGGAAGCTGCTCTTTGCCAAGAGCGAATGGAATTCAGATTTCCAGAGGGTGTCCTTGGCCTCCAGCTCTACGGCTGCATGATGAAGAAAGGTCCTAGTGTGTGTGCCGGGGGCTGGGAGAGAGGGGCCTCCAGCTCTGCTCACATCTATCTGGGCCCCAAGAAAAACCCACAACTGGCTGTGCTCTCCCCAGCCACACCCAGCCTTGGTCACATTCCTTCCCTTCCTGAGCCCACACTGGGTGGCCCCGGCAAACCAGCTTCCACACCTCCCTCCCCACCGGCAGGGAGACCTAGCCCAAGCCCCCATGGGGCAGCCAGCAAGTACAAATGAGGGGCTGCACCCCAGGCCCAAAGAGCCAGAAAGCACGGAGGGCCCTCCCCCCACCCCACCCCCAATGCTGGGCATCCCCTTGTGGCTGCTGTCACTCATGGGGAAAGAGACACACTCCCTTGTCTAGCACCCGGTGAGCTGAGCTCCCATCCCTTCCTGTGCCTGCTTTCAGGAAGTCCCTCTTCATATCCAACCTTGACTCTCCTGGCTGTGGTGCTTGTGGGCCTGGGATTGAAGGGCCATCACTGCCCTTTGCACCAGAGCTTCCCAACATACCAGCAAGAAGGGACTGTACCCCTTCCCCTGTCCAGTCTGGGCCTTCCAAGCCCTGAGATTCAGCAACCTCAGGAATTAAACATTTATAGGAAAACACCTATGCAGACAAGGGACATGAGTCAGGGTGGTCATCAGCAGTAGCAAGGTGGGTAGGCTGGTCACGTTTAGCTTCTCTTTAGCCCAAGTAATAGGGTCTTCCACCTGCATGGCCAGGGAGGGGATAGGCTGAGAGCAATCTTTCTACAGGCTGTCAGCATCAACAAGTGGTTGTGATGAGCAGGAGTCTGTATGGCTTTTCAGGTGTTCCGGCTCAAGGAGGCCCAACACCAAGTTGGAACCTCCAGGCCTGGTGCCTAGGCAGAAGGTGGGCTAAACAAAGGCCAAGGGGAGCTCTGCTCTGTCACTTCCCTTTGTTTGCTAATGAGACTGTTTCCAAAAGCTGGCCTGGTAGCCTGTGGGATTGTTTCTCTCAAGATTTCTTCTAGGGGAGAAGGTGGCTGAGGCTTTGCAGTCTGCATCACCCAGTCCCCAGAATCTACAGGGGACTCAGCTTGCCTAGTCACAGTTCGGCTTTTGGCCTTGGCTCCAGGGCCCATTCTGATCCTCGTGCTTGGGTCAGGGATGGTCAGCACCCCTGCCCTGATGCTGCCTGCTGGGGTCTCCTGCTGAGTACCGTGAAGGCCTACTGTGGCCTGAGAAGCCAACTTCACTGTCCCTTTTGGCCAGCCCTAGACCCTATATTTGTTCAAGTAACACATTGATTCACTGGCTTCTCCTTTAGGCAAGGCCTGCAGGGGACTCAGCTGGGGTTCATGATTCCTTGCCACCTGTGGTGGGCCTGCATGGCCAGCCCAAGGCCTCAAGTGCCACATACTGCTCCCCTGACAGTCAGGTGGCTAGGGTATGACACTCAAGAAAATGCCCAGGCAGAACCCAGAGTTGGGGTCACTTGGTCCAGCCCTTCTGAAGGGGACTGGTTGGATCTGTTGCGGGGAGGAGACAGGCCATGCTGGACCAGCAAAAAAGCATAGAACTTGGAGTCTGGAACCTGACATCTGGCCTTGGCTCTGCCATCAGCTGGCTGTGTCCACTGGGCAAGGTCCTATCATATCTGGTCTCAGTGCTTCCATTTACCCAGAGTCCTTCCATCACCTGACACCTACAGGGATGCTTTCCCAATCAAAATTCAGGCCATGTGGTTTGATAAGGCCACCTTCCAATGTGGAAGACAGGCTGGTAATGGAGAGGCCTGGGAGCACAGAGCTCCTAGGCACTGCCATGGAATGTGGGGACCTTAGGGAAGGATATCTCAATTGAAGCTCTTCAGAGCCCCTGGGGTGGACTGTTCTGTACTCTCATCCCCTAACCCATACCAGCCTGAGCTCAATCATATCAGCCTGTGAAGGCTGCCCCACCACACTGCTTCTGCACCCACCACACCAGGCCCCCTTGAGCTCCCACCTGGAGCCCACACACCCAGCTGGTGGGGTTCGTGGGGCAGTTCAAAAAGAAAAGCAGAAAAAAGCATTTGAGTATTTTGGACTGGAAGGAATTCAATTTAACTTAACAAATCAGGACATGAACCTAAAGAAAACACTGAAAAACAAGCCCAACTTTGAAAGGCCCAAAGAGGGAGGATTAGGTGGGAGAAGAAAGGTCCCCTGTGGGCAGAAGACAGCTGCTCAGTCCAGACCAGGGACCAGGGGAGGATACTGGGCTTGAGTGCAGACCGGGCCAGGGTAGGGAGACAAGCCCAACATACACTCGAGGACTTGAAGTCAGGGGTGCTAGCTGACTTTTTAGCCCACTCTTTCTACCCCAGATACAGAGACTAGCTGCCTGAGGAAGCATAGCGGGGCCCACAGGCTGGCTGCCTGGTGCTGCCCACCTCCTCTCCAGGGCTTGGCTCCCTGGGGCAGAAGGCTCAGACCTATAACCACAGTGGGGTTGCAGGGATTTATTTGGGGGAAGAACCCTGACTGGGTCAGAATGATTATGTGCATGCATGTATGTGTATGTAGCTCCTTGTTCTCTGAAAAGCCTTGCCTACCACCCTACTTCCCAAGTACCCTCTATTGCCTTGCAAAGTCACCCCTGCTCTCTGCCTCAGTTTCCCCAGCAGGGCACCACAGGACACTGCTGTGGCAAGGTTTATACTTAGGGTGCAGTGCAGTCTGATCTTGTATAAATGCTCAGTGCCAAAGACTTAGGAGATAAGTCAGGCAGAGTCAAAATTAACCTTTGAAAAATCCCTTGAATTGGCCCTAAAAATAATCCCTTTCTCAGATCTTTGTGTATATCCCCTGCTGGGTTGCTCTTCTGCCTGCTAAAGTGGGAGAATCTCTGGGTGCACATCCAGAGTACCCTGGGAGAGGCATGCCAGGTGTTCCTGTACCTTTTTCAGGATATTCCCAGTGTCATCTTCAGAGGGAGCCTCTGAGGCTGCAGCCACACATGGGAAAAGATTACTTATGGAGAAGATCAGAGGCTAAGGGGCTGGGGATGGTGGGGACATCGGATTTTGAGGTCAGACCTGGACTCCTGTCTACCTCTGTCTCTCTACCTTTTATTTTTCTTTCCTTTTCTCCTAACAAGGCCCTATTTCTAAGATGGCACCTCAGGCCTCCCATGGGACCAACCAGGCCTGACAGACTAACTGCTCAGACTAACTGCCAGACTTCCTCCTGCTAGCCAGTCACTACCAAGGATGTGTGCCTCAGATACAGCCCCTGCCCAGGACACCTACGTTGTCCCTAACAGGGCCCACATATCTAAAGCATGAATGCCTAGCCAGGGGCTCTGGGGCACCATAAGAAGGCATGAAGCTGGGCCTGCCAATTTGCATCCTGGGAGGGCAGGTCAGCCTTGTCCAGGCAATCCAGGAGGCTTCCCAGTGGCCCACTGGCCAGTGAGGACCTTTGACCTCCTTGGCTGGTGGCACCAGGTTTATGATGCACATAGAGGCTGAACTTGAAGAATTCTTATTGCAGAGGGCAGCCACAAGAGCCACTGGCCAGTGCCCAGATAATATCTGCAGACTGTTCCCTCCTGCTCCATGGGCCACAGAATCCCTTTGTCTTGTTACTGCTACACTGCCTTCCTACCAATCTTCAGGGTTCATCCTGGCCTCTGCCATGCATATCCAGGCTCCCCCAGAGATTGTGTGTCTGGGAATGAGCCCTGGCTTCTGCTCCTATCTCCCAGTGATACTGGCAAGTCCCTATCCTTCTTGAGGCTTCAATTTCCCCTTCTGTGAGCTGGGAGGACAGGCCCACTAGATGCTCTCTGAGGGACCTACCAAGGATGTGGGCTGTGTGTCTCAGCCAACAGCAGTATCTCCTAACCCCAACACAAGAAATACACATAGTAGGTGTCTGGTTAATGCTCTTGGAATTTCAGCCTATACTAACCATCTGAAAGCCACTGAGTCTGAACTCTGCTACCATATTTGAGATTATCTCTCCATTGGGCACCTATGGGTTGCCTAGAGACAAGCTACCATGGTCCTCTTCCCTCGCCCGTCATGTGCAGGGTCAAAGGCAGGTGAGCTACACAGCTCGCCCTGCTCTTCACTCTGCTTTAATGTGGGTGCTTGCCTTGGCAGATGGGCCCGACAGTGATAAGAACAAATGACAGATGCATACTGGGGCACCTGTGACTGCCATGAGACACAGTCCACTGATTCTGCCTCCAACCCCCTCCCCAGAGCAGAATAGTGAGTGCTAGGTTTCCGCCTGTACCTCCACGGGACCCCAACAGCTCCTGAACCCCATTCCGAGATTTGAGGTTGTGACTTGGGGACCTCTAGACTTCCCTTCCCTCCACCTGACTCCAGACTGTCCTGGAGCTTGGGACTCTCAGGCCTCTAGGCACATCTTATTAAGGACAGCAGCTGACAGCCATATGTTGTGACCCCATTTGTATCCCATGGTAACTCATGACCTGGCAGTCTCAAAAGCTCTCAGGCCAAATAAGGAATGTCCCCACAGGATCTCCCATCACAAAGAGACATTCTATGCTGGCTAATATTTTCAGGCATTTCCCCGAGTCAGGTATGTGGCTGGATGCCTAACACCATCTCCTCAAGGCCTTACACTAATACTGTGAGATATACTCCCAATGTATACAAGAGGAAACTGAGGCTGATAGAGATTAGGTGACTGCCCCAGTGTCCTGTGGGCAGGATGAAATGACCCAAGCTGGCCAGGGCTCCCTGACAGACCTTGTCCCTGGATTCAGCGAACATACATCAAACCATTAGCTGACAAGGAATGAATCTGACAAGCTGTTCATGACCACCACACTGAAAACTACAGGGCAAGGTAAAAGATATGAAAGTCAACCTAAATAAACAAGGGACACATGATGCCATGTTTCAGGAGCCCCAAAACTATAAGGATACCCATTCTCCCATGCCCATCTAGAGGCTGCTGCGCCTAGAGGAGGCTTTCCATGGCAACTGGCATATGTATGGTGAGGAGAAACCCTGCAAAGACAGCAAAGCCAGTGATGGGGAAGGAGAAGCAGCCAGAGCCAGCAGGACCAGCTGTCAAAACCTGTTAGCAAACTACAGTGAGAAGGAAGCAAGGTCCTGGCCACAAAGAACAACTTGAATCTGACCAGTGGCACCCAATAGGGTCCAGAAACAGAGCCACACGTAGCCAGTGAGCTGATTGTAAAGACACTGATACGGCAGGGTAGTTGGGAGAGGACGGGCCTTCCCATGCCGGGTGCTGAGCTAGACTGGATATCCAAGTAGGGGAAGAGTGGACCTTGACCTCGGCCTCACATTGGACACAAAAATCAATCCCAGATGGAGTGCAGATCTACATGGGAAAGGTGAGCTAATAAAGCTTTAGAATAAACCAGAATATCTTTATGACCTTGGAGTAGTCAAAGTTTCTTAAATAGGACACAAACAGCCCTATCCAGAGAGGAAATGTTTGATTAACTGGACCACATGAAAATTAAGACCTTCTGTTCATCAAAAGACACCATCAAGAGAGGAAGAAGGCAGCCTGCAGAGGGGGAGGCGATATTTGCAATGTGTGTGTCTGTGAGAGAGTGTGAGTGTGTGTATCTCTCTCTCTGACAAATGACTCCAGAATATCTAAAGATATATAGGACCATAAATAAACATAAAACAGGCAGAAGATGTAGGCAATTTATACAGAATGATGTATCTGACTAAATGATAACATGGAAAGGGACTCAACTTCATTAGTCAACAGGTTACTGCAAATTAAAATCACCATTTGATGCTGCTAGACATTCACCATCATGGCTGAAAAGGAAACGACGGAAAATGCCAAGTGTTAGGATGTGGAGCAGCCAGAATGCTGCCTGTGGGAATGAAAATTGGAATGACTCCTTTGGTAGACTGCTGGGTAGTATTCACTAAGAGTAAACATATGCCCACCCAGGACCCTGCAGCTCCACACATAGGCATATATTCCCCAGAAACGCAACCTGATGCTCACCAAAAGCATGTACAGAATATTCTTAAACAGCAACCTACACCAGAAACTGCCTGCTGGCCACAGGTGGTAGAATGGATGAATGATGTAGGCCATGCACACAATGGAGCACCAGTCAGCAAGGAGCATGAGGGAGCACTCTCCCTTCAGCCCCATAGAGAGGCTGCCAGACATCAAATTGAGTGAGGGACATTGGACACAAAGGTGTATGAGTTGCATCATTCCCACCGCATAAAGTTCAAATGTGGGCAAAGCTGGTCCTTGCTGCTAGCAGTTAGGCCTGTGGTTAATCTCAAGGTCAGTGACAGGAAATGGGAGCCTGAGAGGCTTTCTGGGGGTTCTGTTGCTTCCTCCGGGTGCTGCACACATGGGTCCCAGGGACTGAAGATGCCTCAAGCTGCGCACTTGAGACCTGTGTACTAATCTGTGTGCATGCACATTACCCTTTAACTAAAAGTTTAACACCACCACCCTATACCAGGGTAGCCCAGGTGAGCCTAATTAGGTGGATAGCCCTGGCCTTCAGGGCCGCTAGGCCCAGCCCCATCCCACTCCCTCACTTAAGGGGACACGGCTTCCAGACTTCCTGACTAGGTCACCTCCGTGCTTCAGCCTCCAGAGGCTCTGCAGTTCAATCTCCCCTTTCAGGTATCAGCAGAGCACACAAGAGATCTATATGGGATGTGCCCCCCGCCCAACCCCGCCCCGACCCCCTGCCCCAACTGCCAGAAGTGTCTCCATGACAATATCAGTGCAGTTGTGAAGTCCTTTGCCCTCCAGCCACCTCTGTGGCGTGAATGTTCTCTCTGTCACATCCTCCAACACCATCTGCTTGGCTGAGGACCTGAGCAGCATCATGGCTGGGAGTTCACAAGTGGAGAGGGCTGAGCCTGGCCAGAGTGCACTGGGGCAGGGTGATTTGGATGGCCTTTCCCTTGCAGAGGAAATGAGAAGCCTCACTGCTTTCTTCTGGGCCATCCACCTAGACAGTGCGGCCCTCTCCCCATAGCAGCAGCCCTAGGCCAGCCGCTTCTTCCTCACGTGCTCAGACGGAGGAAGTG |
| LINC00086 | Cytosol | TGTGCGGGATCGTGGAGGTGGGGCCGAGGCAGCGGCCGCCTGAGCCCCGCTCGGCCTTGGGAACACGGGGGCGGGGCGGCCGCGGCTCTGGGCGACCCGCTGGGTGCACTAGTGCTCGGGTCCCACCGCCCTGAGGCTCGCGCTCGAGCGGGTCAGTCGGTCGGCGGGGCCTGCGCGGGGCCCGGGCCCATGGCGGCGTCGGCGGCTCTGTCTGCAGCGGCGGCTGCGGCGGCCCTGTCTGGCCTGGCGGTGCGGCTGTCGCGCTCAGCTGCGGCCCGAGGCTCGTACGGCGCCTTCTGCAAGGGGCTCACGCGCACGCTGCTCACCTTCTTCGACCTGGCCTGGCGGCTGCGCATGAACTTCCCCTACTTCTACATCGTGGCCTCGGTGATGCTTAACGTCCGCCTGCAGGTGCGGATCGAGTGAGCGCCGGCGGCGGCGGCGGCGAAGGCCCGGCTGAAGGGGCGCCCGTGTCCCCGCCCGCCCCCGGCCGGGTCGCCGGCATGAAGGAAAGCTGGGCCGCGGCGGGGGGCGGAGGCGGGGCGGCTCGGACCCCTGGACTCTAGACCTACGCCGCCAGGGCACGACGGCCCAGCCCTGGCCCCGGCTGCGGTCTCAGCCCGGGGGCCCTGGATCGCGCAGAAACGCACTGAATGGGCCCCTGCCATCGGGCTCCAGAAACTACCTGGGCTCGGCCGACCTGTTGCCTCATATTGGCCAAAGAGGGGGAAACCAGAAGGAGGGAATTCTGCTGCGGCGACTTGACTTTCCAGGCCCGGAGCAGAAAGGCATTGGCATTTGTAGGCGGTGACCCGCCCCTTCTCTGGCCTTGCCAAGAGTCACATCCCTGCCCAGGGGCACCTCTGGCCCTGGAACTTGCCTGGGCAGAGGCAGCGTGAAGGGCCTGAACAAGAGGAGAAGAAGGGCCTTCCTAGTAGAGGCACAGCATGGACAAAGGCTCACAGGGGTGGGGGTGCCCAGTGATCGAGTCCTGGCTTGGGAAGGAAGGTCTGAGTTCCCTGGGAACTGAAATCTGCTAGCAGCACTGTGAGAGAGGTGTATTTCCCCCTCCTAATGACAGAGGAAACTGAGGCTTCAGGGAGGGGTGGATTTGCCCTTGACATGCAGATAGGAGGAGGAGGAACTGCGTGTGCCCCTGGGCCTGCAGGCCCCCACACCCCTCCCCAGTCTTCTTCAAGACCTGGCATGGTGGGAGGAGGGAGGGGGAAGTGGAGAGGGGAGCATAGGGCTCCTGGGGCACCAAGGGAGAGAGGGGCCCAGGGGTAGGGAATCTAGGAATGTCGCTTTCCTTGGAGCAGTACGGAAAGTCACAGGGAAGATCAGGAGGACGGACAGCTGAGATGGGAGACAGGAGAGATGAGCCCCAGGACCCCTGGGGAGCCAAGCTGTCCCCCACATCCTAGCCTCTCACCCCACCTGGAGCTTCACCAAGGGCTCCTCAGCAGTGAAGTGGCACAAGCCTCCCAGTTTGGTGGGCAAGTGGGGGTGATCTTGGTGTTGTGGCTCCTGGAGACACGACATAACCAGGAGGGTGAAGGGATAAACCTGGGGTGGGCTGGGGCTGAGACCCATGGCATGACCCCAATTCTCTCTCCTCAAGCTCGACCCCCCATCCCCAGGATCACACAGGAGAATCTCATCCTCACGGCTTGGATTGCTCCTGGGGGCCCCCTGGTGTGCTGCTAACTGGTGTACAATGCTCAGGAGCAGCCCAGAGGGGAGCCGGGAAGGGACCCTCGCCCTTACCATCTATCCCCATTTCCGCATCTCTTGCACTGGTACCCCGGGCGCCACGTTCTCAGTTCCTGGGACTGAAAACTGCAGCAGTCTGGCCAGCTCCAGGGACAGAGTGGCCCAGCCACCTACTATGTACCCTCCTCAGCTGCCCACTGGACTCAGGTCCTGAATGAAGCTGTCCGCCTGCCTCATGCCAGAAGCGGCTGGACAGTGGCTGCCTCATGACCCTTGCAGTCTCCCACATCCAGGGCCTGATGACATGCCCCCTTGTCCCAAGTTTCTTGGGAACCCCTGACCCTGCTGGCCCCTCTCATCCACCCCAACCCTATCCACCCTGGACCACCTCTGGGGGTCTGTCAGCCTGCTGGTCCCCCCAACAGATCTTTGGGGGCAGCCTCTATGGGACAAGAGTGACACAGGGCTGGAGAAGAGGAGTGAGGAGCCTCCTTGTGTCTGATGCACAGATGTGGCCCTTTCAAACCCTGGTGTCACCCTCTGGGTGACTGGATCCCCAGCTCCAGCCTCTTCCTGGGCCAGCCAGGAAGGCTGGAGGAAAGCTCTTTGCTGAGTGCATGCATGGGAGTGTGGGGGGTGACTGAGCCCTCCCCATGCAAGGGGCTTGGCCTGGGACCCTGGAAGCTGTTTCCCTACTGGGATAAAGTTGCGTCAGCCGCAGGGGTCTCTGCCCTCAAAGACCCCCCACTGCAGGGAGCCCCACCCCATAAGAGGGTCACGGAAGTCCATGTCCGCCCACCCCCAGTGGCTTCTGGTGTGGCCGTATTGGCCTAGAGGGGCTGACTGGGGAGGGTCAGGGCCAAGCCCTCAGCATCTGCTCCTGTCCCTGCTTTTTCACCCCTGCTGCCTGAAGTGGTAGCCCCGCCTGCTGCTTCTCCACCTCCCCTCCCCACCTCTTCTCTCCCAGATGGGGCCCTTGCTGCGTGACGGGGTCTCCATGCGCTTTATTTATTTGCAGTCTGTTTTCTAGGCGGTGGAGCTAGACACTGACCGGAATGACATACTTTTCTGTGTGTGATTCACTGTGTACTGGTCAGCACAGGCTGGCCAGAGAGCTGTTCTTGTTTCTGGTGTTGTCACGTCTTCTTGTTTTCTCTAAGTTTAAAAAAAAGTCCTTGGTTTAATACACTAAAATCCCAACTGGAGGCCTCCCGTGTCTGGTGGGGGTGATGCAGTGGCCCCGGGTGGAGAGGCCCCATGGTGAGGTCACCAGCTCCCATGGCCAGGAGTTGGCAGGGAGAAGCCTCTGAGGGCTAGGGCTGGGGGAGGCTGTGGCAGGGACCGTGTTTCCTGTTCAGAGGCTGTGCTGAGAACCGACTTGACCTGGACCACTGCCCCATGAGAAGCAGGCAGCATCCTAGCCTGGCCCCGAGAGGTAGCTGCTTGTTCTGTTGACAACCTTTGTTCAACGACCCAAAACATAAGTCCCTTTCCTTTTTAGAGTCTGGCGATCAAAAGTGTCTTAAGTCAGTAGAATCCTGAGGGTGGACTAGGGTGAAGTGAACTGAAACAGACTCGACACAGGTCATTTTGTGTAGCACTGAGACTCCTCTCCCTGCCTGAGGCCACCTCCCACTGCCTCCCTACCAGCAAGGTTACTGCAGAATCTTGGAGAAGATAAAGGACCTGGCCCTGGAAGCCTGCTTCCCTTTCCCAACCTTGGAAGCAAATTCTGTCCCATCAGAGCCCCCCTCCCTAGTGGCTCCCACCAGAGACCTCCCTAGTGGCTGCTGTAAGGGTTGGCAAGCAGGCAAAAGGCGGGGGAGGGAGGGCAGAGGGTAGTGTTAGAGGAAGCCCCATAGGGCCCCTGGGTGGGGGTGGGGAGGCATCCTGAGGACACAGGGCTATGCTCAGACTTAGGAGTGAAGACTGGGTTTAGGGGCCCTGGGGGCACTCCTGTTGCCAAGGGCTACCCAGGGGTCAGAGCATCCTGACAAGGAGGACCCGTCCAAACGCACTGGTGCTAATGCCCTATGGCATCTGCCACCACAGGAGAAATGGACCCCAGTCCTTGATCTTCCCTCCCCTTTGTCTAGATGGTCCTCTTTATCCCCACTCCAGGGATCACATGACTTGGCAAAGCAAGATGGGAAATGATCCTCCCAGAGCCTGGCTCCAAGGCTTCTTGAAGAGGGAGGATCACCCCTCCCATCCCTGCTACAGAGGAAAAGGCAGGAAGGCCGGTTCTGCTCCGATGGGACTCCAGGAGACCAAAGCCCTGAGCCCTGAGCCCAGCTCTTCCACTTACTACTGTGTGGTGCCAGGCAAGCTGTGCACAACCTGGAGTTTTTAGTAAGGTTCCGTTGGGATGCTTGTAAGCAGTGAATACGATGATGTCTGTTCATTGTTTGTCATCAGGTCTCACCTGGAGCAGCCTTGTTACTGTGGGGAAAAGGAACCAAGTGAAGGGAATCGGCCTCTGATGAGAGCCTGCCATGTGCCAGGCACCATACTAGGTGCTTTGCATTCCTAATCTCATCATTATCACACAACCACCTAACCATTATCCCTGTTTTACAGAGAAACCAAAGACAGGAACCAAGTCTGAAACAGAGCCCCAAGAAGAAGCTCTTTTCCTGAGCTACCAGTGCCCCTTGGAGAGGCAGGGAATTGGGCTGGAAGAATGGTGGAATGGAATAGCAAAGATGGAAGATGACCCTGTCCTTTCTGGGTGAGGTCCAGAAGAAATCCAGGGCTTGGATGAGGGGTAGACCTGAGAGCCTTCTCCAAATGGAACTCCCCAGTGGGAGCAGCTGTGGCCACCCTGAGATGGGTCTACCTGTCTTCCTTCCATTCCACCTCCAGAGATTGATTGGGACCTAAGAGACTGAGTCAGGAGAGGGCAAGAAAGCAGCTAAGTGGGAAGTTTCAGGGGCACTAGAGCAAATACATTGAGAAATGGGAAATACAAACAGCATGCAAAATCACATCCTACTCCTACCCACCCCACTCCCAAGGGAAATTGCTGGGTTTTGGACGAATTTTCACTTTGTGTGCACCTTTTATGTATGTAAAACTACAAACATACTACTTTGAAATCTGATTCCGCTATTTCATGTTTGTCTTACCATGTTATTATGTATTTTCCTACATCCAAAGCTACCTAGTATCTCTTTTGTATGAAGGTTTCATCATGTATCCCCCAGTGCCCTTTGGTTGTTTTTCTTTGTTGTCATTTATTTTGGTGGGGGTTGGTTTGCTTTTTGTTGTTGTTTTTCCTTGTTTTTATAAATGATACAGTGACAGATGCCGTTGTGGATAAATGCTGTGGCATAGTCATCAATTCCTTGATATATATTCCTAGAAAGTGCACCAAAGGGTATGCATGGCTTAGGGCTTTTGATACTCTCCAGAGTTGGGATACCAAGTCACACTACTCACTGGAGTCAGGATGAGAGTTTTTTGAGACAATGGTGGCAATTCTGATTCAATAGATTAAAAAATTTATTACTACCTT |
| LINC00087 | Exosome | CATGGCGGCGTCGGCGGCTCTGTCTGCGGCGGCGGCGGCGGCAGCCCTGTCTGGCTTGGCGGTTCGGCTGTCGCGCTCGGCGGCGGCCCGAGGCTCATACGGCGCCTTCTGCAAGGGGCTCACGCGCACGCTGCTCACCTTCTTCGACCTGGCCTGGCGGCTGCGCATGAACTTCCCCTACTTCTACATCGTGGCCTCGGTGATGCTCAACGTCCGCCTGCAAGTGCGGATCGAGTGAGCGCCGGCGGCGGCGGCGGCCGCGGAGGCCCGGCTGGAGGGGCGACAGTGTCCCCGCCCGCCCCCGGCCGGGTCGCGGGCATGAAGGACAGCTGGATCGCGGCGGGGGGCGGAGGTGGGGCGGCCCGGGCCCCTGGACTCTAGACCTACGCCGCCCGGGCACGAAGGCCCAGCCCTGGCCCTGGCCGCGGTCTCAGCCCGGGACCCCGGATCGCGCAGAAATGCACTGAACAGGCCCCTACAATTGGGCTCCAGAAACTACCTGAGCTCGGACTACCTGTTGCCTCACATTGGCAAAAGAGGGGGAAACCAGAAGGAGGGAATTCTGCTGCGGCGACTTGACTTTCCCCGCCCCGAGCAGAAAGGCATTGACGTTTGTAGGCGGTGACCCGCCCCTTCTCTGGCCTTGCCAAGAGTCTCATCCCTACCCTGGGGCACCTCTGACCCTGGACCTGCTTGGGCAGAGGCAGCGTGAAGGGCCTGAACAAGAGGAGAAGAAGGGCCTTCCTAGTAGAGGCACAGCATGGACAAAGGCTCACAGGGGTGGGGGTGCCCAGTGATCGAGTCCTGGCTTGGGAGGGAAGGTCTGAGTTCCCTGGGAACTGAAATCGGCTAGCAGCACTGTGAGAGAGGTGTATTTCCCCCTCCTAATGACAGAGGAAACCGAGGCTTCGGGGAGGGGGGGATTTGCCCTTGACATGCAGATAGGATGAGGAGGAACTGCGTGTGCCCCTGGGCCTGCAGGCTCCCACACCCCTCCCCAGTCTTCTCCAAGACCTGGCATGATGGGAGGAGGGAGGGGAAAGTGAAGAGGGAAGCATAGGGCTCCTAGGGCACCAAGGGAGAGGGGCCCAAGGGTAGGGAATCTGGGGATCTCGCTTTCTTTGGAGCAGTACAGAAGATCACAGGAAAGATTAGGACAGACAGCTGAGATGGCAGACAGGAGAGATGGGCCCCAGGATCCCTGGGGAGCCAAGCTTTCCCCCACAGCCTAGCCTCCCCACCCCACCTGGAGCTTCACCAAGGGCTTTTCAGCAGTGAAGTGGCACAAACCTCCCAGTTTGGTGGGCAAGTGGGGCTGATGGTGGTGTCATGGCTCCTGGAGACACGACATAACCAGGAGGGTGAAGGGATAAACCTGGGGTGGGCTGGGGCTGAGACCCATGGCATGACCCCAATTCTCTCTCCTCAAGCTCGACCCCCCCGCCATCCCCAGGATCACACAGGAGAATCTCATCCTCACGGCTTGGATTGGTCCTGGGGGCCCCCGGTTGTGCTGCTAACTGGTGTACAATGCTCAAGAGCAGCCCAGAGGGGAGCCAGGAAGGGACCCTCGCCCTCACCTGCTATCCCCATTTCCGCATCTCTTGCACTGGTACCCTGAGGGCCACATTCTCAGTTCCTGGGATTGAAAACTGCAGCAGTCTGGCCAGCTCCAGGGACAGAGTGATCCAACCACCTACCACGTACCCTCCTCAGCAGGCCACTGGACCCAGGTCCTGAATGAAGCTGTCCGCCTGCCTCACCCCAGAAGAGGCTGGACAGTGGCTGCCTCGTGCCCCCTGCAGTCTCCCACAGCCAGGGCCCGATGGGGTGCCTCCTTGTCCCAAGTCTCCTGAGAACCCCTGACCTTGCTGGCCTCTCTCATCCGCCCCAACCCTGTCCACCCTTGACCACCTCTGGGGGCCTGTCAGCCTGCTGGTCCCCCCAACAGATCTCTGGGGGCAGCCTCTGTGGGACAAGAGTGATACAGAGCTGGAGGAGAAGAGGAGTGAGGGGCCTCCTTGTGTCTGATGCACAGATGTGGCCCTTTCAAACCCTGGTGTCACCCTCTGGGTGACTGGATCCCTAGCTCCAGCCTCTTCCTGGGCCAGCCAGGAAGGGTGGAGGAAAGTTCTTTGCTGATTGCATGTGTGATACAGTGGGGGGTGCCTGAGCCCTCCCCATGCAAGGGGCTCATCCTGGGACTCTGGAAGCTGCTTCCCTACTGGGAGAAATGTGTGTCGGAGCTGCAGGGGTCCCTACCCTCAGAGACCCCCGACTGCAGGGAACCCACCCCATAAGAGGGTCACGGATGTCCATGTCCGCCCACCCCCCGTGGCTTCTGCTGTGGCTATATTGGCCTAGAGGGGCTGACTGGGGAGGAGCAGGGCTAAGCCCTCAGCATTTGCTCCTGTCCCTGCCTTTTCACCCCTGCTGCCTGAAGTGGTAGCCCCGCCTGCTGCTTCTCTACCTCCCCTCCCCACCTCTTCTCTCCCCTACAGGGCCCTTGCTGCGTGATGGGGTCTCCATGCACTTTATTTATTTGCAGTCTGTTTTCTAGGCGGTGGAGCTTCTAGACACCGACCGGAATGACATACGTTTCTGTGTGTGATTCACTGTGTACTGGTCAGCACAGGCTGGCCAGAGAGATGTTCTTGTTTCTGGTGTTGTCACGTCTTCTTGTTTTCTCTAAGTTT |
| LINC00114 | Cytoplasm | TTTCCTGAGGCCTCCTCCGGAGTGGAGCAGATGCTGCCATGCTTCCTGTGCAGCCTGTGGAACGGGTCTCATTCTGTCTCCCAGGCTGGAGTGCAATGGCACAATCATGGCTCACTGCAGCTTCAACCTCCTGGGCTCAAGCAATCCTCCCACCTCAGCCTCCAGAGCAACTGGGACTACAGGGACTTTAAGAAGCTGCTGAAGAACCCAAGAGGAAGGTGGGAGCTGTTGATGGTGTGGAATTAGAGGCCTGATGGAGTGGAGAGCTTTCCTCCTCATCACCTGGGTCAGGACCATGGAGCTCCTTGAGCCTTGAGATTCCAGGCCCAGGAGCACATCATCATTGTGCTTCTTGGGGCCCAGGCGCATCTCCTAAGAACTTGTTCTTAGACATGTTGTGCTCAAACTTGTGGCATCATAGCAGTAATAGTTTACAATATACCTACAGTTTCCTGGGCAGATCATCGTGTGCAGACCGGCTGCAGAGAGAAGGGAACTGTTTCACTTTCATCTTCCTGGGCCATTTCCAGATATGAATTGCAGAGACATATTTCAATATATCCTCACCCAAATATGCTTTAGAAGTACATTTCTTTTAATGTAATAAGATTTATGTCCTTTGTGTTTGTTTAGAGGGTAAATCTGGATTTGTATTTTTAGAAGTTACTTTCACCAAAGAAATTCGAAAGAAATAGTGTTTCAGAAAAATCAAAACAAGCCATAGAAATCTAATATGTTTCAGATACTATATTGGGCTGCATGAAGACTAAATCCATAATATGTATGAATAATGTATTCTAAAGTAAATATCATATTTTGTCAAATTA |
| LINC00462 | Cytoplasm | CTTCTTCCTTTAGGTTAACTCCCATGAAGAGCTTTGAAAGAAATAGAAGGATATTACATCCTCTTGGATCAGTCTTGCAAATGGAGAACCTGCTCTCTGGGAGAAAAGTCAAATCGGTCCCACGGCTCTCTTCTCCCACTAAGAACAACTGGTTCCTTCCTTCAGAAGAAGATGTGTTATTCCCACCAGTAGTGGTGAGCGCCTGGATTCCAAAAGGGTTAAGAATTCGGAGATGTGACCTGCCTCATTCTGAAGAACGTGGCCCAGCATGTGTATGAAGCAATTAATTGCTCCACAAACCCTGAAAATTTGATCACTGGCCAGAGTAACGCTGAGGAGCCATCCTGTCTTCTGCTTGAGTGTCTTCACTCTGGGGATTCTGGCAGGCTGGTTTCCTGGCCAGATGCTTCTCATCCACACCTGTGGACCTCAGGAAACCAAAGGTGACCTCTGTTGGCATGGCCTTGACTTCCTTTCCACCAATGCCCATGAGATGTGGAAATTGAGCGAACATCCACACCAGAACCATACACTCAAACAAGCAGGTTGGGGCAGGGAAACTCCATCACATCATGGTGAGGCAGAGATGCTGCAGCCACCCAGATCATTGTGTAGTAGAGGTGGCACCATTTTACTATGTGTCCTCCCTGCACTCAGTGAAGTCTGTGAAGGGAGGGGTCAGTGCAGCAAGGAGCTCGCTCTGCCCCAGAGGAGAGAGTGGAATGTTCGAGGACACATGAAAGCAGTTACTCTGCCTACCTTTTAAAACTCTTTCCTCCTGGGGAACTGGTTTATTCTGTACTCCTAAACAGACTATATCCATGTATTCACTCCTTCTATAAATATTTGAGAAAAACCTACTATATGCCAAGCACTTTTCTAGGCAAAGGAAATGCAATAGCCCTTTCTTCCCATAGCTTCCAGCCTGGTGGGGAATAAAGGAATTAAATAAGCAATTACAA |
| LINC00486 | Cytoplasm | TCCACGTTGGCTGGTTTGAAACTGTGGTGTATGTTTAAGCTCAGGCTGGAAAAGGATCACTGAAAGTCACTTGGATGGTGTCTCGTGTGTCTGTGATGAGCTTGCTGTTGCTTTCTTTCTCCTTGACAGATGAGAAAACAGTGGCTTCAAAGGTTAAACAACTTGTCTTCGATTGCACAGCTAACAAGAGCTCACTGTCCAAAGGGAGTCTCTCTCCCCGGTCCACATGCTGAGGCTTGCTCCAGAGTGAATGTGTGCACTGGAAAGAACCCACACAGGCCCCCTGAGAGCTGTCAGCCTTGCCAGGCTTCTGTGAGATCCCTGGGTCCGAGTAGCCAAGCTGGGAGGAGCACCATTTGCCCACTGACTCTTGGGCAACGCTGCAGCCTGCATCCCAGCCGGCAGAAGGCAGGTGGTATCACAGCTGAGTTTACAGCCACGGAAGGAACCACTGTAGGCTTTTAGTAGATAATATGTTTTCTCTGCTTCAAATCCTTATTTTGGTTGCTTAAAATTTCTGCAGAAAACAAACAGCTTCTGCCAAAATTGGCTCACAAAATTAGCTTACTAGTATAATCAGTTGTATAGCAAGTATTTACTGAGTAGCTCTTGGAAGGCAGTCAACACAAGAGGGATGGATCATTCTTCCAGTTCATCACACGTGGGTTGGACATGCTAACAGTCCCCGATAGCTATCAGGCGAGTAAATGGATGATCACACAAAGGCAGAGGCATCGCAGAAGGATGCTAACTAGATGTCATGCGTCAAGGATACTACAGAAGGATCTCTTTGCTGACAAGGAGGGTACCTCTAAGGTTCCTTCAGATGGGATGGTTCTCTGTTCACCGTGATCACTACTATGCAAAGGCCTGTGGGCCATTTAGGAGAAAAGCATTCAGTCCAATTCCACAGACACAGTGAGGATGTAATATGCCCTAGGCATATATGAGTGGATCCAAGGAGGAAATACTCAGTCACTACTGTCAAGAACTCATGACCTGCTGCAGGGTTTGCCAAACTATGGCTTGTGGGCCAATCTGGCTCACCACCTATTTTAGTAAATAATGTTTTACTGGAATCCAGACACGCTTGTTTGATTACATATTGTCTATGGCTGCTGAGCACCCACTATAAGGGCAAAGTTAAGTAGATAGAGACTTTATCACCCACAAAACCTAAAATATTTGACTGGATGGCCCTCTACAGAAAAAGCCTGCGAACCTGTGGTCTTGTGGGAGGACACTGCTGGGCACAGTTAACTCTAATTCTTGGCTGTAGCATCTTGAATAGGGTGGCAGTCCCTGGCTGCTGAGGGGGACAGCTGGACGGCTGCCTCCCAGGGGCATTTCCATGACTTGC |
| LINC00501 | Cytoplasm | AAGTCCAGTGCTAACCTGTGGGTGAATGAACAATGACCGGGGAACAGGGCCCTAAGGTCCTGGCTAATGCTGGGAAAACACCAAGCTGAAGAAGACATGTTTCCTGCCCTCCAGCAAAGAGATAGAGTCCAAGCAAATAACTACAACAGATGTGAACGCGGAAGCACAAAGGAAGAAGCTAATGCTTCCTGGGAGAGGTGACATTTCTTCTGGGTTTCGATACTTCTTCTAGTCCTCTCACATCTTAAATCTCCATTCTCAGTATTCTCAGTGGAGCATTTTATTCTGCTTCTATGATTTGGGGCAAAAAGAAGAGAATCTATTTGATAAAGTACTTCTAGAAAGACTGGCAGAAAAGACTCTGAAAGACTTTCTCAGGAGTAAGAAACAACACAACTTTCTTAATATTCCTGTGCTGTTTTCACATGCAAAAAATACTTTCCCCCTCCTACCTCATCGGTAGGGCAATGGACAAAATGGCTTTTCTTATAGTAAATAGCATGTTCCATGTAAGACGAGGCCATGATCTCCCTTTTTTGCACTTCAAAATATTTTAAAATTGCTGGATTATTTTTTCCTCTCCATTCTTCCTTCTGTGGCTTGACAATTACTAAAGCTGGTATGATTGATAACACGACTCAAGTTAGAGGAAGTCTTTTTTTTCTTGAGATGGAGTCTGGCTCTGTCTCCCAGGTTGGAGTGCAGCGACTGCGAACTCGGCTCACTGCAAACTAGAGGAGGCCTTTTTTTTTTTAAATCAAAGAGCCTCTTTTAAAATTATAACCCATTGGGAATATGCTGTGGGGAGGTCTCTCACAGCAGTGTGAAAGAGCACATTATCTTTTTTTTTTTTTTTTTGAGACGGAGTCTCGCTCTGTCACCCAGGCTGGAGTGCAGTGACGCGATCTGGGCTCACTGCAAGCTCTGCCTCCCGGGTTCACGCCATTCTCCTGCCTCAGCCTCCCAAGTAGCTGGGACTACAGGCGCCCGCCACCACGCCAGGCTAATTTTTTGTATTTTTAGTCGAGACGGGGTTTCACCGTGTTAGCCAGGATGATCTCGATCTTCTGACCTCGTGATCCGCCCGTCTCAGCCTCCCAAAGTGCTGGAATTACAGGCGTGAGCCACTGCACCCGGCAAGAGCACATTATCTTGTATGTGCCTGTAGCCCATCTGCAGACATGAAACCCAAGTCCCTGTTCTCCCAAGTGCAATGGAAACATCTCCTCTCCTGCGGATGAACTGAATAAGGGATAGTGAAGCATATTTTTAAATGTTTGCTATTGCTCGTTAGCCACAGTAGGAATTTCAATACCAGTGGCAGTGGGATCCCTCATTTTATTGTAGATGCCTTTTTTCATTTCAGCTTACAAAGCTTCCCTCCGTTGGAGGGTGGGGGCCGCAGAAATAAAATGGAAGGCAAAGGGGGGATGCGCTGCGATTTTTCTGCTCATTGTCTCATTTTGAAACTGCCCCAACTGTATAGAACTTCTAGGAAGCCCGTACATGTCGGCACATTCCCGGGGAAGAATGCTTCTCTTAGGAGCCAGTGCCTTAAGAAGTTTCTTCCGTGTTGCACTGGCATGCATGTCTGCTTAAATCGCCACTCTCCTCATGACATTTGGCAGGACTAGGAAGTTCACGTGTCAGTCCCGCTATTGGCCCACGTGACCCCATCCCCAAAGACAGGCAGTGAACCCCTGTGTTTTTATGCCATATGAACTGAGAAGTAGACGCATTCTGTGTAATGGATACACACTGAGGCTGCAATTTTTGAAAAGTCATTGTTTTGACTTCCCTATATTCACATCGAATAACGCTTTTAGTCTTTGCCCTCAGTACTCTAGAAGGGGCTATATAGCCTGTTTTTGTATTTGTGGGAAAAAGACTAGAAGCTGGGCAAAATGCCACCAGGAAGACTAACATGGCGACGTGATCCCAAGGGAAACTGAGGTCTCACACTGTCCAGCAACAGAAGGAGGTTGAATTCTGGAGGAGGTTTAATTTTTTAAAAACTCAAACTAATAAAAGATGTTCACAAATATT |
| LMO7-AS1 | Cytoplasm | GCGGCCGCCCGGCCCGCTTGACATACCAGAAATAGCTGCACACAATTGTACCTTTCCCGGCCAAGAAACCTCCCCCGCTGGGGAGCGCGGCCCGCCGCCCTCACCTGAGCCCCGGCCAGCTGAGCCCGCTACCCGCTCTCCCGCCGCGATCCGGCCCAGCCGCCAGGCATATTCTACCAGTGCAAGAGATCAACAATGATTACAAAATTGCTAAAGAAGTCACTTTCAAGAGATCTGGAGAAGTAAGATGGCCAAATAAAAGCCTCTACCAATCATCCTCCCCACAGGAACACCAAATTTAAGAACTATCTACACAAAAAAGCACCTTCATAAGAACCAAAAATCAGAGAGAACAAGGATAAAGAAGTATCCAAATACAAAGAAAATGTTATGCAAGTGACCTTTAGAGATGTTTTAAAGATGACAAAATATTGATGAAGATGGGCCAACAAGTGTTACTGTTACCT |
| LSAMP-AS1 | Cytoplasm | GAACAGCTCCGGTCTACAGCTCCCAGCGTGAGCGACGCAGAAGACGGTGATTTCTGCATTTCCATCTGAGGTGGAATGACTGTGGCTCTGTGCCATCTCGAAATCTACTGGTCTCTAAGCACAAACGAACATTCTCTTAAACTAATGAAATGAGTGGCAGGCAGCGTGTGCAGAGCACCAGCAAAGGCTCAGAGGGATGCCCACAGAGTTCCACCGCAACCGTGCACAGAGGAGGAGGAGAATGCTCCCTACAGGTGAAAACTAGGCTAATACACAATGATTGAAGACTAAAGGATGAGAAGAGCCACCAGTGGGGCGCATTCACAGAGAAACGGTCACATGATGAGGCAGCAAGAAGGCAAAAATTGCGAGCCAACAAGAGAGGCCTCAGAAGAATCCAACCCTGGTGCCGCCTTCATTTTAAACTTCCAACAACCCCAGAACTGTGGAAAAAAATTCTATTTTTTGTCTGTGGTATTTTGTTATGGCAACCCTAGCAAACTAATACACTGAGCAAACTAATACATTAAACAGAATATTAAAACGAACAAAA |
| MIR205HG | Cytoplasm | TGAAGCAAACTCAAAGCTAGGTCCTGATGTCTCAAGGCACAGGTACTCGTACTTAAAGACACTGTCATCTCTCAAGTACCCATCTTGGAGGGTACGGACCCCACATGAGGAAAGGACCAACCACACCGAGCTCAGTTATGGCACACACAGTGGGACCTAGACAAAGGGAGAGGGTGACCGACATCCCAACTAGATTTCAGTGGAGTGAAGTTCAGGAGGCATGGAGCTGACAACCATGAGGCCTCGGCAGCCACCGCCACCACCGCCGCCGCCACCACCGTAGCAGCAGCAGCAGCAGCAGCAGCAGCAGCAGCAGCAGCAAGAGTAACTCTGACTTAGGAATAGAGACAGCCAGAGAGAAATGTGATCAATGAAGGAGACATCTGGAGTGTGCGTGCTTCTTCAGAGGGACGGGTGATGGGCAGATTGGAAAAAGCACCGCAGATGGGAACCTTAATCTTTCTTTTCTAAAATTGATGCTATGAAAATTTGCGTTTTCTGTAACTTGTAAAAACTAAAAGTTGCTTGTCTACTGAAAA |
| MRVI1-AS1 | Cytoplasm | AAAAAATTGTAACCACCTTGGCAGATTGTGGTGTAAGCACAAAAAGAGTTATCATTTGCAAAGAACGCTGAGCTGCCAACACCATGCTGAGGGCAGTGGGACTTCCCAGAGTAGTAGAATACATGCAGCCTGGTGTATATTGAGTTTTTGAGCCTATCACTCAGATTCTATTCTGGAAATTTCCTCTCCTTGTTGACCCTGCCCCCATTTGGTCACCCTCCCTGCCCTAGGACTGAGTACGAAGGAAAGGTTATTCTACTATTCTGTGCTGGGAGGCAGACATCGTCATACTTACCAAGGCTGCTTATGAGATCTTGGAGATACAGAATGGAAATGACCCCACAGTCTCTGGGTAACACCCTGGAGACTGTATTCAAGCTGCCTGTAGCAATATGAAGGCTTATGATCACTTCATGTTCATTTAAGGGATGTATACATAGTTAAGGAAACCATGAGCAAGAAAGGAGCCCTGGTATTCCTTGAACATGCATACCTTTCCCAGCGTGAGGGACATGGACCGTGCCGTGCGAGGGACCCCATCTTCTGCAGCAGGAGGGAGCAGAGAAGAGAACACAGGTTCAGGGTACATGTGCAGGTGCATTACATAAACTTGTCATGAAGATTTGTTGTACAGATTATTTCGTCACCCAGCCACCCTCATCAGCCAGAGTTCAAACCCTGACAGTGCCCATGGAGAGATCTGGTCCAAGGCACCAACCTCTTCCTGCTGGACTGAGAAAGAGCCTCCTACTGTGTCTCTCTTTACCTCAGCTTTTGCACACCTCCAATATATTGTCCACTGAAGTCAGAATAATCTAAAAATAAAGGC |
| PART1 | Cytoplasm | ACACATTAATCTACTTATCTGGAATCACTTTGCCTCTAAAGGCCAGAGAAAAATCACAGCTTCCTTGTCGGAGGGGAAAAGGACAGGTGATCTGGGGAAAACGCAGCTACACCTGGAGCAAGGTCTCTTCCCGGCTTGGCAATCTCAGCTGTGCCGGCGCTACGGGACCCGAGCCGTCCCAGAAACCAAAGGGCAGGCACGGCAGCAAACGCCTGAGGACTGAGAAGACGTCAAGGTCCAGACACTCGTCATTAACACCTACTCACAACCGATTCCCCCACCCACTTTGGCCTCACCAAGATTTGGCAAGCAGGAGAATTAAATGTCACTTTGCTACCATCCTCTGCTGAACCTCCAACAGAAAAACCCATATTGCCCAAGTACAAAGCATGAAATACTGCTGGCCTACCCTGACTTTTCAGCTATTCTCCTTGCATATAGTAAATGACTCCCCAGGAGACATCATCTTAAAGTCTGAGACACAAAAAAGGACCCAAAGTCCTATATGCTTGAAGAATTGCATGGCAATATATTTAGTTAACATTTTCTATTTTATTAGAAAAAACAGACAATTCAATTGTCGATGTGATCGTTTCAGGCTTCCCAGCCTCTCCTTTCTGACATCATCCTATATACTACAACCTCCACCAGAAAAGCAGTATGGTACTGGGAAAAAGAGATCTAAAGGCAGAACACTAGGGCTTCACTCTCAGCACTGTTAACTGAGTGACTTGACTAAGTCACTGAAATTCACTGAAACTGCAGTTTCCCCTTCTGTCAAAGGGGGATAACAATATCAGCTTCACAGGGTGCTGTGAAGGAAGCAAATGAGAAAATGAAAGTGAGCATCCTTTTAAATTATAAACAGAGAATAAGGGAGAGGGGAGGGGGAAGGGGAGAGGAGGAAGAAAGGAAACAATCTGCCCTCCCTCACCCCTTCACACTAGGTAAAATAAAATGTTTAGACAGAAAAACCTAAAGAAAAGGATCCAAATAAAATATTCTTAGGAACTGTCAAAGGGAGAAGACATTGGAATCTCCAAATCTGGCCAAGAGCTCAGCCTAAGCAGCATGTTTTGAACTCCTGAGTCCTTGGAACCCTGCACTAAAGCTTTGGGACTCTGACCTGCAGGAAAAAAAAAAAAAAAATGCTTCCCTCAGTTCATTCCCTGTAGTTTAAAAACAAGAGCAACTAAAGGGCAAGCTTCGGGGGCAAGGGGAGACAAACACCTCAGACCATTAACTTACTTTATCCCCATTGGAAATCAACTTCTGGAGAACATTTCAGCTTTGTATGCTCTTCTTGGTCCCACTCAAATGGGAAGTTCTGCTCGAGGCCAGAAATAGAAAACAGGAAGTCAACAGAACTGATTTTCCTAACTGCCCAAATGCAAATCACCACACTCCGTTATGGATGAGAATTAGAGTAAAATGTAAACTTCTTTTTAAAAAGAAGTTCACATGCTAACTGTGTACAGAAAACATCCTACTCCAGGGAAGAAAGAAAGAAAGAAAGAAAGGAAAGATCCATCAGCTTCCTTCCTTAATAAAGGAGGATTGACAGAGCTGGACTGACAGTGAAGTAAAGGTTACCGACGGTGTGCTCATCAGAACCATGCTGCCACTAAAAATAATGAAGTGTCCCACTTTGATGCGACACTATTCTCCAAGTTGGTTCCTCTAAAGGTGCTTTTCTTCAAGGGAAGCAGATACTAAAGTGACAACTTTCTCATTACCATTCAGACTATTTTCTTAGAGTCCCAGGTTTGAAACTTTCAAGTTGAAACTCTAAGCTGCACCCACTGCATACAGACTTCTTAGTCAGTTTCTGTCACGTACCATCATATCTACCTTATTTGCATCAGTCATAAATTCTGCTCATTCTTCTATCACAGCTCCTCTGACATTTGCCAGACCTCACCATTTCCTCAGCCATTTCTGGTCTGAATCATCAGCCCCCCATGCCTGCTGCCTGCTCTCTTCTATAAACATACCAGATGAATCTTTCCAGCATGCTGCTTCCCCTGCCCCCAAGTCAGCCACAGTTCCCTACTACCTATTGATCACATCTGAGTTGCCTCAGCCAGTGGTCTTGCCATATCTACCTAGCTTAGGCTGAGTGTTATAGGAGAAAGTGCACTAGCCCGGGCACCAGATGGATCAGGTAAGACTCTAGCTTAGGTAAGCTTTATCACATCATATAGTGGCTGTGTGGATTGAGGAAAATTATGAAACTTCTTTGCACTTCAGTTTTCTCATTTGCAGAATGAAGATAATAACACCTACCTATACAGTTACTAAGGATTAAATAGGATAATATGCACAAAGTGACCCTAGTGAGTTTCAGTAAGAAGAATTAATGCTTATGAAGTGCTTATCACATTCCAGCTTCTGTTCTGTGTGCTGTGCATGTGTTTATGCAGGTATTATTATTAACCTCATTTTGCAGAAGAGGAACTTGAGGCAAGAGAGAGCGTGTAAAGTCACATAACAAATCCACAGAAGAGCTAGCCTTTGAACTCCAACAGCCTGACTCCAGTGCCTGCATATGTAACTCCATGCTCTACTGCCTCCCATTTCCCTTTCCCTCCAACTTTCACCGGCTAACACAGCCCACAGGTCCACCTCAGTCCTTCTCATCCTTCCAGTCTTCCCTCCACATTCCAACTACCTGTGACAAATGCTATCCTGCATTTTCTCTCCAATCTTACCTGACATCTTGAAGGGAGCAGTTCAGTTACAGCATTCTCTCCATACCCAACTATACCATCTCTCTACTCTTTATCTTGCCCTCACATACACATAAGTTTGTCATTGTTCTTCAGTTCTTTGGGTAATCTCTTCCTAGAAACTGCATCTAACAAATAGACCACATCCCCAAATTCCATCATGTCCAAAAAGAAGCACGGTATTTCCATCAACACCCCAGCTGCCATCACCAAGCCAACTCGCCTGACTTCCATTCACTTACTAGCAAACATTTACCAAGTATCTGCCATGTTCATTTTGTCAATGAGAATGACATGGGACTTGTTCCTCTAGGAGATCACAGTCTAATGAGATCAATGTTTATATAGAAAATGTAAATAGGTTGTGATAAGTACTAAATGCACGCTGCTAATGGCATCATCACCTCTGGCTCTTCTCAATCCTGCGTTTCCCATGTTGTGTCATGCAACAGAGCTGTCACACTGCCTCTTCCTCTTCATCTCTGGTGCCACCTGTGATTTACTTGCACACTACCTTGTTTCTGAACTATTGCAGCCCCTCCCAACTTATCTCACTGCCTCATTCACCTGCCTTTCAAAACTGGCCCACCTCCTGCTGCCCAATAACCTGATGTTGTCACTCTCATATTTAAAACCCATAAGTGCTGCCGGGAGCGGTGGCTCACGCCTGTAATCCCAGCACTTTGGGAGGCCGAGGCGGGCGGATCACAAGGTCAAGAGATCGAGACCATCCTGGCCAACGTGGTGAAGCCCAGCCTCTACTAAAAATACAAAAATTAGCTGGGCGGGGTGGCACACACCTGTAGTCCCAGCTACTTGGCAGGCTGAGGCAGGAGAATTGCTTGAACCCGGAGGCAGAGGTTGCAGTGAGCCGAGATTGCACTACTGCACTCCAGACTGGTGACGGAGCGAGACGCTGTCAAAAAAACAAAACAAAACAAAAAACATATCAATGCTGATGAACCAAGTGCAAAGCCCTGAGCTTGGCCAGGCAACATAGCCTGGAAACCTGACTTTTAATCCTTACTTTGCTCTATTCCCCCAACATACACACACTTCATACACCACACCTAAAAGAGACTTCCCACAGTTTTCCAAAAATGACTCACATTTATATTTCCTGTTTTTCCCTTTGCCCACCCTGCCCTCTCTCCCTCTGCCTTCATTTTTTTAATTAACCTCTGGAACTCAGGTGCTCCTGCTCCAAGGTTTAGGTCAAAAGTTACCTCCTCCATGAAGCTTTCTCTCATTCTCCCACAATGTGTGAAAGCCCCATTCTCAGATTTGCTTAGTTGTGCATTTGTATATTCTACCTTGCACTACAGTCATTTGTATATTTATCTTATGGTTTCTGTGCCTTAGGAAAGGTACACTGTCTTAATCATCTTTGTTCAATACAGTGCCTAGCAGAGTGCCTCAAGTATAATATGTGCTCAATAAATAATAGATGCATTTTTCCATGCATATTTTCTACTATATCGAAGTGGAAAATATATCCATGTGATATAGTGAAAAATATCTCATGAAGAGAGATACAGTGAGGAAAATATCTCATGAAGACCAAAAAGCAGAAATCCAAGTTTTTTCTTTTCATTACTATCATCAGTGATTATTAATTTTATGTGTCAACTTGGTTGGGCTTAGGGATGCTCAGATAGCTGGTAAAATATTATTTCTGGATGTGTCTCTCAGGGTGTTTCTGGGGGAAATTAGCATTTGAATCAGTAGACTAAATAAAGAAAATCTGCCCTCACAAATGTGAGCCAGCATCATCCAATTTATTGACAGTTTAGATACAACAATAAGGCAGAAGAAGGTGAACTCTCTTTCCTTGAGCTGGGACATCCATCTTCTCCTGCTCTGACATTGGAGTTTCTAGTTCTTGGGACTTCAGATTCTCCCCTGAAGTTTCTCAGGCCTTTTGGCATCAGACTGGAAGTTATGCCATTGGCTCCCCTGGTTCTCAGACCTTTGGACTCAGACTGAATTATACCACTGGCTTTCCTGGTTCTCCAGCTTCAAGATGACATATTGTGAGACTTTTCAACCTCCATAATTGCATGAGCCAATTTCCACAATATATCTCCTCTTATGTATCTATATATTTCCTATTGGTTTTGTTTCTCTACAGAATCCTAATAGATCATCTAATTGTTTATCTAACAAGTATTTTCCTCCCTAGACAATATGTTGGGCACTAGGGACACAAAAATAAAAACACAGACATCTGCACTCAATAAACTTACATTCAATATCAGAGAGATTGGCACAGAATACAAATAAAATGAGATACATGCAAAATAATATGAATTGCAAAATTACGGCAATTCACCACCAACTAGGTGTGTGACCGTGGGAAAATCACACTGTGAGCTAAATATTAGCAAGTTTAAAATGCAAAATCAGAGGCAGTCTATGAATTGGACTTTGCTAATGGCCTCCTTTGCTGATGGATGGCACATTGGTGTTAGAAAGTGCTTATTTTTGCAAAGGTCCATTATTGATGCTTTAATTCTAAGGAAATTATGTAGGCAGAAACTAAAGAAAGGTTAAAAAAACAGCAGCTAAAATTGATACTTAGACAATAAAAAACTAATTATAAGTGATTTATCAGCACCACTAGATAACTACATTGGTGAAATTTAAGGCCATGAAATGCAGGACAAGCCTCGTAGGAAGCAAGGTCCCAGTGGATGAGATGCTGTCCTCTGGGCTCCTAACCTTCCACCACTAGTATTCACCATATCTGATTGGAAATGTGTGCATGTGAGTCGGCCTCCCCTCTATGATGTGAGTAGGGACCTATTTTTGCTCATCTCTGTATCCTCTACACACGATGCTTGGCACACAGCAAGTATTCAATAAATGTTTATGAATGAAGCAATCA |
| RBMS3-AS3 | Cytoplasm | AGAAAGAGAAACTTCCATTGCTAACATGAAGCCAAATTGTCCTAAACAGGAGTCTTGAGACTTCTCTTCTTTTGGTGTGGATTCCGGAACTCCTTTCTGAAGCAAGAGGCAAATATCTCTCAGCAGAGAATGCCAAGACCAATCACTTAACCATGGCAATCTTACTCAGCATGGTAATTCCCACCCTGATGGTCCCAAGATGGCTGCAGCAGTTTCAGCCATGTTATCCTAATCCACCAACATCTGGAGGTATCTGTCTTTTCAATCTCCATCTATTACAAATGTAGGATACTTTTACCAGAAGGTCCTCCTCTAGACTACTACTCATGTTTCATTGGACAAATTTGGTCCCATGGAATTTCTTAAGTCATTTACTGATGAGGAAAGTGGAATTATCATGACTGGATTAGACTACCTAAGATTCACCACTCCTGAGCCTGGAGAAAGATAATTTTCTCAGAGCAAACAACTGCATATGGCTGAAGATAGGAATGAACTATAAAACAAAATTGGAGTCCTCCAGGAAAGAAAGGGGGAATTAAAGGGAAATGATTGTGTAGGCAAAACACAATAGTCTCCAATGCCAAATAAACTTTGTTTTGTTACTCTCTCA |
| SHANK2-AS1 | Cytosol | AATGACAGAAGCAGCAGCGAGAGCCCTTCAGAGGACCGCGAAACGCGAACTCCCACCCCAGCAAACCACCAAGGGGAAGAGGCGGGCCCCCATCCAGGAGGCCTTAGGAACAGGAGAGCTGACTTTTCTAAAGCAAATAAGGCAGTGTTCTCCCCAGGGCGCCAGGCTTCCCCAGCTCCAAGTCTGGGCGTCTGAAATCTGCTGTGTAATTGGATCACGCCCGGCCCGTGCTGAGCGCCCATCTGCGCCCGTCTTGGAAAACACAAGTCTCAGAGAAGGAAAGCAGTTGCCACAGTGTTCAGCTTAACCAAGTCATCTTATTTGATATGTGAAAGTTCTTCGAGATGGAAGAATCTCAGCTCATCACTGCGAAGGCATGCCAGGATTAGGAAGTCATCAGATGCAAGCCTGCAAGCCTCAGTGTACTCCTCAATGTCTGCAGGGCCCACCCCAGAGAGAGGCCCCTGGGTGACCAGCCGGTGAGGAGCTTCAAGAGGAAGGATTCACCATCACCACCTGGATCCTGTGTGCAGCTTTTGTCACAGCGGTGGCGAGCACAGTTTCTGCCTCCTGGGGAGATGGGGTGTGAAGCTCACAGCACCTGCGTGTGGCTGGGCCCAACATTAACAGCGCTGCAAGAGCTCACCTCCATTTATAGGAAGTGGGAAGGACCCAAGGACTGGCCGATGACACCACAGGAGGCAGAAGGCACATCGGGGGTTGGGGGTAGCCTATGGCCAACCTACCCTGTCCCTCTAACAACCTACCCTGTCCCTATAACACGTTGCTGGCAGGAAACACGGGAGATGGCTCAGACTGGAGACCTGGGGACATATCTACCAAGTGCCACGTGGGTCCTGGGTCAAAGTCACCACCGTGAAAAGACATTTCTGAGATAATTTGGGACATGTGCTAATGGAGGGGTTTCAGATTCCACCCAGAGACCACTGCTGGGTGCGTGGATGGGACAGTGACACTGGGGCCCGCAAGAAAGATCCCATGCTGTGAGAGACAGACTGAAGGGGAAGAGGGAGTGACGCGATGCCTGGGATTTGCTGAAGAAAAAAAAAAGAATAAAAAGGTCCGTCTGTATATGGATGGCAAGAATTTCTTGGTAACAAAAGCACAGCCAATAAAAGAAAAAATAAATACTAAGGTCGATGAAAATTTAAAACTTTTGTGCATCAAAGGGCACCATCAACAGAGCAAAAAGGCAGCTAAAGAATGGGAGGAAATATTTGCAAATCATGTATCTGGTAAGGGGTTAGTATCCAGAATGTGCAGAAAACTCTTACAACTCAACAACAGAAACAAAAATCTAATTTAAAAATGGGCAAACTGGCCAGGTGAGGTGGCTCATGCCTGTAATCCCAGCACTTTGGGAGGCCGAGGTGGGCGGATCACTTGAGGTCAGGAGTTCGAGACCAGACTGGCCAACATGGCAAAACCCCATCTCCACTAAAATTTCAAAAATTAGCCAGGTGTGATGGCACGTCCCTGTAACCCCAGTTACTCGGGAGGCTGAGGCAGAAGAATTGCTGGAACCCAGGAGGCAAAGGTCGCAGTGAGGTGAGATCACGCCACTGCACTCTAGCCTGGGTGACAGAGTGAGACTCCGTCTCATAAATAAATAAATAAAAATGGGTAAAGGACTTGAATAGACATTTCTCCAAAGAAGAGATACAAATGGCTGATAAGGACTTTTCTGAAAAGATGTTCAACACCACTAATCAATTAGAGAAAGGCAAATCCAAACTACAACGGGACACCATCTCACACCTGTTAGGATGGCCACGATCCTAAAACAGAAAGTAATGACGCTGGTGAGTATGTGGGGAAACTGGAGCCCTTGGGCACCGCTGGCAGGAAGGTAAAGTGGTGCAGCCCCTGTGGAACATGGCATGGCTGCTCCTCAAAACCTTAAACACAGAACGAGCACGTGAGCCAGCAATGCCACTTCTGCATCTATGCCCGAAGGCATTGACAGCGGGGTCTTGAAGAGATATCTGCACACCCGCGTTCACGGTGGCAGCACTATTCATGGTAGCTAAGAGGTGGACGCAACCTAAGTGTCCACCTACAGATGAACAGACACACAAAAGGTGGTGTTTCCGTAGAGTTGGGATAGTTATCATTCAGGCTTAAACCAGAAGGAGATTCTGATGCATGCTACAACACGGAGGAACCTCGAAGGTATTACACTGAGTGAAATGTACCAAGCACAAAAAGATAAATACTGCAGGATTCCACTG |
| SOX21-AS1 | Cytosol | TCTTCTTGGCTCCGGGCAGGGTGCGCCGATGGGAAACCCCCAATCGGCTGGGTGATTTTTTTTTTTTGGAGGGGGGAGCTGTCCCCCGCAGCACCTGCCTCTCGAGTCTTCCTCTCCCGCTGCTGGACTGACGGGTGGGGCGAGGAAGGAGGGTGGATGAGGCTTGAGCAAGCGTTGGTCGCCCGGCCTCAGAGGAAGTTCTCCCGGCTCGCCGAGGAGCCGCGTCCGGCCGTCCCCTGGGCCATGCCGGCTCTGTTCGCTCCGAGCCCCTTCAGGCGCGCCTGCCGACTTAGGGCTCCCGGAGCTCGCCGGCCGCGGAGGGCAGCGCAGCCTCCACGCGGCGCCGCAGGCCTGGCACCGGCCGCGTCCCCGCCTCGCTCTTCTCGGCAGCCGCCTGGCACTTGCAACTAACTTCGCCCAAGTTTCCTGCGCCGCGGCGCCCCTCTCGGCGCGCTCCTGGGAGCGTGCGCCCGCGAGTCCGCCGCGCCCGGGGCCCCTCTGTCCTCGCTGCTGTGGCCAGAGCCGAGCCGGCGCAGGGGCGGAGGTGCTGCAGGAGAGTTAAGGAAAACGGGCCATCACCTCAGTTCGCAGGTCCGCGCTGTGGCACCTCCCGCTCTCCCCACTCCCGCGGCGCAGGGGGCCCCTTCCCAGCCCAGGCCGCCCTCTGCACCGGCTCAGGTTTAGGCGAGTGGAGAGTCCGGAGCGCGCCGGGGAGTCTGCGCCCTGTTTGGCGTTGGCAAACCCTGGTGTCATCCCGGCTACCCAGGCCGGCCTGGCGTGCAGCTGCCCTGGTTGTGGCCCTGGAATCCTCGCCGGGAGGAAGAAGGCGGCGGGCACGAGTAGGAGAGCCTCTCCAGCTACGGAGGAAGAGGGTTAAACCTGGAGAAGGGGAGACTCACCCTTGAAAATGTTTGAGACTGTTCCTAGCAAGAAAGTGCTCATTTTCCTTGGAGGTCCAGGTGTTGCTTGCAACAGAAACAGAGGCTTCTCGCATTTTCTTGCACTGGCGATGCACCTAGTCAGAGGTAGTGGAGTGAAAAATAACTCTGGAGGGAATAAGGACATTCTTGGCAACCTTTCCAAAAAAGAGCTAGCCCAGACTGAGAGTATGCATCCTTGACTGACTATTCAGCTCTTTAAAAATTCTGTGTTGTTTGTCTTTTGTTGTAATCTTGGTCGCCAGAACCATCTATATACTGATCTCTTTAAGGTACAGTTAACTTACAGTGTCTCACTTACATGCGCTGCTGAGGTGAAGGTGTTTAAAATCAATAAGTGTAAGTAGTTTCCCTGGAAGAGAGAAGTAAAACGTACCAATCACTCAGTGCTGGAATATTTTTATGCTAAGCATAATTTCCTTCTTTAGTAGTTAGAGCAGTTTTTTAAAAAAAATAGTTAACACGATGCAGAAGTCAAACAGCATGATTCTGTTTCCGTATTTTCCTCACTTTTTGGTATGCTGCGGCACATTTAGAACATGATCTGTCTGGAGGTTTTATTCACATGGTGGCCTGCCCTTCAGGGAGAACTTGCTTGCCTTCTTTCTTTGGCAGTTTGGATTTGACCTAGCCCTGTGGTATGTGCAAGTAGGAACATCTACAAACGGTTAACTCTTTAAGTCTTCCTACGATTTATGTTCAGTTCATTCACACACACACACACACACACACACACACACACACAAAAGCTTTGATTGTAATTATGAACGTGAATTAAAACTGTTTCAGGCCATCAGATTTATGTAAAAGTTAGGCTTGTTTTGTTCATTTTAATAAAAGAATAGTGACTTAAAAATTACAGTCGTGATAACAAAACTGTGAACAGCAGTGGCATCTATGTGGGTTTTTGTTTTTCTGAAGATGTCGTATTGACAAGCTTATTTTGTATCGGTTATGCCCTTGCATTGTTAAAATAATTGGACTGATTTCTCCAGAACCATATTCCTTTACAGGCTCTGTAAAGACACTCTTGTCATTCACAGACTCTGGCTGGAGCAATGGGAAAACACTAGCATGTGTAATTACATAGACTGGAATATTAAATATACTGTTTTGTGATGATAAACTACAAAATTATCGTCCCTGAGCTTTTCACCTACTGACTTATAGGTTTGAGACAAAAATACTATACACTGGAACATAAAGAACATATGTATATATAGGCCACAAAACATGAAATCTGTTATTCACTAAATATTGTGAATAAAGTTAAATTTAATCGTGAAAATTATTTTGACACATAAAAAGGGAGAAGGATTGTGAAAATGTGTTTTATATTAGTGAGTTAAGTGATTTTATACTATTCAAATTTAAGTGTAAAAATCTACTGCAATAAATTATTACATTAGAAATGTTTGTATATATGATTGAAATGTATGCTTACATGTGCAATGTCTTATAGGAATAATATATGTAAGGTTCCTTGGCTATAGTTTTTTCCAGTATACATTTACAATATTGTCTGTCTAGTGATATTAAATGAGCCAAATTTGAACACTCTCAAGTCCAAGAATGTTGAACTGTGGGAAACAAAGGTGTCATAAGAAGAAAATGACTATTTGTGTGCTTGTCTATTCACCAAATGAAACTTCCCACATTTCAAAATAGGAAAGATTTGGTTTTAGTTCTTGGCTTCTAGGGGATCAATATTCTTGGCCTAACCCTTCACAACCAAAGAAAGAGCCCTCATAATCGGCCATTCATTAAAGAAAGGCAATGTGGCATTGCTGTTATTAACCAGCCTTCACACCTCTTGCCTGAATCTGGTTGAGCTTTTAACAAAGTAAATTTTCAAGCCATTTTCATTCATATGTACCAAAATGTAAATTTGCTTTTAATTTCCTCATATCTTGTGTTTGAATTGATAGAGGTATATTTTTATTCTTGCTCAGTTTTGTCCTAAAGATTAAAAAAATCCCAGCATATAGGAAATGGAATAGTCTGTGTGAGTGAATAGTGAAGGAAAATAAATTGAAAGAGAATTGTAAGTTTGATGAGCTTTATTTTGTCAACTCTTGATTGTAAACACCTAAAGAACAGTATAGATTTGATCGGTAGAATTCGTGTGGGTCTGTATTTCCCTTGCGGCCACAGTGATATATGTGATCGCAAATGTATCTAGTCGGAAACTGAGTGACACTCCCTCAGAAAAAGTTGTGGGCCTGTGTAAATTGAGTTTCCTCTTTTCCCCCTTCAATTCTCCCCAGAAAATAGTCTCTTTTCAATTCTAAAACTTTCACTAGGAGGCAGCAATGAGATTGTTGGGGAATAAAAAATACCTTGGCAACA |
| UCA1 | Nucleus | AAGACCTGCCGCCTATAAAAAGGATTATATCTTGAGACCCTATCCTCTAAAATTTTTTCCACACCCAAAACAAAAAATCTCTGGGTCAAAAGTCTAAAACGCTTAGGCTGGCAACCATCAGATCCTTGCCCATGGTGTCCTCAAGCCTACTCTCATGAAATGGACAACAGTACACGCATATGGGGCCAGTTCCACATATTTGGCAACCAGACCAGCATCCAGGACAACACAAAGATCTGCAATCAGAACTATTGAACTTCTCCATTCAGACCGCCACTCACACCTATGGGAAAAGGGTAATGTATCATCGGCTTAGCAACAGGGAATACTATTCGTATGATGGAAAATGGGGACAAAAGGCTTTGGTACATAAAACATTATTCCTTCCTTGGCCTAAAAACTCATCGCCACCTACATTAAAGCTAATATGCCTGAT |

**Supplementary Table 4. GO functional enrichment analysis results.**

| Category | Term | Count | PValue | Genes | Fold Enrichment |
| --- | --- | --- | --- | --- | --- |
| GOTERM_MF_DIRECT | GO:0003700~transcription factor activity, sequence-specific DNA binding | 5 | 4.74E-05 | SOX11, E2F3, PROX1, AFF3, RUNX1 | 18.86381869 |
| GOTERM_CC_DIRECT | GO:0005654~nucleoplasm | 8 | 6.47E-05 | ANLN, NOVA1, SOX11, E2F3, PROX1, AFF3, EZH2, RUNX1 | 4.619785055 |
| GOTERM_CC_DIRECT | GO:0000785~chromatin | 5 | 4.18E-04 | SOX11, E2F3, PROX1, EZH2, RUNX1 | 10.76001261 |
| GOTERM_MF_DIRECT | GO:0000978~RNA polymerase II core promoter proximal region sequence-specific DNA binding | 5 | 9.58E-04 | SOX11, E2F3, PROX1, EZH2, RUNX1 | 8.658289607 |
| GOTERM_BP_DIRECT | GO:0008284~positive regulation of cell proliferation | 4 | 0.001159704 | SOX11, E2F3, PROX1, EZH2 | 15.58874722 |
| GOTERM_MF_DIRECT | GO:0000976~transcription regulatory region sequence-specific DNA binding | 3 | 0.004244731 | SOX11, PROX1, RUNX1 | 26.34593838 |
| GOTERM_BP_DIRECT | GO:0010468~regulation of gene expression | 3 | 0.004640412 | NOVA1, PROX1, AFF3 | 25.17124183 |
| GOTERM_BP_DIRECT | GO:0000122~negative regulation of transcription from RNA polymerase II promoter | 4 | 0.005845356 | SOX11, PROX1, EZH2, RUNX1 | 8.850281512 |
| GOTERM_BP_DIRECT | GO:0006355~regulation of transcription, DNA-templated | 4 | 0.006156015 | SOX11, PROX1, AFF3, EZH2 | 8.688550479 |
| GOTERM_CC_DIRECT | GO:0005634~nucleus | 7 | 0.009354847 | NOVA1, SOX11, E2F3, PROX1, AFF3, EZH2, RUNX1 | 2.685103991 |
| GOTERM_BP_DIRECT | GO:0045944~positive regulation of transcription from RNA polymerase II promoter | 4 | 0.010690895 | SOX11, E2F3, PROX1, RUNX1 | 7.125913591 |
| GOTERM_MF_DIRECT | GO:0000981~RNA polymerase II transcription factor activity, sequence-specific DNA binding | 4 | 0.013553315 | SOX11, E2F3, PROX1, RUNX1 | 6.536704022 |
| GOTERM_BP_DIRECT | GO:0060412~ventricular septum morphogenesis | 2 | 0.015271608 | SOX11, PROX1 | 115.6516517 |
| GOTERM_MF_DIRECT | GO:0001228~transcriptional activator activity, RNA polymerase II transcription regulatory region sequence-specific binding | 3 | 0.015978047 | SOX11, E2F3, RUNX1 | 13.25651868 |
| GOTERM_BP_DIRECT | GO:0042752~regulation of circadian rhythm | 2 | 0.027098199 | PROX1, EZH2 | 64.83501684 |
| GOTERM_BP_DIRECT | GO:0000082~G1/S transition of mitotic cell cycle | 2 | 0.030338755 | E2F3, EZH2 | 57.82582583 |
| GOTERM_BP_DIRECT | GO:0043433~negative regulation of sequence-specific DNA binding transcription factor activity | 2 | 0.037193594 | PROX1, EZH2 | 47.02319902 |
| GOTERM_BP_DIRECT | GO:0001822~kidney development | 2 | 0.048391508 | SOX11, PROX1 | 35.9589169 |
| GOTERM_BP_DIRECT | GO:0030182~neuron differentiation | 2 | 0.070056091 | SOX11, RUNX1 | 24.59259259 |
| GOTERM_MF_DIRECT | GO:0003714~transcription corepressor activity | 2 | 0.080392618 | EZH2, RUNX1 | 21.3276644 |
| GOTERM_MF_DIRECT | GO:0003677~DNA binding | 3 | 0.099309271 | SOX11, PROX1, RUNX1 | 4.875842405 |

**Supplementary Table 5. Survival analysis of ceRNAs network-related genes.**

| Gene_Name | Level | Label | No. | P-value |
| --- | --- | --- | --- | --- |
| DIAPH3-AS1 | high | >3 | 45 | 0.22 |
| DIAPH3-AS1 | low | <=3 | 207 | 0.22 |
| ERVMER61-1 | high | >2 | 202 | 0.31 |
| ERVMER61-1 | low | <=2 | 50 | 0.31 |
| KCNA3 | high | >38 | 214 | 0.085 |
| KCNA3 | low | <=38 | 38 | 0.085 |
| LINC00114 | high | >5 | 111 | 0.39 |
| LINC00114 | low | <=5 | 141 | 0.39 |
| LINC00462 | high | >13 | 70 | 0.28 |
| LINC00462 | low | <=13 | 182 | 0.28 |
| LINC00486 | high | >0 | 200 | 0.28 |
| LINC00486 | low | <=0 | 52 | 0.28 |
| LINC00501 | high | >5 | 150 | 0.26 |
| LINC00501 | low | <=5 | 102 | 0.26 |
| LMO7-AS1 | high | >46 | 50 | 0.022 |
| LMO7-AS1 | low | <=46 | 202 | 0.022 |
| LSAMP-AS1 | high | >0 | 202 | 0.081 |
| LSAMP-AS1 | low | <=0 | 50 | 0.081 |
| MIR205HG | high | >1 | 98 | 0.5 |
| MIR205HG | low | <=1 | 154 | 0.5 |
| MRVI1-AS1 | high | >3 | 161 | 0.036 |
| MRVI1-AS1 | low | <=3 | 91 | 0.036 |
| PART1 | high | >106 | 114 | 0.069 |
| PART1 | low | <=106 | 138 | 0.069 |
| RBMS3-AS3 | high | >27 | 77 | 0.27 |
| RBMS3-AS3 | low | <=27 | 175 | 0.27 |
| hsa-miR-216a | high | >1.128695 | 113 | 0.02 |
| hsa-miR-216a | low | <=1.128695 | 139 | 0.02 |
| hsa-miR-182 | high | >6498.941048 | 108 | 0.075 |
| hsa-miR-182 | low | <=6498.941048 | 144 | 0.075 |
| hsa-miR-96 | high | >3.767156 | 190 | 0.03 |
| hsa-miR-96 | low | <=3.767156 | 62 | 0.03 |
| hsa-miR-210 | high | >72.871761 | 158 | 0.091 |
| hsa-miR-210 | low | <=72.871761 | 94 | 0.091 |
| hsa-miR-205 | high | >1.546471 | 64 | 0.14 |
| hsa-miR-205 | low | <=1.546471 | 188 | 0.14 |
| hsa-miR-204 | high | >0.755882 | 206 | 0.12 |
| hsa-miR-204 | low | <=0.755882 | 46 | 0.12 |
| hsa-miR-21 | high | >315755.202269 | 79 | 0.14 |
| hsa-miR-21 | low | <=315755.202269 | 173 | 0.14 |
| hsa-miR-217 | high | >35.55389 | 119 | 0.0045 |
| hsa-miR-217 | low | <=35.55389 | 133 | 0.0045 |
| hsa-miR-195 | high | >74.326457 | 45 | 0.16 |
| hsa-miR-195 | low | <=74.326457 | 207 | 0.16 |
| hsa-miR-141 | high | >583.043344 | 171 | 0.086 |
| hsa-miR-141 | low | <=583.043344 | 81 | 0.086 |
| hsa-miR-503 | high | >1.85848 | 194 | 0.3 |
| hsa-miR-503 | low | <=1.85848 | 58 | 0.3 |
| hsa-miR-122 | high | >0.805772 | 19 | 0.012 |
| hsa-miR-122 | low | <=0.805772 | 233 | 0.012 |
| hsa-miR-200a | high | >3271.031242 | 28 | 0.06 |
| hsa-miR-200a | low | <=3271.031242 | 224 | 0.06 |
| hsa-miR-143 | high | >185098.229833 | 169 | 0.17 |
| hsa-miR-143 | low | <=185098.229833 | 83 | 0.17 |
| hsa-miR-183 | high | >6975.933773 | 42 | 0.15 |
| hsa-miR-183 | low | <=6975.933773 | 210 | 0.15 |
| hsa-miR-508 | high | >5.081111 | 53 | 0.017 |
| hsa-miR-508 | low | <=5.081111 | 199 | 0.017 |
| hsa-miR-363 | high | >2.34544 | 172 | 0.0079 |
| hsa-miR-363 | low | <=2.34544 | 80 | 0.0079 |
| hsa-miR-17 | high | >898.712537 | 77 | 0.014 |
| hsa-miR-17 | low | <=898.712537 | 175 | 0.014 |
| hsa-miR-372 | high | >0.332646 | 68 | 0.02 |
| hsa-miR-372 | low | <=0.332646 | 184 | 0.02 |
| hsa-miR-93 | high | >1281.523503 | 211 | 0.046 |
| hsa-miR-93 | low | <=1281.523503 | 41 | 0.046 |
| hsa-miR-145 | high | >3764.493559 | 101 | 0.003 |
| hsa-miR-145 | low | <=3764.493559 | 151 | 0.003 |
| hsa-miR-187 | high | >2.49517 | 111 | 0.00089 |
| hsa-miR-187 | low | <=2.49517 | 141 | 0.00089 |
| hsa-miR-222 | high | >64.869639 | 160 | 0.1 |
| hsa-miR-222 | low | <=64.869639 | 92 | 0.1 |
| hsa-miR-301b | high | >0.186563 | 199 | 0.062 |
| hsa-miR-301b | low | <=0.186563 | 53 | 0.062 |
| hsa-miR-100 | high | >4481.220603 | 82 | 0.0025 |
| hsa-miR-100 | low | <=4481.220603 | 170 | 0.0025 |
| AFF3 | high | >159 | 146 | 0.0022 |
| AFF3 | low | <=159 | 106 | 0.0022 |
| ANLN | high | >6679 | 35 | 0.11 |
| ANLN | low | <=6679 | 217 | 0.11 |
| E2F3 | high | >1343 | 127 | 0.11 |
| E2F3 | low | <=1343 | 125 | 0.11 |
| EZH2 | high | >1065 | 150 | 0.035 |
| EZH2 | low | <=1065 | 102 | 0.035 |
| FRMD5 | high | >1088 | 31 | 0.22 |
| FRMD5 | low | <=1088 | 221 | 0.22 |
| NOVA1 | high | >180 | 112 | 0.013 |
| NOVA1 | low | <=180 | 140 | 0.013 |
| PROX1 | high | >2428 | 42 | 0.011 |
| PROX1 | low | <=2428 | 210 | 0.011 |
| RUNX1 | high | >7918 | 106 | 0.088 |
| RUNX1 | low | <=7918 | 146 | 0.088 |
| SOX11 | high | >193 | 26 | 0.06 |
| SOX11 | low | <=193 | 226 | 0.06 |

**Supplementary Table 6. Correlations of lncRNA-mRNA pairs.**

| lnRNA | mRNA | Cor | Pvalue | miRNA interacted with mRNA | miRNA interacted with lncRNA |
| --- | --- | --- | --- | --- | --- |
| ERVMER61-1 | E2F3 | 0.346778499 | 6.06E-13 | hsa-miR-217 | hsa-mir-182, hsa-mir-205, hsa-mir-96, hsa-mir-204, hsa-mir-21 |
| KCNA3 | AFF3 | 0.552467926 | 6.72E-34 | hsa-miR-217 | hsa-mir-122, hsa-mir-141, hsa-mir-143, hsa-mir-183, hsa-mir-195, hsa-mir-200a, hsa-mir-204, hsa-mir-21, hsa-mir-210, hsa-mir-217, hsa-mir-50 |
| KCNA3 | NOVA1 | 0.382821523 | 1.18E-15 | hsa-miR-217 | hsa-mir-122, hsa-mir-141, hsa-mir-143, hsa-mir-183, hsa-mir-195, hsa-mir-200a, hsa-mir-204, hsa-mir-21, hsa-mir-210, hsa-mir-217, hsa-mir-50 |
| LINC00114 | PROX1 | 0.347177622 | 5.68E-13 | hsa-miR-217 | hsa-mir-143, hsa-mir-182, hsa-mir-204, hsa-mir-216a, hsa-mir-363, hsa-mir-508, hsa-mir-96 |
| LINC00501 | ANLN | 0.409819776 | 6.46E-18 | hsa-miR-217 | hsa-mir-183, hsa-mir-204, hsa-mir-301b, hsa-mir-363 |
| LINC00501 | E2F3 | 0.387921973 | 4.58E-16 | hsa-miR-217 | hsa-mir-183, hsa-mir-204, hsa-mir-301b, hsa-mir-363 |
| LINC00501 | EZH2 | 0.381738148 | 1.44E-15 | hsa-miR-217 | hsa-mir-183, hsa-mir-204, hsa-mir-301b, hsa-mir-363 |
| LINC00501 | FRMD5 | 0.310185508 | 1.58E-10 | hsa-miR-217 | hsa-mir-183, hsa-mir-204, hsa-mir-301b, hsa-mir-363 |
| LMO7-AS1 | ANLN | 0.391027947 | 2.55E-16 | hsa-miR-217 | hsa-mir-122, hsa-mir-145, hsa-mir-17, hsa-mir-204, hsa-mir-372, hsa-mir-93 |
| LMO7-AS1 | E2F3 | 0.506899374 | 5.91E-28 | hsa-miR-217 | hsa-mir-122, hsa-mir-145, hsa-mir-17, hsa-mir-204, hsa-mir-372, hsa-mir-93 |
| LMO7-AS1 | EZH2 | 0.30212829 | 4.89E-10 | hsa-miR-217 | hsa-mir-122, hsa-mir-145, hsa-mir-17, hsa-mir-204, hsa-mir-372, hsa-mir-93 |
| LMO7-AS1 | FRMD5 | 0.356056339 | 1.31E-13 | hsa-miR-217 | hsa-mir-122, hsa-mir-145, hsa-mir-17, hsa-mir-204, hsa-mir-372, hsa-mir-93 |
| LMO7-AS1 | PROX1 | 0.336909809 | 2.93E-12 | hsa-miR-217 | hsa-mir-122, hsa-mir-145, hsa-mir-17, hsa-mir-204, hsa-mir-372, hsa-mir-93 |
| LMO7-AS1 | RUNX1 | 0.408810834 | 7.92E-18 | hsa-miR-217 | hsa-mir-122, hsa-mir-145, hsa-mir-17, hsa-mir-204, hsa-mir-372, hsa-mir-93 |
| LMO7-AS1 | SOX11 | 0.343047252 | 1.11E-12 | hsa-miR-217 | hsa-mir-122, hsa-mir-145, hsa-mir-17, hsa-mir-204, hsa-mir-372, hsa-mir-93 |
| MRVI1-AS1 | AFF3 | 0.46544365 | 2.84E-23 | hsa-miR-217 | hsa-mir-183, hsa-mir-205, hsa-mir-222 |
| MRVI1-AS1 | NOVA1 | 0.482433925 | 4.09E-25 | hsa-miR-217 | hsa-mir-183, hsa-mir-205, hsa-mir-222 |
| PART1 | AFF3 | 0.452809505 | 5.73E-22 | hsa-miR-217 | hsa-mir-100, hsa-mir-122, hsa-mir-141, hsa-mir-143, hsa-mir-145, hsa-mir-187, hsa-mir-195, hsa-mir-200a, hsa-mir-204, hsa-mir-205, hsa-mir-21, hsa-mir-301b, hsa-mir-363, hsa-mir-508 |
| PART1 | NOVA1 | 0.587443924 | 4.02E-39 | hsa-miR-217 | hsa-mir-100, hsa-mir-122, hsa-mir-141, hsa-mir-143, hsa-mir-145, hsa-mir-187, hsa-mir-195, hsa-mir-200a, hsa-mir-204, hsa-mir-205, hsa-mir-21, hsa-mir-301b, hsa-mir-363, hsa-mir-508 |
| RBMS3-AS3 | AFF3 | 0.654327091 | 4.28E-51 | hsa-miR-217 | hsa-mir-204, hsa-mir-96, hsa-mir-182 |
| RBMS3-AS3 | NOVA1 | 0.694762073 | 6.15E-60 | hsa-miR-217 | hsa-mir-204, hsa-mir-96, hsa-mir-182 |

**Supplementary Table 7. Correlations of lncRNA-miRNA pairs**

| lnRNA | miRNA | Cor | Pvalue |
| --- | --- | --- | --- |
| MRVI1-AS1 | hsa-miR-141 | -0.391435285 | 9.93E-10 |
| PART1 | hsa-miR-141 | -0.492968011 | 2.65E-15 |
| RBMS3-AS3 | hsa-miR-141 | -0.484980904 | 8.56E-15 |
| MRVI1-AS1 | hsa-miR-17 | -0.310944924 | 1.77E-06 |
| PART1 | hsa-miR-17 | -0.523145673 | 2.39E-17 |
| RBMS3-AS3 | hsa-miR-17 | -0.495340882 | 1.86E-15 |
| MRVI1-AS1 | hsa-miR-182 | -0.422434634 | 3.07E-11 |
| PART1 | hsa-miR-182 | -0.452746636 | 7.16E-13 |
| RBMS3-AS3 | hsa-miR-182 | -0.410430707 | 1.23E-10 |
| MRVI1-AS1 | hsa-miR-183 | -0.376922951 | 4.48E-09 |
| PART1 | hsa-miR-183 | -0.450470913 | 9.62E-13 |
| RBMS3-AS3 | hsa-miR-183 | -0.436460836 | 5.65E-12 |
| LINC00501 | hsa-miR-195 | -0.347190037 | 7.87E-08 |
| MRVI1-AS1 | hsa-miR-200a | -0.309295764 | 2.02E-06 |
| PART1 | hsa-miR-200a | -0.456385118 | 4.45E-13 |
| RBMS3-AS3 | hsa-miR-200a | -0.45304436 | 6.89E-13 |
| LINC00501 | hsa-miR-204 | -0.346866254 | 8.11E-08 |
| LMO7-AS1 | hsa-miR-204 | -0.311223675 | 1.73E-06 |
| PART1 | hsa-miR-21 | -0.392639646 | 8.73E-10 |
| PART1 | hsa-miR-210 | -0.472827248 | 4.80E-14 |
| RBMS3-AS3 | hsa-miR-210 | -0.492242203 | 2.96E-15 |
| MRVI1-AS1 | hsa-miR-222 | -0.313256641 | 1.47E-06 |
| PART1 | hsa-miR-222 | -0.545876242 | 4.99E-19 |
| RBMS3-AS3 | hsa-miR-222 | -0.528730739 | 9.49E-18 |
| KCNA3 | hsa-miR-301b | -0.315019601 | 1.27E-06 |
| PART1 | hsa-miR-301b | -0.336635649 | 2.03E-07 |
| RBMS3-AS3 | hsa-miR-301b | -0.334467586 | 2.46E-07 |
| PART1 | hsa-miR-503 | -0.478193416 | 2.26E-14 |
| RBMS3-AS3 | hsa-miR-503 | -0.396175284 | 5.97E-10 |
| PART1 | hsa-miR-93 | -0.477364595 | 2.54E-14 |
| RBMS3-AS3 | hsa-miR-93 | -0.467364502 | 1.02E-13 |
| MRVI1-AS1 | hsa-miR-96 | -0.453380607 | 6.59E-13 |
| PART1 | hsa-miR-96 | -0.458186821 | 3.50E-13 |
| RBMS3-AS3 | hsa-miR-96 | -0.481590188 | 1.39E-14 |

**Supplementary Table 8. Correlations of mRNA-miRNA pairs**

| mRNA | miRNA | Cor | Pvalue |
| --- | --- | --- | --- |
| AFF3 | hsa-miR-141 | -0.514631142 | 9.47E-17 |
| AFF3 | hsa-miR-17 | -0.550261348 | 2.29E-19 |
| AFF3 | hsa-miR-182 | -0.497935287 | 1.26E-15 |
| AFF3 | hsa-miR-183 | -0.548338459 | 3.22E-19 |
| AFF3 | hsa-miR-200a | -0.4596691 | 2.88E-13 |
| AFF3 | hsa-miR-21 | -0.488093283 | 5.44E-15 |
| AFF3 | hsa-miR-210 | -0.55482813 | 1.00E-19 |
| AFF3 | hsa-miR-222 | -0.5622253 | 2.56E-20 |
| AFF3 | hsa-miR-301b | -0.416557319 | 6.10E-11 |
| AFF3 | hsa-miR-503 | -0.432801929 | 8.86E-12 |
| AFF3 | hsa-miR-93 | -0.587584105 | 1.82E-22 |
| AFF3 | hsa-miR-96 | -0.527450275 | 1.17E-17 |
| ANLN | hsa-miR-145 | -0.33299132 | 2.80E-07 |
| ANLN | hsa-miR-195 | -0.488127871 | 5.42E-15 |
| ANLN | hsa-miR-204 | -0.329193272 | 3.88E-07 |
| E2F3 | hsa-miR-145 | -0.469269979 | 7.85E-14 |
| E2F3 | hsa-miR-195 | -0.513166368 | 1.20E-16 |
| E2F3 | hsa-miR-204 | -0.34911661 | 6.60E-08 |
| EZH2 | hsa-miR-100 | -0.396241634 | 5.93E-10 |
| EZH2 | hsa-miR-143 | -0.399242488 | 4.28E-10 |
| EZH2 | hsa-miR-145 | -0.381328532 | 2.86E-09 |
| EZH2 | hsa-miR-195 | -0.440751439 | 3.31E-12 |
| FRMD5 | hsa-miR-195 | -0.345372701 | 9.29E-08 |
| NOVA1 | hsa-miR-141 | -0.511391444 | 1.58E-16 |
| NOVA1 | hsa-miR-17 | -0.51495273 | 9.00E-17 |
| NOVA1 | hsa-miR-182 | -0.502834725 | 5.99E-16 |
| NOVA1 | hsa-miR-183 | -0.519968961 | 4.01E-17 |
| NOVA1 | hsa-miR-200a | -0.547167328 | 3.97E-19 |
| NOVA1 | hsa-miR-21 | -0.434655524 | 7.06E-12 |
| NOVA1 | hsa-miR-210 | -0.522220489 | 2.78E-17 |
| NOVA1 | hsa-miR-222 | -0.586790734 | 2.14E-22 |
| NOVA1 | hsa-miR-301b | -0.317575503 | 1.03E-06 |
| NOVA1 | hsa-miR-503 | -0.455863477 | 4.76E-13 |
| NOVA1 | hsa-miR-93 | -0.528491667 | 9.88E-18 |
| NOVA1 | hsa-miR-96 | -0.482215319 | 1.27E-14 |
| SOX11 | hsa-miR-195 | -0.505998758 | 3.68E-16 |
